# Structural underpinnings of Ric8A function as a G-protein α-subunit chaperone and guanine-nucleotide exchange factor

**DOI:** 10.1038/s41467-019-11088-x

**Published:** 2019-07-12

**Authors:** Dhiraj Srivastava, Lokesh Gakhar, Nikolai O. Artemyev

**Affiliations:** 1 Department of Molecular Physiology and Biophysics, University of Iowa Carver College of Medicine, Iowa City, IA 52242 USA; 2 Department of Biochemistry, University of Iowa Carver College of Medicine, Iowa City, IA 52242 USA; 3 Protein Crystallography Facility, University of Iowa Carver College of Medicine, Iowa City, IA 52242 USA; 4 Department of Ophthalmology and Visual Sciences, University of Iowa Carver College of Medicine, Iowa City, IA 52242 USA

**Keywords:** X-ray crystallography, GTP-binding protein regulators, Molecular modelling

## Abstract

Resistance to inhibitors of cholinesterase 8A (Ric8A) is an essential regulator of G protein α-subunits (Gα), acting as a guanine nucleotide exchange factor and a chaperone. We report two crystal structures of Ric8A, one in the apo form and the other in complex with a tagged C-terminal fragment of Gα. These structures reveal two principal domains of Ric8A: an armadillo-fold core and a flexible C-terminal tail. Additionally, they show that the Gα C-terminus binds to a highly-conserved patch on the concave surface of the Ric8A armadillo-domain, with selectivity determinants residing in the Gα sequence. Biochemical analysis shows that the Ric8A C-terminal tail is critical for its stability and function. A model of the Ric8A/Gα complex derived from crosslinking mass spectrometry and molecular dynamics simulations suggests that the Ric8A C-terminal tail helps organize the GTP-binding site of Gα. This study lays the groundwork for understanding Ric8A function at the molecular level.

## Abstract (web summary)

Ric8A regulates G protein α-subunits (Gα) by acting as a guanine nucleotide exchange factor (GEF) and a Gα chaperone. Here, the authors solve the crystal structures of free and Gα fragment bound Ric8A, and provide insights into the structural basis for Ric8A’s GEF and chaperone functions.

## Introduction

G protein-coupled receptors (GPCRs) and their cognate heterotrimeric G proteins (Gαβγ) are the key components of a major cellular signaling pathway that is activated when agonist binds to the GPCR, enabling its interaction with Gαβγ and catalyzing the release of GDP from Gα. This enables Gα to bind GTP, triggering dissociation of the signaling species GαGTP and Gβγ, which modulate numerous downstream effectors. Thus, GPCRs serve as guanine nucleotide-exchange factors (GEFs) for G proteins. However, G proteins can also be activated by nonreceptor GEFs. Notable among these are the resistance to inhibitors of cholinesterase 8 (Ric8) proteins, which regulate G-protein biology in species ranging from slime molds to vertebrates and, in contrast to GPCRs, act on monomeric Gα subunits^1–5^. Whereas the genomes of invertebrates encode a single ancestral Ric8 isoform that is capable of interacting with Gα subunits of all types^6,7^, their vertebrate counterparts encode two isoforms, each of which regulates a particular subset of Gα subunits: Ric8A (Gα_i/t_, Gα_q_, and Gα_12/13_) and Ric8B (Gα_s_)^8–11^. The interaction of Ric8 with GDP-bound Gα stimulates release of GDP, leading to the formation of a stable intermediate complex of Ric8 and nucleotide-free Gα. Once Gα binds GTP it dissociates from Ric8, and thus the nucleotide-exchange cycle on Gα is completed^8,12^.

Although the GEF activity of Ric8A and Ric8B was initially thought to account for the ability of these proteins to positively regulate G-protein signaling, mounting evidence suggests that the Ric8 proteins can also serve as ubiquitous chaperones for Gα-subunits^13–15^. The fact that the chaperone function of Ric8 augments G-protein signaling could readily explain many, if not all, biological effects of the protein. Although it remains unclear whether the GEF or chaperone function of Ric8 proteins is predominant with respect to its biological effects, we reasoned that the structure of the Ric8/Gα complex might hold the key to understanding both. This complex underlies the GEF activity of Ric8 and may, in fact, be similar to the folding intermediate that is present during the biosynthesis of Gα^12^.

The mechanism underlying the ability of GPCRs to function as GEFs has been extensively investigated. Structures of GPCR/Gαβγ complexes have revealed, in atomic detail, GPCR-induced structural perturbations of Gα that lead to the release of GDP^16–19^. Gα subunits feature two key domains, the Ras-like domain (RD) and the α-helical domain (HD)^20^. The RD binds the nucleotide and interacts with the HD, with the latter serving as a lid over the nucleotide binding site. Agonist-bound GPCR engages Gαβγ at two key sites of Gα: the C-terminal α5 helix and the N-terminal αN-β1 loop. The largest GPCR-induced conformational change in Gα is an outward translation with rotation of the α5 helix that leads to a displacement of the guanine ring binding loop β6-α5 of Gα^16^. Changes associated with the interaction between the receptor and the αN-β1 loop propagate to and disturb the phosphate-binding P-loop (β1-α1). The dual disruption of the guanine-nucleotide binding site is accompanied by weakening of the RD/HD interface and stabilization of conformational states in which RD and HD are dynamically separated, facilitating the escape of GDP^16,21^.

In contrast to the wealth of structural information available regarding the interactions between GPCRs and G proteins, data at atomic level have not been available for Ric8 proteins, either alone or in complex with Gα. A lack of sequence similarity between Ric8 isoforms and other proteins has precluded homology modeling of its structure. However, protein-fold recognition algorithms have predicted that Ric8 adopts an armadillo-like fold^22^. The complete lack of structural relatedness between GPCRs and Ric8 suggests that the mechanisms whereby these GEFs activate G proteins are distinct. This notion is supported by the finding that Ric8 cannot interact with heterotrimeric Gαβγ^8^. It had been proposed that Ric8 might interact with the conformationally sensitive switch II region of Gα, which is occluded in the heterotrimer by Gβγ^8,23,24^. This interaction may also explain the selectivity of Ric8 for the GDP-bound state of Gα^8^. Notwithstanding these differences, notable parallels between G protein activation by Ric8A and GPCRs were suggested by some of the available biochemical data. Like GPCRs, Ric8A has been shown to induce separation of RD and HD of Gα, and to do so by perturbing the secondary structure surrounding the guanine-binding site within the RD^25^. A second parallel is that the key Ric8A interaction site of Gα has been localized to the C-terminal α5 helix^26^.

To gain mechanistic insights into the function of Ric8 and its interaction with Gα, we solve the crystal structures of both apo Ric8A and Ric8A in complex with the C-terminal fragment of transducin-α (Gα_t_) attached to a maltose-binding protein (MBP) tag. These structures reveal that Ric8A has two major modules: the core armadillo (ARM)-repeat domain, composed of 8 ARM repeats, and a flexible C-terminal tail. In addition, they show that the concave surface of the Ric8A armadillo domain encompasses a conserved binding site for the C-terminus of Gα. Our biochemical studies demonstrate that the C-terminal tail is important for the stability of the Ric8A protein overall, and key for its GEF function. Modeling of the Ric8A/Gα complex and flexible C-terminal tail of Ric8A provides a foundation for understanding the molecular mechanisms that underlie the stability, GEF function, and chaperone function of Ric8A.

## Results

### Ric8A binds the C-terminus of Gα with high affinity

The C-terminal 18-mer peptide of Gα_i_ corresponding to Gα_t_333–350, was previously shown to bind to Ric8A^26^. To identify C-terminal constructs of Gα that are suitable for crystallization with Ric8A, we tested one in which the 11 C-terminal residues of Gα_t_ were fused to the B1 domain of Streptococcal protein G (GB1-Gα_t_340–350) and a second in which the 24 C-terminal residues of Gα_t_ were fused to MBP (MBP-Gα_t_327–350). For Ric8A, we used a construct that is truncated at the C-terminus (Ric8A1–492) and that had been reported to retain the full functionality of Ric8A^26^. Elution profiles generated by size-exclusion chromatography (SEC) indicated that both fusion proteins formed complexes with Ric8A1–492 at nearly 1:1 stoichiometry (Supplementary Fig. 1), suggesting that the 11 C-terminal residues of Gα_t_ are sufficient for a high-affinity (submicromolar *K*_d_) interaction.

**Fig. 1:**
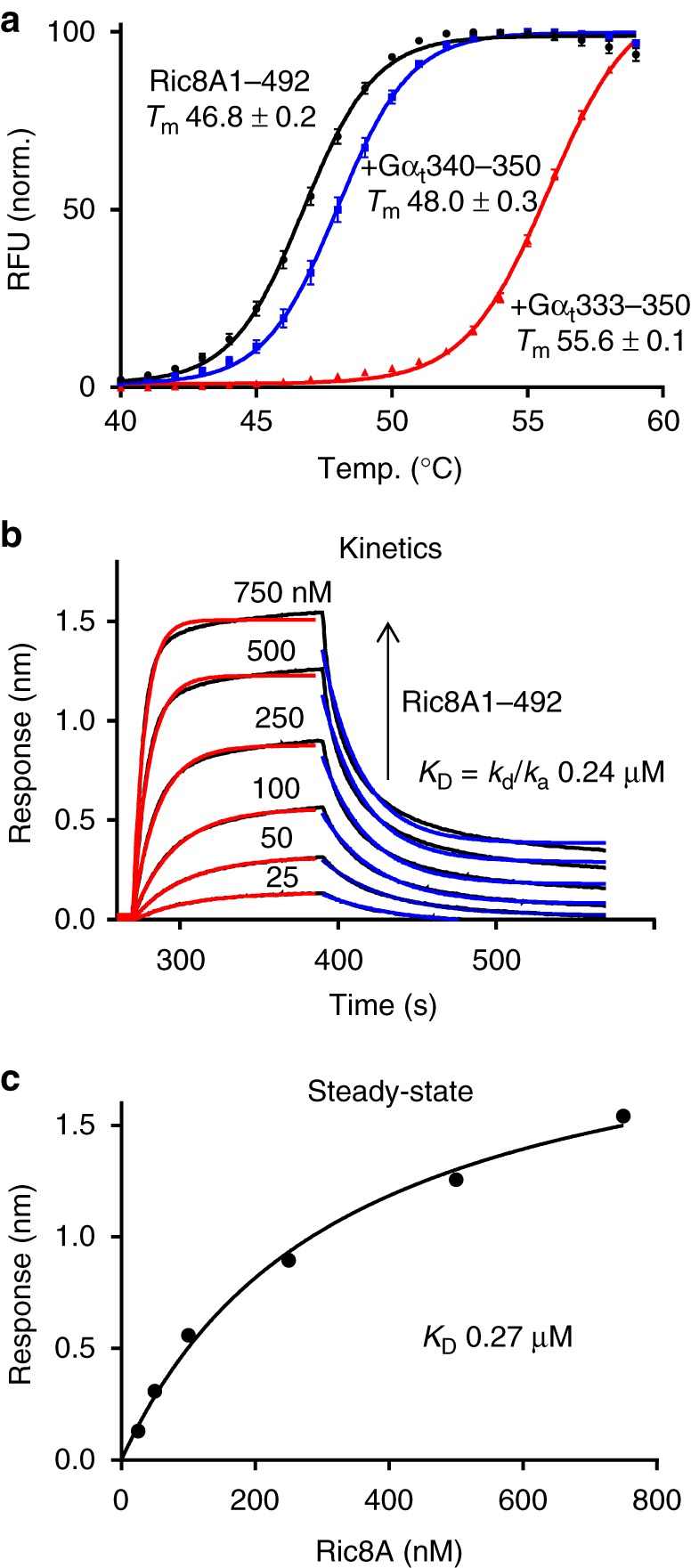
Interaction of Ric8A with the C-terminus of Gα. **a** Thermal denaturation, as determined by differential scanning fluorimetry (DSF), of 8.7 µM Ric8A1–492 in the absence (black) or presence of 50 µM Gα_t_340–350 (blue) or Gα_t_333–350 (red). RFU – relative fluorescence units. Average normalized curves are shown. The *T*_m_ values (°C) are shown as mean ± SD (*n* = 3). **b** Kinetics of association and dissociation for Ric8A1–492 and biotinylated Gα_t_333–350 coupled to a streptavidin biosensor as determined using BLI. Representative curves are shown. The processed data curves are black and the nonlinear regression fits from the 1:1 binding model are red (association; *k*_a_ = 1.3 ± 0.3 × 10^5^ M^−1^s^−1^) and blue (dissociation; *k*_d_ = 0.031 ± 0.008 s^−1^) (mean ± SD). **c** The steady-state binding curve obtained from data in (b); *K*_D_ = 0.27 μM. For *n* = 4 experiments, *K*_D_ = 0.28 ± 0.07 μM (mean ± SD). Source data are provided as a Source Data file

Next, we evaluated the effects that binding of the 11-mer and 18-mer C-terminal peptides of Gα_t_ (Gα_t_340–350 and Gα_t_333–350, respectively) had on the thermal stability of Ric8A1–492. To this end, we used differential scanning fluorimetry (DSF) (Fig. 1a). These tests revealed that Gα_t_333–350, but not Gα_t_340–350, had a strong stabilization effect (~9 °C shift) on Ric8A1–492 (Fig.1a). We infer from the protein stabilization effect that the longer peptide more fully recapitulates the C-terminal interaction of Gα_t_ with Ric8A. The kinetics and affinity of binding for Gα_t_333–350 and Ric8A1–492 were quantitated using biolayer interferometry (BLI), with N-terminally biotinylated Gα_t_333–350 attached to a streptavidin biosensor. The binding and dissociation kinetics for the Ric8A1–492/Gα_t_333–350 interaction were consistent with a 1:1 binding model, with the average association constant (*k*_a_) measured at 1.3 ± 0.3 × 10^5^ M^−1^s^−1^ (mean ± SD) and the average dissociation constant (*k*_d_) at 0.031 ± 0.008 s^−1^, yielding a *K*_D_ = *k*_d_/*k*_a_ of 0.24 µM (Fig. 1b). The *K*_D_ calculated based on steady-state analysis of the interaction between Ric8A1–492 and Gα_t_333–350 was comparable (*K*_D_ = 0.28 ± 0.07 μM, mean ± SD, *n* = 4 (Fig. 1c). Our analysis indicates that the interaction between Ric8A and the Gα C-terminus has about 45-fold higher affinity than previously estimated (*K*_D_ 12 µM)^26^.

### Ric8A is an ARM-repeat protein with a flexible C-terminus

**Fig. 2:**
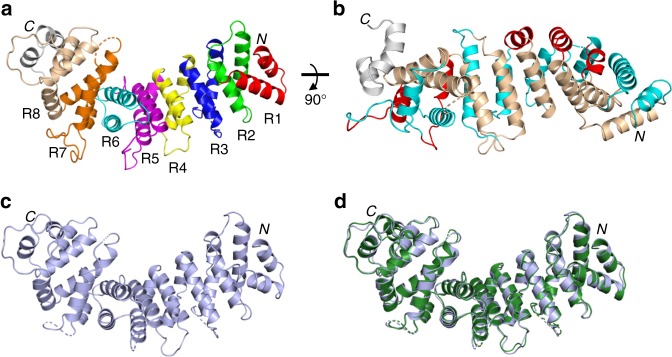
Structure of the Ric8A armadillo core. Structure of Ric8A1–426 based on the Ric8A1–492/MBP-Gα_t_327–350 complex. **a** ARM repeats R1–8 are shown in different colors, as indicated. **b** α-helices h1, h2, and h3 comprising each ARM-repeat are shown in red, cyan, and wheat, respectively. The two-helix hairpin of Ric8A (residues  400–426) is shown in gray. **c** Structure of apo Ric8A1–492 in which only residues 1–422 were resolved. **d** Overlay of the structures in (**a**) and (**c**)

Crystallization attempts using human Ric8A1–492 in the absence or presence of Gα_t_333–350 peptide or 24 C-terminal residues of Gα_t_ fused to MBP (MBP-Gα_t_327–350) were unsuccessful. However, the use of bovine Ric8A1–492 with an N-terminal His6-tag led to well-diffracting crystals of the protein in complex with MBP-Gα_t_327–350 (Supplementary Table 1, Supplementary Fig. 2). Residues at positions 1–426 of Ric8A other than those in the flexible loops (102–106, 202–207, and 292–303) could be modeled clearly based on the electron density (Fig. 2a, b). The electron density for residues 427–492 was missing despite the confirmed presence of unproteolysed Ric8A1–492 in the crystal (Supplementary Fig. 3).

**Fig. 3:**
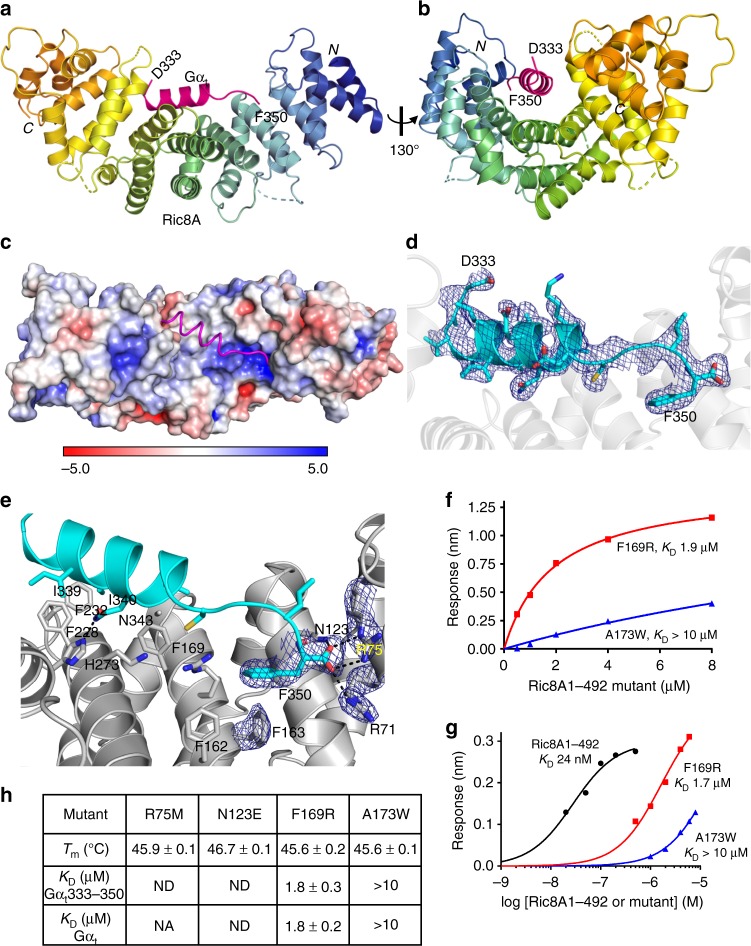
Interface of Ric8A with the C-terminus of Gα_t1_. **a** View of Gα_t_333–350 (magenta) bound to the concave surface of Ric8A (rainbow), based on the structure of the Ric8A1–492/MBP-Gα_t_327–350 complex. **b** View highlighting curvature of the concave binding surface of Ric8A. **c** Electrostatic surface representation of Ric8A with the bound Gα_t_333–350 (magenta tube), showing helical residues Gα_t_335–346 interacting with the neutral surface and the C-terminal-most residues contacting the positively charged patch (units *K*_*b*_*T*/*e*_c_). **d** An omit map (*F*o-*F*c) for Gα_t_333–350 is contoured at 2.5σ and shown for 1.6 Å around the ligand. **e** Close-up of the interface between Ric8A (gray) and the Gα_t_ C-terminus (cyan). Omit maps (*F*o-*F*c) for Gα_t_ F350 and its critical Ric8A contact residues R71, R75, N123, and F163 are contoured at 2.5σ and shown for 1.6 Å around the side chains. **f**, **g** Properties of the mutations that alter Ric8A/Gα_t_333–350 interface. Representative steady-state binding curves from BLI assays measuring the binding of Ric8A1–492 mutants F169R and A173F to biotinylated Gα_t_333–350 (**f**) or Ric8A1–492 and its F169R and A173F mutants to Avi-tagged Gα_t_ (**g**) coupled to a streptavidin biosensor. **h** Table showing thermostability (DSF assay) and Gα_t_333–350 and the full-length Gα_t_ steady-state binding affinities of Ric8A1–492 mutants calculated from the BLI assays (mean ± SD, *n* = 3). ND – not detectable. NA – not available. Source data are provided as a Source Data file. Stereo images of 3d and 3e are available in Supplementary Fig. 7

Apo Ric8A1–492 was subsequently crystallized, and the molecular replacement solution for this crystal structure was found using the structure of Ric8A in complex with MBP-Gα_t_327–350 (Supplementary Table 1, Fig. 2c). As in the case for Ric8A within the complex, only a subset of the Ric8A1–492 residues (Ric8A1–422) could be modeled based on the electron density, suggesting that the C-terminal tail of Ric8A is flexible. Furthermore, comparison of the two structures shows that the binding of MBP-Gα_t_327–350 to Ric8A does not cause major conformational changes to its core domain, Ric8A1–422 (Fig. 2d). In both structures, ~400 N-terminal residues of Ric8A were packed into a classical armadillo fold consisting of eight ARM repeats (R1–R8; Fig. 2a, b)^27^. R3, 4, 5, 7, and 8 have a canonical ARM repeat structure containing three α-helices, with one short (h1) and running nearly perpendicular to a hairpin formed by two longer antiparallel helices h2 and h3^28^. R1 is a partial ARM repeat that lacks h1, and R2 and R6 appear to be non-canonical ARM repeats, with h1 replaced by a loop. The tandem packing of ARM repeats produces a right-handed ribbon-like superhelix featuring a concave surface formed by the h3 α-helices from R2-R8 (Figs. 2b and 3a, b).

Comparing the Ric8A1–426 structure to those of other proteins in the Protein Data Bank using DALI^29^ revealed structural homology with functionally diverse armadillo-fold proteins including β-catenin, importin-α, and the RhoA/RhoC GEF SmgGDS (Supplementary Fig. 4A–C). However, the limited structural similarity (root mean square deviation, RMSD > 3 Å) does not extend beyond ~400 N-terminal residues of Ric8A. Although residues 400–422 of Ric8A form a two-helix hairpin, their orientation and packing differ from those that characterize the armadillo fold (Fig. 2). Moreover, in the structures of apo Ric8A1–492 and Ric8A1–492/MBP-Gα_t_327–350 conformations of the hairpin are nearly identical despite the different crystal packing interactions that involve residues 400–422 (Supplementary Fig. 5). This suggests that the contacts within the crystal lattice are not responsible for termination of the armadillo fold after R8. Thus, Ric8A contains 8 ARM repeats rather than the 10 that were initially predicted^22^. The apparent structural flexibility of Ric8A427–492 is consistent with its secondary structure prediction (Supplementary Fig. 6).

### The Gα_t_ C-terminus binds to the concave surface of Ric8A

The crystal structure of the Ric8A1–492/MBP-Gα_t_327–350 complex revealed an interface that buries 956 Å^2^ of surface area (total buried area divided by 2)^30^. Residues Gα_t_333–350 contact the concave surface of Ric8A from R2 to R8 (Fig. 3a, b). The Gα_t_ C-terminus is not directly involved in crystal contacts. Residues Gα_t_335–346 form an α-helix that rests over a neutral surface of Ric8A, whereas Gα_t_347–350 is in an extended conformation and lies over a positively charged surface (Fig. 3a, c). In the final refined structure, all of the side chains of residues Gα_t_333–350 were modeled with confidence (Fig. 3d, e, Supplementary Fig. 7). The C terminal carboxylate group of Gα_t_ F350 forms a hydrogen bond and polar interactions with the side-chain amide of N123, as well as the guanidino groups of R71 and R75 of Ric8A (Fig. 3e). The backbone nitrogen atom of F350 also form a hydrogen bond with an oxygen of the side-chain amide of N123. Furthermore, the side-chain of F350 is involved in the T-shaped *π*–*π* stacking interaction with F163 of Ric8A, which is held in place by the T-shaped *π*–*π* stacking interaction with the neighboring F162 (Fig. 3e). Another hydrogen bond is formed between the side chains of Gα_t_ N343 and Ric8A H273. Besides these hydrogen bonds and polar interactions, most of the interactions between Ric8A and the Gα_t_ C-terminus are hydrophobic or side-chain packing interactions. For instance, I339 and I340 of Gα_t_ occupy a hydrophobic patch formed by F228 and F232 of Ric8A (Fig. 3e). By binding across the concave surface of Ric8A, the Gα_t_ C-terminus could act as a scaffolding element that bridges multiple ARMs. Such a scaffolding interaction could underlie the increase in the thermal stability of Ric8A1–492 in the presence of the Gα_t_333–350 peptide.

To probe the protein interface observed in the crystal structure we tested the ability of Gα_t_333–350 to bind to Ric8A1–492 proteins that were mutated to disrupt this interaction (Fig. 3f, Supplementary Fig. 8). The substitutions, R75M and N123E, were predicted to interfere with the interaction network of Gα_t_ F350. These mutations had the most severe consequences, as they ablated the binding of Ric8A1–492 to Gα_t_333–350 in the BLI assay. The substitutions, F169R and A173F, introduced steric hindrance, which also severely impaired the interaction (Fig. 3f). Similar results were obtained using the full-length Avi-tagged Gα_t_ in the BLI assay. Ric8A1–492 bound to Gα_t_ with high affinity (*K*_D_ 24 ± 2 nM, mean ± SD, *n* = 3; Fig. 3g, Supplementary Fig. 9A). The F169R and A173F mutations severely reduced affinity of Ric8A1–492 for Gα_t_, whereas no Gα_t_ binding was detected using the N123E mutant (Fig. 3g, h; Supplementary Fig. 9B). In addition, the disruption of the interaction of Ric8A with the full-length Gα_t_ was observed using the Ric8A R75M mutant in SEC co-migration experiments (Supplementary Fig. 10). Thus, mutational analysis validated the Gα_t_ binding site on Ric8A identified in our crystal structure.

To assess the conformation of the C-terminal region of Gα in the absence of intramolecular interactions we solved the crystal structure of MBP-Gα_t_327–350 alone (Supplementary Table 1). Although the Gα_t_327–348 sequence could be traced in electron density, it largely lacked secondary structure and the conformation differed markedly from that in the complex containing Ric8A (Supplementary Fig. 11). Thus, binding of Gα_t_327–350 by Ric8A induces the formation of an α-helix by Gα_t_335–346 and/or stabilizes such a structure.

### The Gα C-termini contribute to the Ric8 isoform selectivity

**Fig. 4:**
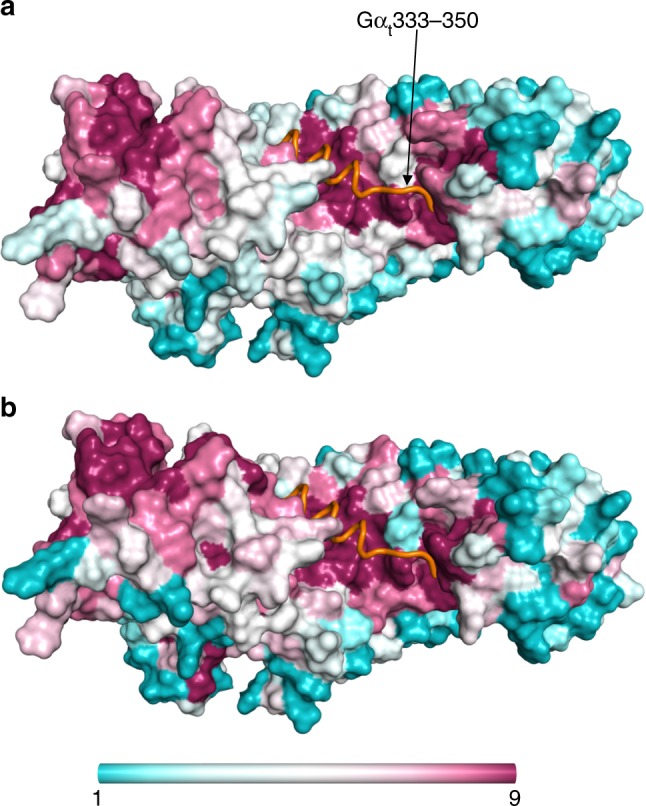
Binding surface for the Gα_t_ C-terminus is highly conserved in Ric8 isoforms. Surface representations for Ric8A1–426 colored by residue conservations scores derived from ConSurf analysis of a sample of 250 Ric8 homologs (**a**) and 117 Ric8A orthologs (Ric8A subtree) (**b**). The sequences for the analysis with maximal identity of 97% and minimal identity of 30% were collected from the UniProt database. Color scale: 9 – magenta – conserved, 1 – cyan –variable. Gα_t_333–350 is shown as an orange tube

The interface between Ric8A and the Gα_t_ C-terminus involves both residues that are conserved across all families of Gα subunit (such as Gα_t_ I339, L344, and L349) and residues that are conserved only in the Gα_i/t_ family (V335, T336, I340, C347, and F350)^31^. In contrast, nearly all of the Ric8 residues that interact with Gα are strongly conserved across the Ric8A and Ric8B isoforms, as well as the ancestral Ric8 proteins. (Fig. 4, Supplementary Data 1)^32^. The conservation analysis, therefore, suggests that family-specific residues of Gα, but not of Ric8 proteins, are the determinants of selective coupling between these proteins. We tested this notion, using BLI to examine the binding of Ric8A to a peptide comprised of the C-terminal 18 residues of Gα_s_ (Gα_s_363–380). Notably, Ric8A was capable of binding to Gα_s_363–380, albeit with a ~18-fold lower affinity than to Gα_t_333–350 (Supplementary Fig. 12a–c).

Examination of the Ric8A/Gα_t_327–350 interface in the crystal structure suggested that many of the contacts would be maintained if Gα_t_ residues were substituted for their Gα_s_ counterparts. However, we considered that the four C-terminal Gα_t_ residues, CGLF, may provide a better fit for the extensive packing interactions with Ric8A. We therefore tested a chimeric C-terminal peptide of Gα_s_ ending in CGLF (peptide C1) (Supplementary Fig. 12a, d, e). Although the binding of C1 to Ric8A was not enhanced, a peptide containing two additional substitutions, Gα_s_Q370I (I340 in Gα_t_) and Gα_s_H373N (N343 in Gα_t_) (peptide C2), resulted in potent Ric8A binding, similar to that for Gα_t_333–350 (Supplementary Fig. 12a, f, g). Moreover, the double I340Q/N343H mutation of Gα_t_ decreased its affinity for Ric8A1–492 by about 100-fold (*K*_D_ = 2.3 ± 0.4 µM, mean ± SD, *n* = 3; Supplementary Fig. 13). These findings suggest that residues at positions I340 and N343 of Gα_t_ are important for the selectivity of interactions between Gα_i/t_ proteins and Ric8A. Regions of Gα other than the C-termini may also be involved in the selective interaction with Ric8 isoforms given that the Gα_s_ C-terminal peptide interacted with Ric8A to some extent.

The strong conservation of the Gα C-terminal binding surfaces in Ric8A and Ric8B raised the possibility that Ric8B interacts with the C-termini of Gα_i/t_ -like Gα subunits. However, no interaction between Gα_t_333–350 and Ric8B was detected, even when a high concentration of Ric8B (20 µM) was used. Hence, the conformation of the Ric8B site that binds the Gα C-terminus may differ from the one on Ric8A.

### The Ric8A stability is regulated by its proximal C-terminus

The structure of the Ric8A1–492/MBP-Gα_t_327–350 complex indicates that the flexible C-terminal region Ric8A427–492 is not directly involved in binding the Gα C-terminus. However, this region is known to contain a potential Gα binding site^33^. To determine the role of Ric8A427–492 in the function of this protein, we first used BLI to assess an interaction between Ric8A1–426 (lacks the C-terminal region) and Gα_t_333–350. Unexpectedly, the Gα_t_ C-terminal peptide did not bind to Ric8A1–426 (Supplementary Fig. 14A). To probe the mechanism whereby Ric8A427–492 contributes to the interaction with Gα_t_333–350, we examined the thermal stability of Ric8A1–426 by DSF. This analysis revealed that Ric8A1–426 (*T*_m _= 36.5 ± 1.1 °C, mean ± SD, *n* = 3) was much less stable than Ric8A1–492 (Supplementary Fig. 14B). Thus, the C-terminal tail of Ric8A seems to be engaged in intramolecular interactions with the core domain, Ric8A1–426, both stabilizing it and allosterically promoting its binding to the Gα C-terminus.

**Fig. 5:**
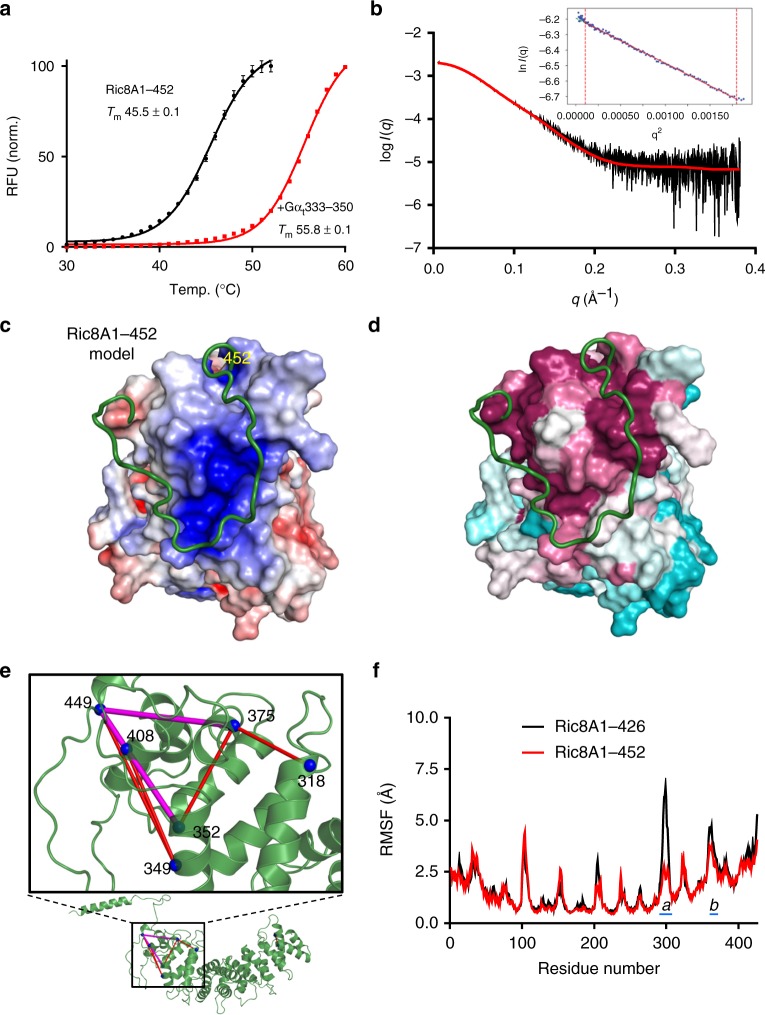
Proximal portion of the Ric8A C-terminal tail binds and stabilizes the armadillo core domain. **a** Denaturation of 9.1 µM Ric8A1–452, as determined by DSF, in the absence (black) or presence of 50 µM Gα_t_333–350 (red). RFU – relative fluorescence units. The *T*_m_ values (°C) are shown as mean ± SD. **b** Experimental SAXS data for Ric8A1–452 (black). Theoretical SAXS profile calculated for the Ric8A1–452 model fits the SAXS data with a *χ*^2^ value of 1.82. The Guinier plot for the low q region (*q*∙*R*_g_ < 1.3) is shown in the inset. **c**, **d**. The best-scoring FloppyTail model of Ric8A1–452 from cluster I. The proximal C-terminal region Ric8A423–452, shown as a ribbon, the core domain Ric8A1–422 shown as electrostatic surface representation (units *K*_*b*_*T*/*e*_c_) (**c**) or as a conservation surface (based on ConSurf analysis Ric8 homologs (**d**); the color scale represents conservation scores, 9 – magenta – conserved, 1 – cyan - variable). **e**. Close-up of the model of apo Ric8A1–492 showing the region with the highest concentration of intramolecular DSS-crosslinks. Eight of the total nine crosslinks (blue) were identified in the C-terminal half of Ric8A. Crosslink K352/K449 is obscured by crosslinks K352/K408 and K408/K449. All the crosslinks satisfy the distance threshold of 30 Å^74^. Crosslinks K352/K449 and K408/K449 used in FloppyTail modeling of Ric8A1–452 and crosslink K375/K449 used in model selection are colored in magenta; all other crosslinks are in red. **f** RMSF plots averaged from four MD simulations (Supplementary Fig. 18) for each Ric8A1–426 (black) and the model of Ric8A1–452 (red). The flexible regions **a** and **b** of Ric8A significantly stabilized by the proximal part of its C-terminal tail are indicated with a blue line. The peak average RMSF values (Å) for **a** (6.7 ± 1.4 vs. 2.1 ± 0.4 (mean ± SE, *n* = 4), unpaired *t-*test *P = 0.02, residue 299) and **b** (4.7 ± 0.2 vs. 3.7 ± 0.1, unpaired *t-*test **P = 0.004, residue 363) are statistically different. Source data are provided as a Source Data file

To map these intramolecular interactions, we generated the Ric8A construct, Ric8A1–452, in which less of the C-terminal region is missing. Both the thermal stability of Ric8A1–452 and its ability to bind to and be stabilized by Gα_t_333–350 were comparable to those of Ric8A1–492, indicating that proximal C-terminal residues 427–452 are essential (Fig. 5a, Supplementary Fig. 15). This segment is conserved in the Ric8A and Ric8B isoforms, and it is also highly acidic.

To identify intramolecular interaction sites involving the C-terminal region of Ric8A1–492 we utilized the crosslinking mass spectrometry (XL-MS) approach, with disuccinimidyl suberate (DSS) serving as the crosslinking agent. Nine crosslinked pairs were identified using the Protein Prospector software^34^. Among these pairs, four involved K449 in the flexible C-terminal region (Supplementary Data 2, Fig. 5e). In particular, K449 was crosslinked to K349, K352, K375, or K408. Residues K349, K352, and K408 together with R345, R348, and R405 form a large and highly conserved positively charged patch on the outer surface of the C-terminal portion of the Ric8A core domain (Fig. 5c, d, Supplementary Fig. 5D). Thus, the crosslinking analysis is consistent with the notion that electrostatic interactions between the negatively charged proximal C-terminal segment Ric8A427–452 and the positively charged surface on the Ric8A core domain play an important role in stabilizing Ric8A.

### A model of Ric8A C-terminus accounts for its stabilization

We employed the FloppyTail application within the Rosetta framework to model the structure of the C-terminal region of Ric8A in its apo form and in complex with Gα^35^. First, we modeled the structure of the proximal portion of the Ric8A C-terminal tail. A total of 4900 models of Ric8A1–452 were generated, from which 212 models were selected based on the energy scores, crosslinking constraints and agreement (*χ*^2^ values) between theoretical SAXS profiles for models and the experimental SAXS profile of Ric8A1–452 (see Methods, Fig. 5b, and Supplementary Table 2, Supplementary Fig. 16). Clustering of 212 models yielded two main clusters I and II that were comprised of 50 and 20 models, respectively (Supplementary Fig. 17)^36^. In all of the highest scoring models, the basic patch in the core Ric8A1–426 and the acidic stretch Ric8A436–444 (EDEDTDTDE) were in close proximity and shared contacts. Cluster I included 5 of the top 10 energy score models. Cluster II models were rejected because they predicted that the orientation of the C-terminal end was away from the Gα-binding site, making the reported interaction of the Ric8A C-terminal tail with Gα highly improbable (Supplementary Fig. 17)^33^. Thus, the highest scoring model of Ric8A1–452 from cluster I was selected for further analysis and modeling (Fig. 5c, d).

Molecular dynamics (MD) simulations of the Ric8A1–452 model were performed to determine if and how it might account for the higher thermal stability of Ric8A1–452 vs. Ric8A1–426. MD simulations of the Ric8A1–426 structure and the Ric8A1–452 model revealed that the root mean square fluctuation (RMSF) values for residues in the mobile regions are significantly reduced in the Ric8A1–452 model, indicating that the protein is stabilized (Fig. 5f, Supplementary Fig. 18).

The conformation of the distal portion of the C-terminal tail (Ric8A453–492) was assessed using the experimental SAXS profile of Ric8A1–492 and a conformational sampling analysis using the BILBOMD server^37^ (Supplementary Figs. 16 and 19). The best-scoring single state and three-state models of apo Ric8A1–492 suggest that the distal portion of the C-terminal tail assumes extended conformations and does not contact the core of the protein (Supplementary Fig. 19).

### Mechanistic insights from modeling of the Ric8A/Gα complex

Our initial model of the Ric8A/Gα complex was generated by superimposing the α-helix (residues Gα_t_335–340) from Gα_t_GDP (PDB 1TAG) and the Ric8A1–492/MBP-Gα_t_327–350 structure (PDB 6N85). This overlay revealed extensive overlap and clashes between Gα and the C-terminal half of Ric8A, suggesting that major conformational changes are needed to accommodate this interaction (Supplementary Fig. 20A). A similar overlay using the Gα conformation from the GPCR/Gα structure (PDB 3SN6) significantly reduced these clashes, and thus was selected for further modeling (Supplementary Fig. 20B)^16^. To simplify the modeling, we took advantage of the functionality of reduced miniGα constructs, which lack the HD domain^38,39^. A homology model of miniGα_i_ lacking the αN-helix (ΔN25-miniGα_i_) was generated using a structure of the GPCR-bound miniGα_s_^38^. MD simulations of the of Ric8A1–452 model (Supplementary Fig. 18) and ΔN25-miniGα_i,_ (Supplementary Fig. 21) allowed us to select conformations of these molecules that minimized the steric clashes. To simulate the forces that acted on Ric8A upon binding of ΔN25-miniGα_i_, we conducted steered MD (SMD) simulations (Supplementary Fig. 22). This SMD simulation led to a slight twisting of the C-terminal module and produced an open conformation of Ric8A1–452 with reduced concave curvature (Supplementary Fig. 20C). Ric8A1–452 in open conformation would not clash with ΔN25-miniGα_i_ upon superimposition of the C-terminal helical segments (Supplementary Fig. 20D).

**Fig. 6:**
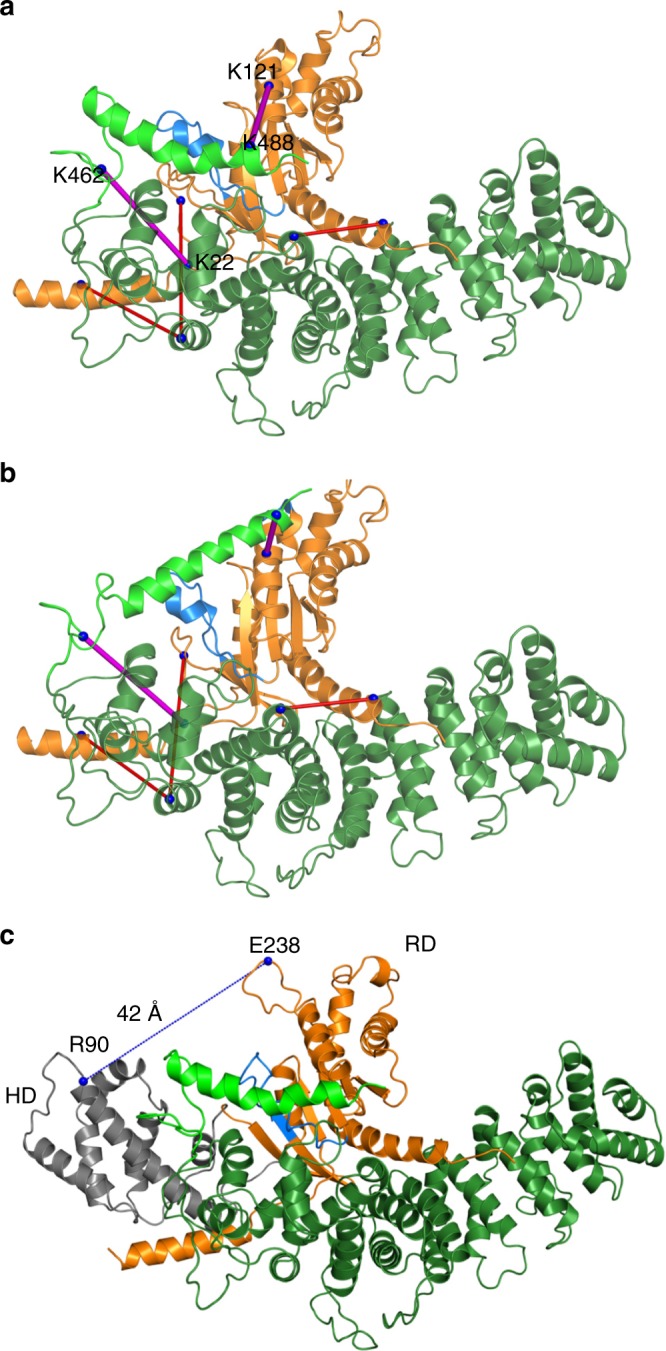
Models of the Ric8A/Gα complex. **a** FloppyTail model 1 from cluster I of Ric8A1–492/ΔN19-miniGα_i_ models with the addition of 19 N-terminal residues of miniGα_i_. Ric8A is shown in green and ΔN19-miniGα_i_ is shown in orange with the switch II region shown in blue. Distal C-terminal tail residues 455–470 extend beyond the C-terminal end of the Ric8A core domain, and the Ric8A 471–490 helix (light green) is situated along the switch II region (blue) and the α3-β5 loop. Five intermolecular crosslinks between Ric8A1–492 and miniGα_i_ were identified by XL-MS analysis and Protein Prospector^61^. All of the crosslinks satisfy the distance threshold of 30 Å^74^. The crosslink pairs that involve the distal portion of the Ric8A C-terminal tail and that were used in modeling (K488/miniGα_i_-K122 and K462/miniGα_i_-K21) are shown in magenta; all other crosslinks are in red. **b** FloppyTail model 2 from cluster II of Ric8A1–492/ΔN19-miniGα_i_ models. The Ric8A 471–490 helix (isolated light green helix) hangs over the switch II region (blue) and points towards the switch III region. The crosslinks are shown as in (**a**). **c** Model of the Ric8A1–492/Gα_i_ complex with a 42-Å separation of the α-helical domain (HD, gray) and Ras-like domain (RD, orange) of Gα_t_

Our biochemical assays confirmed an earlier report that deletion of the distal portion of the Ric8A C-terminal tail severely diminishes the GEF activity of this protein (Supplementary Fig. 23)^26^. This and the identification of a potential Gα-binding site at residues Ric8A455–470^33^ suggest that this portion of the C-terminal tail is essential for its functional interaction with Gα. To characterize this interaction, we conducted XL-MS on purified complexes of Ric8A1–492 with miniGα_i_ (Supplementary Data 3) and Gα_t_ (Supplementary Data 4, Supplementary Fig. 24). Gα_t_ and miniGα_i_ shared intermolecular crosslinks with the core domain of Ric8A (Fig. 6a), all of which were consistent with the Ric8A1–452/ΔN25-miniGα_i_ model (Supplementary Fig. 20D). Importantly, the crosslinking of Ric8A1–492 with Gα_t_ and miniGα_i_ identified one and two, respectively, crosslinked pairs involving the distal portion of the Ric8A C-terminal tail: Ric8A-K488/Gα_t_-K244, Ric8A-K488/miniGα_i_-K122 (equivalent to Gα_t_-K244), and K462/miniGα_i_-K21 (equivalent to Gα_t_-K25) (Fig. 6a, Supplementary Data 3 and 4). The latter two crosslinked pairs were used to constrain the FloppyTail modeling of Ric8A453–492. To allow the K462/miniGα_i_-K21 crosslink constraint to be used in the modeling, the N-terminus of the ΔN25-miniGα_i_ was extended to ΔN19-miniGα_i_.

In total, 5548 FloppyTail models were generated, clustered and sorted according to their energy scores (Supplementary Fig. 16). The two main clusters obtained using an RMSD value of 2.5 Å contained 14% (cluster I) and 11 % (cluster II) of models (Supplementary Fig. 25). The mean values for the energy score were −1652 for cluster I and −1657 for cluster II. In the majority of models, the distal C-terminal tail residues 455–470 rise above the C-terminal end of the Ric8A core domain, in proximity to the switch II region of Gα. In cluster I models, the Ric8A 471–490 α-helix is situated along the switch II region and the α3-β5 loop, whereas in cluster II models it hangs over the switch II region, pointing towards the switch III region. (Supplementary Fig. 25, Fig. 6a, b). Both the top cluster I and II models, exemplified by model 1 (energy score −1693) and model 2 (energy score −1690), respectively, were ranked high with respect to energy scores (Fig. 6a, b). Both models are plausible and do not differ in critical respects.

A model of the Ric8A1–492/Gα_i_ complex was generated based on model 1 of the Ric8A1–492/miniGα_i_ complex and the structure of the rhodopsin/G_i_ complex (Fig. 6c)^40^. The distance distribution 20–48 Å for the spin pair at residues 90 and 238 in Gα_i_ has been experimentally determined for the Ric8A/Gα_i_ complex as a measure of the degree of HD/RD separation^25^. In the model of the Ric8A1–492/Gα_i_ complex the distance between Cα atoms of Gα_i_ R90 and E238 is 42 Å. Furthermore, the HD in the model is free to sample all conformations with the reported distance distribution, and it does not appear to come into proximity with Ric8A.

## Discussion

In this study, we solved the structures of apo Ric8A alone and in complex with a fusion protein containing the Gα_t_ C-terminus and MBP. These structures revealed that the core domain of Ric8A has an armadillo-type fold comprised of 8 ARM repeats and that the highly conserved area on the concave surface of the Ric8A superhelical armadillo array serves as a binding site for the C-termini of Gα proteins. Additional binding experiments revealed that the selectivity of the Ric8A/Gα interaction is determined, in part, by a few class-specific residues of Gα, particularly those at positions corresponding to I340 and N343 of Gα_t_. Armadillo proteins that are structurally related to Ric8A, including SmgGDS β-catenin, and importin-α, commonly bind their partners at the concave surface^41–43^. However, the nature of the ligand-binding interactions of Ric8A differs from those of other ARM-repeat proteins (Supplementary Fig. 26).

Solving the structure of Ric8A1–426 in complex with the C-terminus of Gα_t_ allowed us to model the entire Ric8A/Gα complex because we could superimpose the Gα_t_ helical fragment from the complex with the α5 α-helix of the Gα subunit in the GDP, GTP, and GPCR-bound conformations. The significant reduction in clashes between Gα and Ric8A when the GPCR-bound conformation of Gα was used was intriguing. It suggested that, on binding to Ric8A and GPCR, the initial conformational changes of the Gα subunit are similar and involve α5 translation along, and rotation around, the helical axis, i.e., movement away from the GDP-binding site^16^. This distal conformation of α5 is rare in GαGDP, but it is dominant in complexes between agonist-bound GPCRs and Gαβγ^21^. Our finding that Ric8A is capable of high-affinity binding to a region as small as the 11-C-terminal residues of Gα_t_ suggests that Ric8A may also shift the equilibrium in GαGDP to the distal α5 conformation. Furthermore, the Ric8A-bound α5-helix of Gα displays an extension similar to that observed during the Gα C-terminal disorder-to-order transition when Gαβγ binds to GPCRs (Supplementary Fig. 11B)^16,44^.

Modeling of the GPCR-bound conformation of Gα in the complex with Ric8A did not prevent all clashes; further conformational changes in Ric8A were needed for this. SMD simulations indicated that Ric8A also assumes an open conformation to accommodate Gα. The resulting model of the Ric8A/Gα complex is consistent with our XL-MS analysis, and it indicates that in addition to binding the α5-helix of Gα, the Ric8A core domain extensively interacts with the αN-β1 loop and the switch II region of Gα. These interactions disrupt the Gα GDP-binding site and separate of the HD and RD domains^25^. Although the αN-β1 loop region is a part of the interface between Gαβγ and GPCRs, the interaction of the switch II region of Gα would be unique for the Ric8A/Gα complex. Furthermore, the modeled Ric8A/Gα interface is incompatible with binding of Ric8A to heterotrimeric G proteins.

Our studies have revealed critical roles for the seemingly unstructured C-terminal region of Ric8A (residues 427–492). The proximal portion of the Ric8A C-terminal tail (residues 427–452) was found to be important for stability of the protein. Further our XL-MS studies and molecular modeling suggest that the negatively charged stretch within Ric8A427–452 interacts with the conserved positively charged region of the Ric8A armadillo core. MD simulations confirmed that this interaction can markedly stabilize the most flexible regions of Ric8A. The finding that, in the crystal structure, the Gα C-terminus is bound to the Ric8A armadillo core despite the fact that the C-terminal tail of Ric8A lacks electron density appears to be serendipitous. In the crystal lattice, the Ric8A core is stabilized by contacts between its positively charged region and the negatively charged domain of MBP (Supplementary Fig. 5), which may displace the C-terminal tail. Phosphorylation of S435 and T440 of Ric8A by the protein kinase CK2 has been recently shown to enhance Ric8A binding to Gα subunits along with increasing its GEF and chaperone activities^45^. Our results suggest that instead of directly a being part of the Gα binding site, these phosphorylation sites augment the stabilizing intramolecular electrostatic interactions in Ric8A and thereby potentiate Ric8A activities.

Our analysis supports the idea that the C-terminal tail of Ric8A, Ric8A453–492, plays a central role in the GEF activity of the protein. It organizes or stabilizes regions of Gα that are disordered upon GDP dissociation, thereby enabling Gα to bind GTP. As has been shown biochemically, truncated Ric8A1–453 retains substantial capacity to dissociate GDP from Gα, yet Ric8A1–453-bound Gα is unable to bind GTP^26^. Furthermore, compelling evidence points to residues Ric8A455–470 being a binding site for Gα, and to the switch II region of Gα being an important binding site for Ric8A^8,33^. Our models of the position and conformation of the distal portion of the Ric8A C-terminal tail are consistent with existing biochemical evidence. In apo Ric8A, this part of the tail appears to extend away from the protein core. In contrast, in the Ric8A/Gα complex it reaches towards Gα and engages the switch II and, possibly, switch III regions, which are likely to be essential for the ability of Gα to bind GTP. Once GTP is bound, the switch regions change conformation, preventing Gα from binding to the distal portion of the Ric8A tail, and the interaction between Gα and the armadillo core is diminished by retraction of the α5 α-helix, resulting in dissociation of GαGTP from Ric8A.

Overall, the structure-based model of the Ric8A/Gα complex presented here suggests parallels, as well as differences, in the mechanisms underlying the GEF activities of GPCRs and Ric8A. Specifically, GPCRs and Ric8A engage some of the same regions of Gα, the C-terminus and the αN-β1 region^16^. Yet the Ric8A/Gα interface appears to be more extensive and Ric8A seems to induce more profound structural perturbations of Gα near the switch II region and guanine-nucleotide binding site^33^. Thus, Ric8A appears to use the distal portion of its tail to organize, or form de novo, the guanine-nucleotide binding site on Gα, as this would be required for its chaperone activity and GTP-binding.

## Methods

### Protein expression and purification

For crystallography and biochemical studies, sequences encoding bovine Ric8A1–492, Ric8A1–452, and Ric8A1–426 were amplified from a Ric8A cDNA clone obtained from Dharmacon (accession number NM_001015627.2) and cloned into the NdeI-XhoI sites of the pET15b vector (Novagen). Ric8A1–492 mutations were introduced using the QuikChange protocol for site-directed mutagenesis. For Small Angle X-ray Scattering (SAXS) experiments, Ric8A1–492 and Ric8A1–452 were cloned into a modified pRSF Duet vector (Novagen), with a 34 amino-acid linker introduced between the 6xHis-tag and TEV protease cleavage site, and a 3 amino-acid linker introduced between the TEV protease cleavage site and Ric8A. Sequences coding for Gα_s_, Gα_i_, and chimeric Gα_t_^46,47^ were cloned into the NcoI-XhoI sites of a modified pET21a vector containing an N-terminal 6xHis-tag and a TEV protease cleavage site. For the cloning of minimal Gα_i_ (miniG_i_), the 8 N-terminal residues of Gα_i1_ were deleted, a helical domain corresponding to residues 61–177 was replaced with the Glu-Glu epitope tag (EYMPME) linker, residues 231–237 from the switch III region were deleted, and six amino-acid substitutions (G41D/E41N/G219D/T221A/A228D/P290Q) were introduced using overlap extension PCR^39,48,49^. This miniG_i_ was cloned into the NcoI-XhoI sites of a modified pET21a vector. All PCR primers used in this study are listed in Supplementary Table 3.

For crystallography, we generated a surface entropy reduction mutant of Ric8A1–492, ^460^EEK^462^→AAA^50^. For crystallography and biochemical studies, we generated a fusion protein of Gα_t_340–350 with the B1 domain of Streptococcal protein G (GB1-Gα_t_340–350) and a fusion protein of Gα_t_327–350 with maltose-binding protein (MBP). Sequence Gα_t_340–350 was chosen because the corresponding Gα_t_ peptide potently binds to activated rhodopsin, and the 11 C-terminal residues of Gα_i/t_ form the most important interface with rhodopsin^40,51^. The sequence coding for Gα_t_340–350 was cloned into BamHI-HindIII sites of a modified pQE30 vector containing the GB1 tag. A longer sequence Gα_t_327–350 was used in the MBP-fusion protein to allow for a linker, such that potential crystal packing interactions would not interfere with the known interaction involving the C-terminal 18-mer peptide of Gα^26^. MBP was modified by introducing multiple mutations that improve its ability to crystalize^52,53^. *E. coli* MBP28–394 with amino-acid substitutions D110A/K111A/E200A/N201A/A243H/K247H/K267A was attached to Gα_t_327–350 via an AAAH linker using overlap extension PCR. The construct was cloned into the NdeI-XhoI sites of the pET15b vector.

All constructs were transformed into *E. coli* strain BL21(DE3) (Novagen). Cells expressing Ric8A1–492 and Ric8A1–452 were grown to OD_600_ = 0.6 in Terrific Broth (TB) medium at 37 °C and induced with 0.5 mM IPTG at 22 °C overnight. Cells expressing Ric8A1–426 were grown to OD_600_ = 0.6 in TB medium at 37 °C and induced with 0.5 mM IPTG at 22 °C for 2 h. For the expression of all full-length Gα constructs, cells were grown to OD_600_ = 0.6 in 2TY medium at 37 °C and induced with 50 μM IPTG at 17 °C overnight. Cells expressing miniGα_i_ were grown to OD_600_ = 0.6 in LB medium supplemented with 2 mM MgSO4 at 37 °C and induced with 50 μM IPTG at 17 °C overnight. Cells expressing MBP-Gα_t_327–350 were grown to OD_600_ = 0.6 in LB medium at 37 °C to OD_600_ = 0.6 and induced with 0.5 mM IPTG at 37 °C overnight.

Cells expressing 6xHis-tagged Ric8A1–492, Ric8A1–452, Ric8A1–426, Gα_t_, Gα_s_, Gα_i,_ or MBP-Gα_t_327–350 were resuspended in buffer N1 (50 mM HEPES, 300 mM NaCl, 5% glycerol, pH 8.0) supplemented with a Complete^TM^, Mini, EDTA-free Protease Inhibitor Cocktail tablet (Roche) and 2 mM PMSF. For Gα constructs, cell suspensions were also supplemented with 10 mM MgCl_2_ and 50 μM GDP. Cells were lysed by sonication, cell debris was cleared by centrifugation and supernatant was loaded onto His-bind resin (EMD Millipore) charged with Ni^++^. Resin was washed with 5-column volumes of resuspension buffer followed by buffer N1 containing 30 mM imidazole. Proteins were eluted with buffer N1 containing 300 mM imidazole. Ric8A1–492 and Ric8A1–452 were dialyzed against 20 mM phosphate buffer (pH 7.0) containing 5% glycerol, 5 mM β-mercaptoethanol (buffer S1). They were then further purified by SP-sepharose (GE healthcare) cation exchange chromatography. Resin was washed first with buffer S1 and then with buffer S1 containing 25 mM NaCl. Ric8A1–492 and Ric8A1–452 were eluted using buffer S1 containing 250 mM NaCl. Due to its poor solubility and/or stability at pH 7.0, Ric8A1–126 was purified using HiTrapQ anion exchange chromatography, as were Gα_t_, Gα_s_, and Gα_i_. After purification using the His•Bind resin, these proteins were dialyzed against 20 mM HEPES (pH 8.0) buffer containing 50 mM NaCl, 5% glycerol, 1 mM tris(2-carboxyethyl)phosphine (TCEP) (buffer Q1) and loaded onto a HiTrapQ column (GE healthcare). Proteins were eluted using a linear 0.05–1 M NaCl gradient in buffer Q1. Where the His6-tag was not removed, Ric8A1–492, Ric8A1–452, Ric8A1–426, Gα_t_, Gα_s_, or Gα_i,_ was further purified by size-exclusion chromatography (SEC) using a HiLoad 16/600 Superdex 200 pg column equilibrated with 20 mM Tris, 150 mM KCl, 5% glycerol, 1 mM TCEP, pH 7.5. His tags were removed from Ric8A constructs by adding TEV protease at a 1:10 molar ratio, followed by an overnight incubation at 4 °C. Samples were then passed through His•Bind resin to remove uncleaved proteins and purified by SEC as described above.

The miniGα_i_ construct was expressed and purified according to the procedures described above with the following modifications. Buffer N2 (50 mM HEPES, 150 mM NaCl, 20 mM MgSO4, 10% glycerol, pH 8.0) supplemented with 50 μM GDP was used to resuspend and sonicate the cells. His•Bind resin was charged with Co^++^. After the column was loaded with the supernatant, it was washed with buffer N2 containing 10 mM imidazole and the protein was eluted with buffer N2 containing 150 mM imidazole and 50 µM GDP. The sample was then incubated overnight with TEV protease at a 1:50 molar ratio at 4 °C and purified by SEC using a HiLoad 16/600 Superdex 75 pg column equilibrated with 20 mM Tris-HCl (pH 8.0) buffer containing 150 mM KCl, 10% glycerol, 20 mM MgSO4, 10 µM GDP, and 1 mM TCEP.

Ric8A1–492/miniGα_i_ complex was prepared by mixing Ric8A with miniGα_i_ at a 1:1.5 molar ratio. The complex was purified by SEC using a HiLoad 16/600 Superdex 200 pg column equilibrated with 20 mM Tris-HCl (pH 8.0) buffer containing 150 mM KCl, 5% glycerol, and 1 mM TCEP. This procedure removed excess miniGα_i_, ensuring 1:1 stoichiometry of the complex. Ric8A1–492/Gα_t_ complexes were purified by mixing Ric8A1–492 with Gα_t_ (1:1.5 molar ratio), followed by SEC using Superdex 200 10/300 GL column (GE healthcare) to remove excess Gα_t_.

MBP-Gα_t_327–350 was purified by Ni-NTA chromatography followed by SEC using a HiLoad 16/600 Superdex 200 pg column equilibrated with 20 mM Tris-HCl (pH 7.5) buffer containing 100 mM NaCl, 1 mM EDTA, and 2 mM TCEP. Ric8A1–492/MBP-Gα_t_327–350 complexes were prepared by mixing the proteins at a 1:1.5 molar ratio in 5 mM maltose and loading the mixture onto a HiLoad 16/600 Superdex 200 pg column equilibrated with 20 mM Tris-HCl (pH 7.5) buffer containing 150 mM KCl, 5% glycerol, and 1 mM TCEP.

### Crystallization, data collection, and structure determination

MBP-Gα_t_327–350 was crystalized using the hanging drop vapor-diffusion method. Specifically, 0.3 µl of protein (60 mg/ml, 5 mM maltose) was mixed with 0.3 µl of crystallization solution containing 2 M ammonium sulfate, 0.1 M sodium acetate (pH 4.6) at 18 °C, using a TTP LabTech Mosquito crystallization robot (TTP labtech). Apo Ric8A1–492 was also crystallized using the hanging drop vapor-diffusion method. In this case 0.3 µl of Ric8A1–492 at 35 mg/ml was mixed with 0.3 µl of 0.1 M bis-tris propane (pH 6–7), 0.2 M NaI, 12–20% PEG3350 at 4 °C using a TTP LabTech Mosquito crystallization robot. Crystals obtained were used to seed 1:1 μl drop in a sitting drop vapor-diffusion set-up. Crystals were cryo protected using 20% sucrose in mother liquor. The Ric8A1–492/MBP-Gα_t_327–350 complex was crystallized by mixing 0.3 µl at 40 mg/ml with 0.3 µl of 0.1 M MIB buffer, 25% PEG3350, pH 8.0 at 18 °C, using a TTP LabTech Mosquito crystallization robot.

The data sets were collected at the Advanced Light Source Beamline 4.2.2 (Berkley, CA). MBP-Gα_t_327–350 crystals were exposed to the beam for 0.2 s with a wavelength of 1 Å at 0.2° oscillation per frame, and data were collected across a 180° rotation. For apo Ric8A1–492, crystals were exposed to the beam for 1 second, with 0.2° of oscillation per frame, and two data sets were collected across a 180° rotation. For crystals of the Ric8A1–492/MBP-Gα_t_327–350 complex, two data sets were collected by exposing crystals to the beam for 0.1 s, with 0.1° of oscillation across a 180° rotation. The data sets were indexed and integrated using X-ray detector software XDS^54^ and scaled using the Scala software^55^. For apo Ric8A and the Ric8A1–492/MBP-Gα_t_327–350 complex, the two data sets were merged and the structures were solved by molecular replacement. To solve the structure of MBP-Gα_t_327–350, PDB ID 1ANF was used as search model and molecular replacement was done using Phaser crystallographic software^56^. The structure of the Ric8A1–492/MBP-Gα_t_327–350 complex was solved by molecular replacement using the MBP-Gα_t_327–350 structure as a search model. The structure of apo Ric8A1–492 was solved using the Ric8A structure from the complex as a search model. The structures were refined using PHENIX^57^ and Coot. For MBP-Gα_t_327–350, Ramachandran favored, allowed and outliers (%) were 99.23, 0.77, and 0. For Ric8A1–492/MBP-Gα_t_327–350, Ramachandran favored, allowed and outliers (%) were 97.46, 2.42, and 0.13. For apoRic8A, Ramachandran favored, allowed and outliers (%) were 97.44, 2.05, and 0.51. Figures were generated using Pymol. Electrostatic surfaces were calculated using the APBS software^58^.

### Protein thermostability assays

Stabilities of Ric8A constructs with and without peptide ligands were assessed using differential scanning fluorimetry (DSF), in which an increase in fluorescence of the Sypro Orange dye is measured. Ric8A1–492, Ric8A1–452 and Ric8A1–426 in 20 mM Tris-HCl (pH 7.5) buffer containing 150 mM KCl, 5% glycerol, and 1 mM TCEP with and without peptide were used at a final concentration of 0.5 mg/ml and were supplemented with a 1000-fold dilution of Sypro Orange dye (Invitrogen). Temperature was increased at 1 °C/minute and fluorescence signals were recorded using real-time PCR (C1000 Touch thermal cycler, Bio Rad), with the cycler set to FRET mode and the excitation wavelength set at 450–490 nm and the emission wavelength at 560–580 nm.

### Bio-layer interferometry binding assay

An Octet RED96 system and streptavidin (SA)-coated biosensors (FortéBio, Menlo Park, CA) were used to measure association and dissociation kinetics for Gα peptides or the full-length Avi-tagged Gα_t_ in relation to Ric8A1–492, Ric8A1–452, and Ric8A1–426. To obtain the Avi-tagged Gα_t_, Gα_t_ was cloned into modified pET21a vector with the N-terminal His6-tag followed by the Avi tag and TEV cleavage site^59^. The Avi-tagged I340Q/N343H mutant of Gα_t_ was prepared by QuikChange protocol. BL21(DE3) cells for expression of the Avi-tagged Gα_t_ were grown in 2TY media supplemented with biotin (10 mg/liter), induced at OD_600_ of 0.6 with 50 µM IPTG and further grown overnight at 16 °C. Binding studies were performed in 20 mM Tris, 150 mM KCl, 5% glycerol, 1 mM TCEP, 0.5 mg/ml BSA, pH 8.0. All steps were performed at 26 °C, with biosensors stirred into 0.2 ml of sample in each well at 1000 rpm, and at a data acquisition rate of 5.0 Hz. N-terminally biotinylated Gα_t_333–350, Gα_s_363–380, C1 peptide and C2 peptide were loaded onto SA sensors at a concentration of 0.05, 0.05, 0.005, and 0.01 mg/ml for 40–90 seconds. Data for association and dissociation phases of the assay were collected as shown in Fig. 1b and Supplementary Figs. 8, 11, 12, and 14. To correct for baseline drift and non-specific binding, reference sensors lacking bound Gα peptide were used in the BLI assays with Ric8A proteins at the highest concentrations. Kinetic data fitting was performed using FortéBio Data Analysis software 10.0. For each concentration of Ric8A1–492, dissociation rate constant (*k*_d_) values were calculated from the corresponding dissociation phases of the curves. These *k*_d_ values were used to calculate the association rate constant (*k*_a_) values from the association phases for each concentration according to the equation

1 $$k_{\mathrm{a}} = \left( {k_{{\mathrm{observed}}} - k_{\mathrm{d}}} \right)/\left[ {{\mathrm{Ric}}8A1-492} \right]$$

The average *k*_a_ and *k*_d_ were calculated as means of the individual *k*_a_ and *k*_d_ values for all curves. Equilibrium dissociation constant *K*_D_ was calculated as mean *k*_d_/mean *k*_a_. Steady-state data fitting was performed using the GraphPad Prism 7 software with the equation for one site specific binding.

### Crosslinking

Apo Ric8A1–492, and complexes of Ric8A1–492 with Gα_t_ and miniGα_i_, were purified by SEC on a column equilibrated with 20 mM HEPES (pH 8.0) buffer containing 150 mM KCl, 5% glycerol and 1 mM TCEP. Crosslinking reactions were initiated by adding disuccinimidyl suberate (DSS) (0.5 mM final concentration) to apo Ric8A1–492 (0.3 mg/ml), Ric8A1–492/Gα_t_ (0.3 mg/ml) or Ric8A1–492/miniGα_i_ (0.35 mg/ml) at 25 °C. Forty minutes after being initiated, they were quenched by adding Tris-HCl pH 7.5 to a final concentration of 30 mM. Crosslinked proteins were resolved by SDS-PAGE.

### PAGE and in-gel trypsin digestion

An estimated 3 µg of crosslinked protein was loaded onto NuPage 4–12% Bis-Tris precast gels (Invitrogen, USA) and separated at 150 V for 1.5 h. Sharp Pre-stained Protein Standards (10 µl, were loaded onto a separate gel lane to serve as a guide to molecular weight. The gel was stained using a Pierce Silver Stain Kit (Thermo Scientific, USA) following the manufacturer’s directions.

A procedure slightly modified from the one described previously was used for in-gel digestion^60^. In brief, the targeted protein bands from the SDS-PAGE gel were manually excised, cut into 1 mm^3^ pieces, and washed in 100 mM ammonium bicarbonate:acetonitrile (1:1, v/v) and then in 25 mM ammonium bicarbonate:acetonitrile (1:1, v/v) to achieve complete destaining.

The gel pieces were further treated with acetonitrile, to effectively dry them and then reduced in 50 μl of 10 mM DTT at 56 °C for 60 min. The gel-trapped proteins were then alkylated with 55 mM chloroacetamide (CAM) for 30 min at room temperature. The gel pieces were washed twice with 25 mM ammonium bicarbonate:acetonitrile (1:1, v/v) to remove excess DTT and CAM, after which 50 μl of cold trypsin solution at 10 ng/μl in 25 mM ammonium bicarbonate was added to the gel pieces and they were allowed to swell on ice for 60 min. Digestion was conducted at 37 °C for 16 h. Peptides were extracted by adding 100 μl of 50% acetonitrile/0.1% formic acid for 0.5 h three times and combining the supernatants. The combined extracts were concentrated using a lyophilizer and rehydrated in 15 μl of Mobile Phase A solution.

### LC-MS/MS

Mass spectrometry data were collected using an Orbitrap Fusion Lumos mass spectrometer or an Q-Exactive HF Orbitrap mass spectrometer (Thermo Fisher Scientific, San Jose, CA) coupled to an Easy-nLC-1200™ System (Proxeon P/N LC1400). The autosampler was set to aspirate 3 μl (estimated 0.2 ug) of reconstituted digest and load the solution on a 2.5-cm C18 trap (New Objective, P/N IT100–25H002) coupled to waste, HV or analytical column through a microcross assembly (IDEX, P/N UH-752). Peptides were desalted on the trap using 16 μl mobile phase A in 4 min. The waste valve was then blocked and a gradient was run at a 0.4 μl/min flow rate through a self-packed analytical column (10 cm in length×75 µm inner diameter). The fused silica column was tapered from 75 µm ID (Polymicro) to ~8 µm at the tip using a Sutter P-2000 laser puller, and then packed with 2.7 μm Halo C18 particles using a He-pressurized SS cylinder. Peptides were separated in-line with the mass spectrometer using a 70-min gradient composed of linear and static segments wherein buffer A is 0.1% formic acid and buffer B is 95% acetonitrile, 0.1% formic acid. The gradient first holds at 4% for 3 min then makes the following transitions (%B, min): (2, 0), (35, 46), (60, 56), (98, 62), (98, 70).

### Tandem mass spectrometry using Orbitrap Fusion Lumos

Data acquisition was initiated with a survey scan (*m*/*z* 380–1800) acquired on an Orbitrap Fusion Lumos mass spectrometer at a resolution of 120,000 in the off axis Orbitrap segment (MS1), with Automatic Gain Control (AGC) set to 3E06 and a maximum injection time of 50 ms. MS1 scans were acquired every 3 s during the 70-min gradient described above. The most abundant precursors were selected from among 2–6 charge state ions at a 1E05 Automatic Gain Control (AGC) and 70 ms maximum injection time. Ions were isolated with a 1.6-Th window using the multi-segment quadrupole and subjected to dynamic exclusion for 30 sec if they were targeted twice during the prior 30-s period. The selected ions were then sequentially subjected to collision-induced dissociation (CID) and high energy collision-induced dissociation (HCD) activation in the IT and the ion routing multipole respectively (IRM). The AGC target for CID was 4.0E04, 35% collision energy, with an activation Q of 0.25 and a 75 ms maximum fill time. Targeted precursors were also fragmented by high energy collision-induced dissociation (HCD) at 30% collision energy in the IRM. HCD fragment ions were analyzed using the Orbitrap (AGC 1.2E05, maximum injection time 110 ms, and resolution set to 30,000 at 400 Th). Both MS2 channels were recorded as centroid and the MS1 survey scans were recorded in profile mode.

### Tandem mass spectrometry using Orbitrap Q-Exactive HF

Data dependent acquisitions (DDA) began with a survey scan (m/z 380–1800) acquired on a Q-Exactive HF Orbitrap mass spectrometer at a resolution of 120,000 in the off axis Orbitrap segment (MS1) with AGC set to 3E06 and a maximum injection time of 50 ms. MS1 scans were acquired every 3 s during the 70 min gradient described above. The most abundant precursors were selected among 2–5 charge state ions observed in MS1 and isolated with a 1.6-Th window using the segment quadrupole. Selected ions were subjected to HCD using 1E05 AGC and 70 ms injection time thresholds. Ions were subject to dynamic exclusion for 30 s if they were targeted twice in the ion routing multipole (IRM) during the prior 30 sec. Targeted precursors were fragmented by HCD at 30% collision energy and fragment ions were analyzed using the Orbitrap (AGC 1.2E05, maximum injection time 110 ms, and resolution set to 30,000 at 400 Th). MS1 survey scans were recorded in profile mode and MS2 data were recorded as centroid.

### Identification of crosslinked peptides

Peak lists in the form of mgf files were submitted for the search using Protein Prospector Batch-Tag Web^34,61^. The database searched contained the sequences of Ric8A1–492, Gα_t_ or miniGα_i_. In addition, the target sequences were randomized 10 times and appended to the target sequences. Eighty peaks from each spectrum were searched using a precursor charge range of 2–5, a tolerance of 20 ppm for precursor ions and 1 Da for fragment ions, and an instrument setting of ESI-Q-hi-res. Cleavage selectivity was set to that of trypsin, and up to three missed cleavages per peptide were allowed. Carbamidomethylation of cysteines was specified as a constant modification. Protein Prospector Search Compare program was used to generate a Crosslinked Peptides report. The score of a crosslinked peptide was based on number and types of fragment ions identified, as well as the sequence and charge state of the crosslinked peptide. Only results where the score difference is >0 (i.e., the crosslinked peptide match was better than a single peptide match alone) are considered^61^. The expectation value represents how many random matches would be expected to achieve a given score or greater, in a search of a given size. The expectation values are calculated based on matches to single peptides and thus should be treated as another score, rather than a statistical measure of reliability^61^. There were no matches to decoy sequences in the search of intramolecular crosslinks of Ric8A1–492, suggesting a false discovery rate (FDR) of <1%. FDRs of ~4.5% and 1.5% were estimated in the Ric8A1–492/miniGα_i_ and Ric8A1–492/Gα_t_ crosslinking searches, respectively, as described previously^61^. Low-scoring intermolecular crosslink matches were filtered according to an FDR of 5%.

### GTPγS binding to Gα_i_

The rates of GTPγS binding to Gα_i_ in the absence or presence of Ric8A1–492 (Ric8A1–452) were measured by following the increase in fluorescence of Gα_i_ tryptophan. Gα_i_ (1 µM) was mixed with GDP (1 µM) in a fluorescence cuvette, in 20 mM Tris-HCl (pH 8.0) buffer containing 150 mM NaCl, 5% glycerol, 1 mM TCEP and 10 mM MgCl_2_, and incubated for 2 min. The binding reaction was initiated by addition of Ric8A1–492 or Ric8A1–452 (1 µM each) and/or and GTPγs (10 µM). Fluorescence at 340 nm was monitored with the excitation set at 295 nm. Data were fit to an equation for one phase association using the GraphPad Prism 7.05 software.

### Small angle X-ray scattering

SAXS data were collected at the Bio-CAT beamline 18-ID-D at the Advanced Photon Source (APS; Argonne, IL) using an in-line size-exclusion chromatography SAXS (SEC-SAXS) configuration^62^ with superdex 200 column (GE Healthcare). A 250-µl volume of 10 mg/ml sample in 20 mM Tris, 150 mM KCl, 5% glycerol, 1 mM TCEP, pH 7.5 buffer was loaded onto the column at flow rate at 0.9 ml/min. The elution trajectory was redirected into the SAXS sample flow cell (1.5 mm ID quartz capillary with 10 µm walls) after the UV monitor. Scattering data were collected every 2 s using a 0.5-s exposure on a Pilatus 3 × 1 M pixel detector (DECTRIS) covering a *q*-range of 0.0040 < *q* < 0.388 Å^−1^ (*q* = 4*π*/*λ* sin *θ*, where 2*θ* is the scattering angle). For each protein, the buffer scattering before and after the eluted peak was recorded and used for background correction. The final protein scattering curves were obtained by scaling the data from the main peak, and averaging it and correcting for buffer scattering. BioXTAS RAW and ATSAS 2.8 were used for SAXS data reduction and analysis^63,64^.

### Molecular dynamics simulations

MD simulations were performed using YASARA Structure 18.2.7 and the md_runfast macro. For simulation of Ric8A1–426, the Ric8A1–492 structure solved in the P2_1_ space group (PDB 6N85) was used as starting model. Missing residues from the loop regions were modeled using the YASARA Structure 18.2.7 before starting the simulation. For the simulation of Ric8A1–452, the top selected FloppyTail model was used. For simulation of miniGα_i_, a homology model of miniGα_i_ lacking the 25 N-terminal residues (ΔN25-miniGα_i_) was built based on the template structure of miniGα_s_ in complex with the β2 adrenergic receptor (PDB ID 5G53) using the YASARA program. This choice of template produced fewer clashes in modeling the complex of miniGα_i_ with Ric8A compared to the use of miniGα_o_ structure (PDB: 6FUF). The simulations were run using the AMBER14 force field in water at a temperature of 298 K or 310 K, pH of 7.4 and NaCl concentration of 0.9%. The particle mesh Ewald summation was used to compute long-range coulombic interactions with a periodic cell boundary and a cutoff of 8 Å. The MD simulations were analyzed using the md_analyze macro in YASARA and Pymol programs. All of the parameters of these MD simulations except for RMSF were calculated using YASARA. RMSF (Cα) values were calculated using VMD. Backbone atoms of residues corresponding to 80–280 of Ric8A was aligned before the RMSF calculations were performed using built in RMSF calculation function in VMD^65^.

### Steered molecular dynamics simulations

SMD was performed on a conformation of the Ric8A1–452 model that was derived in MD simulations and showed minimal clashes on modeling of the Ric8A1–452/ ΔN25-miniGα_i_ complex. The structure file was prepared using VMD^65^ and the plugin QwikMD^66^. The MD simulations were performed employing the NAMD molecular dynamics package^67^ and the CHARMM36 force field^68^. The Minimization and Constrained equilibration MD Simulation was performed with implicit solvent represented by the Generalized Born/solvent-accessible surface area model^69,70^. A temperature ramp was performed and consisted of 0.24 ns of simulation where the temperature was raised from 60 K to 300.00 K. Before the SMD simulations all the systems were submitted to an energy minimization protocol for 1000 steps. In this step consisted of 1.00 ns of simulation, the atoms defined by the selection “protein and backbone” were restrained. The SMD simulation was performed with implicit solvent represented by the Generalized Born/solvent-accessible surface area model^69,70^. The temperature was maintained at 300.00 K using Langevin dynamics. A distance cutoff of 16.0 Å was applied to short-range, non-bonded interactions, and 15.0 Å for the smothering functions. The equations of motion were integrated using the r-RESPA multiple time step scheme^67^ to update the short-range interactions every 1 steps and long-range electrostatics interactions every 2 steps. The time step of integration was chosen to be 2 fs for all simulations. The SMD simulations^71^ of constant velocity stretching (SMD-CV protocol) employing a pulling speed of 2.5 Å/ns and a harmonic constraint force of 7.0 kcal/mol/Å^2^ was performed for 4.0 ns. In this step, SMD was employed by harmonically restraining the position of Ric8A residues 1–13 and moving a second restraint residues 296–452 with constant velocity in the axis defined by the center of mass of the ΔN25-miniGα_i_ atoms that clash with Ric8A and the center of mass of the Ric8A atoms that clash with ΔN25-miniGα_i_. Residues 296–452 were selected as moving because in MD simulations of Ric8A1–452 they behaved as a module that fluctuated relatively independent of the rest of the molecule. It is this module that was clashing with ΔN25-miniGα_i_.

### Modeling of the proximal C terminal tail of Ric8A

The crystal structure of Ric8a1–426 was used as the starting model in modeling of the proximal portion of the C-terminal tail. Given that the regular secondary structure of the core domain ends at residue 422, the starting model was generated based on the structure of Ric8A1–422 with Ric8A423–452 attached in a random conformation that extended away from the core of the molecule. The structures of the missing loops were modeled using the YASARA program, and the resulting structure was used in further modeling with the FloppyTail application^35^ of the Rosetta software suite. The FloppyTail algorithm generates hypothetical, low-energy conformations for disordered or flexible regions using two-stage modeling. The first stage is the centroid phase, during which side-chains are represented by a single, large centroid atom and is designed to collapse long tails into a reasonable conformation. The second stage involves restoration of the side-chains, fine sampling of the backbone conformational space, side-chain optimization, and minimization^35,72^. The various options for the FloppyTail algorithm were set using a flag file (provided as Supplementary Note 1). The flag file was modified from the original flag file provided by Steven Lewis as part of the Rosetta software suite. Two experimental distance constraints based on the highest-scoring C-terminal crosslinks K408/K449 and K352/K449 identified by XL-MS were used during the FloppyTail calculation with flat harmonic function (eq. 2)^73^.

2 $$f\left( {{\mathrm{dist}}} \right) = \left\{ {\begin{array}{*{20}{l}} 0 \hfill & {{\mathrm{if}}\;{\mathrm{dist}} \le {\mathrm{tolerance}}\; \pm \;x_0} \hfill \\ {\left( {\frac{{{\mathrm{dist}} - x_0 - {\mathrm{tolerance}}}}{\sigma }} \right)^2} \hfill & {{\mathrm{otherwise}}} \hfill \end{array}} \right.$$

The three parameters chosen for the calculation were *x*_0_ = 15 Å, tolerance = 15 Å and $\sigma$ = 1. Effectively, these parameters limit the distance between Cα atoms to < 30 Å. The length of the DSS crosslinker (11.4 Å) combined with the length of 2 Lys side-chains (6.4 + 6.4 Å) yields a maximal distance of ~24 Å between the Cα–Cα atoms. Considering protein dynamics (flexibility) this distance is adjusted to a threshold of <30 Å^74^. Simulations were performed using 56 cores on the Argon cluster at the University of Iowa. After the FloppyTail simulation, Rosetta score_jd2 executable was used to calculate energy scores of the models, and the Crysol program^75^ was used to generate and compare fits of theoretical SAXS profiles of the models to experimental SAXS data (*χ*^2^ values). Energy scores from Rosetta and *χ*^2^ were used to select the models. The range of *χ*^2^ values among the 500 top energy models was 1.13–5.00. As a first step in model selection, 311 models were picked from the top 500 energy score models using the cutoff *χ*^2^<2.0. Next, the pool of models was further narrowed to 212 using the crosslinking distance constraint of <30 Å^74^ for the Cα atoms of the third highest scoring C-terminal crosslinked pair K375/K449; this constraint was not used in the FloppyTail modeling. Clustering of 212 models was performed with the Ensemble Cluster tool from UCSF Chimera software^36^, and it yielded two main clusters I and II that were comprised of 50 and 20 models, respectively. The remaining clusters were minor each containing 13 or fewer models. None of the minor clusters included models from the top 10 energy score models. Thus, these clusters totaling 142 models were excluded from further analysis.

### Modeling of apo Ric8A1–492

The starting model for apo Ric8A1–492 was built using the YASARA application and the model of Ric8A1–452, which served as a template, and was subjected to a conformational sampling analysis using the BILBOMD server^37^. Residues Ric8A1–452 and an α-helix Ric8A 471–490 (Supplementary Fig. 6) were treated as rigid domains linked with a flexible linker Ric8A453–470. Thus, 800 BILBOMD models were generated using MD simulations and validated against the experimental SEC-SAXS profile of Ric8A1–492 (Supplementary Fig. 19).

### Modeling of Ric8A-miniGα_i_ and Ric8A-Gα_t_ complexes

The starting model for the complex was produced by superimposition of the α5 helix of the MD models of ΔN25-miniGα_i_ onto the corresponding helical segment of the C terminal G_t_ peptide in complex with Ric8A, which was aligned with the SMD models of Ric8A1–452. The SMD model of Ric8A1–452 and MD model of ΔN25-miniGα_i_ that produced no clashes on this superimposition were selected, and their coordinates were merged. Next, the N-terminus of ΔN25-miniGα_i_ in the complex was extended by 6 residues (ΔN19-miniGα_i_) using YASARA to include the crosslinked Lys21 residue of miniGα_i_. Also, the C-terminal CGLF residues of ΔN19-miniGα_i_ were modeled as in the Ric8A-bound structure of Gα_t_327–350. The resulting model of the complex was energy minimized using YASARA and used as input for the FloppyTail calculation. The FloppyTail algorithm was used to model the distal C terminus of Ric8A in the Ric8A1–492/ΔN19-miniGα_i_ complex. The model of the Ric8A1–452/ΔN19-miniGα_i_ complex with the Ric8A453–492 tail appended in the extended conformation was used as a starting point for this modeling. Residues 471–490 were kept helical, based on the prediction by PSIPRED (Supplementary Fig. 6). The FloppyTail protocol for the complex was similar to that described for Ric8A1–452. Two experimental intermolecular distance constraints were used during the simulation. 5548 models generated by FloppyTail were clustered into two major clusters I and II by RMSD with a cutoff of 2.5 Å using the clustering algorithm implemented in the visual molecular dynamics (VMD) program. The top cluster I and II models (models 1 and 2, respectively) in terms of energy score were selected for the 19-residue N-terminal extension to generate the models of Ric8A1–492/miniGα_i_ complex.

The structure of rhodopsin-bound G_i_ (PDB 6CMO) was used to model of the Ric8A/Gα_i_ complex. Additionally, the αN-helix and the α5 helix of Gα_i_ from 6CMO were modeled according to the model of the Ric8A1–492/miniGα_i_ complex. The model of the Ric8A1–492/Gα_t_ was obtained by superimposing the Gα_i_ structure onto the model of the Ric8A1–492/miniGα_i_ complex followed by energy minimization using the YASARA program.

### Reporting summary

Further information on research design is available in the Nature Research Reporting Summary linked to this article.

## Supplementary information

Supplementary Information

Peer Review File

Description of Additional Supplementary Files

Supplementary Data 1

Supplementary Data 2

Supplementary Data 3

Supplementary Data 4

Supplementary Data 5

Supplementary Data 6

Supplementary Data 7

Supplementary Data 8

Reporting Summary

Source Data

## Data Availability

The atomic coordinates have been deposited in the Protein Data Bank (https://www.rcsb.org/) with the PDB accession codes 6N84[10.2210/pdb6N84/pdb], 6N85[10.2210/pdb6N85/pdb], 6N86[10.2210/pdb6N86/pdb]. SAXS data for Ric8A1–492 and Ric8A1–452 were deposited in the Small Angle Scattering Biological Data Bank (https://www.sasbdb.org/) with the accession codes SASDF65[https://www.sasbdb.org/data/SASDF65/] and SASDF75[https://www.sasbdb.org/data/SASDF75/], respectively. The coordinates for the models of Ric8A1–452, apo Ric8A1–492, Ric8A1–492/miniGα complex, and Ric8A1–492/Gα complex are provided as Supplementary Data 5, 6, 7, and 8. The source data underlying Figs. 1a–c, 3f–h, 5a, f and Supplementary Figs. 8, 9, 10, 12, 13, 14B, 15 and 23 are provided as a Source Data file. All other data supporting the findings of this study are available from the corresponding author upon reasonable request.

## Code availability

Rosetta Floppy tail scripts used in this study are available in Supplementary Note 1.

## References

[1] Miller KG (1996). A genetic selection for Caenorhabditis elegans synaptic transmission mutants. Proc. Natl Acad. Sci. USA.

[2] Miller KG, Emerson MD, McManus JR, Rand JB (2000). RIC-8 (Synembryn): a novel conserved protein that is required for G(q)alpha signaling in the C. elegans nervous system. Neuron.

[3] Miller KG, Rand JB (2000). A role for RIC-8 (Synembryn) and GOA−1 (G(o)alpha) in regulating a subset of centrosome movements during early embryogenesis in Caenorhabditis elegans. Genetics.

[4] Matsuzaki F (2005). Drosophila G-protein signalling: intricate roles for Ric-8?. Nat. Cell Biol..

[5] Wright SJ, Inchausti R, Eaton CJ, Krystofova S, Borkovich KA (2011). RIC8 is a guanine-nucleotide exchange factor for Galpha subunits that regulates growth and development in Neurospora crassa. Genetics.

[6] Wilkie TM, Kinch L (2005). New roles for Galpha and RGS proteins: communication continues despite pulling sisters apart. Curr. Biol..

[7] Tall GG (2013). Ric-8 regulation of heterotrimeric G proteins. J. Recept. Signal Transduct. Res..

[8] Tall GG, Krumins AM, Gilman AG (2003). Mammalian Ric-8A (synembryn) is a heterotrimeric Galpha protein guanine nucleotide exchange factor. J. Biol. Chem..

[9] Nagai Y, Nishimura A, Tago K, Mizuno N, Itoh H (2010). Ric-8B stabilizes the alpha subunit of stimulatory G protein by inhibiting its ubiquitination. J. Biol. Chem..

[10] Von Dannecker LE, Mercadante AF, Malnic B (2005). Ric-8B, an olfactory putative GTP exchange factor, amplifies signal transduction through the olfactory-specific G-protein Galphaolf. J. Neurosci..

[11] Klattenhoff C (2003). Human brain synembryn interacts with Gsalpha and Gqalpha and is translocated to the plasma membrane in response to isoproterenol and carbachol. J. Cell Physiol..

[12] Papasergi MM, Patel BR, Tall GG (2015). The G protein alpha chaperone Ric-8 as a potential therapeutic target. Mol. Pharm..

[13] Gabay M (2011). Ric-8 proteins are molecular chaperones that direct nascent G protein alpha subunit membrane association. Sci. Signal.

[14] Chan P (2011). Purification of heterotrimeric G protein alpha subunits by GST-Ric-8 association: primary characterization of purified G alpha(olf). J. Biol. Chem..

[15] Chan P, Thomas CJ, Sprang SR, Tall GG (2013). Molecular chaperoning function of Ric-8 is to fold nascent heterotrimeric G protein alpha subunits. Proc. Natl Acad. Sci. USA.

[16] Rasmussen SG (2011). Crystal structure of the beta2 adrenergic receptor-Gs protein complex. Nature.

[17] Liang YL (2017). Phase-plate cryo-EM structure of a class B GPCR-G-protein complex. Nature.

[18] Zhang Y (2017). Cryo-EM structure of the activated GLP-1 receptor in complex with a G protein. Nature.

[19] Tsai CJ (2018). Crystal structure of rhodopsin in complex with a mini-Go sheds light on the principles of G protein selectivity. Sci. Adv..

[20] Noel JP, Hamm HE, Sigler PB (1993). The 2.2 A crystal structure of transducin-alpha complexed with GTP gamma S. Nature.

[21] Dror RO (2015). SIGNAL TRANSDUCTION. Structural basis for nucleotide exchange in heterotrimeric G proteins. Science.

[22] Figueroa M (2009). Biophysical studies support a predicted superhelical structure with armadillo repeats for Ric-8. Protein Sci..

[23] Lambright DG (1996). The 2.0 A crystal structure of a heterotrimeric G protein. Nature.

[24] Wall MA (1995). The structure of the G protein heterotrimer Gi alpha 1 beta 1 gamma 2. Cell.

[25] Van Eps N, Thomas CJ, Hubbell WL, Sprang SR (2015). The guanine nucleotide exchange factor Ric-8A induces domain separation and Ras domain plasticity in Galphai1. Proc. Natl Acad. Sci. USA.

[26] Thomas CJ (2011). The nucleotide exchange factor Ric-8A is a chaperone for the conformationally dynamic nucleotide-free state of Galphai1. PLoS ONE.

[27] Tewari R, Bailes E, Bunting KA, Coates JC (2010). Armadillo-repeat protein functions: questions for little creatures. Trends Cell Biol..

[28] Huber AH, Nelson WJ, Weis WI (1997). Three-dimensional structure of the armadillo repeat region of beta-catenin. Cell.

[29] Holm L, Laakso LM (2016). Dali server update. Nucleic Acids Res..

[30] Krissinel E, Henrick K (2007). Inference of macromolecular assemblies from crystalline state. J. Mol. Biol..

[31] Flock T (2017). Selectivity determinants of GPCR-G-protein binding. Nature.

[32] Ashkenazy H (2016). ConSurf 2016: an improved methodology to estimate and visualize evolutionary conservation in macromolecules. Nucleic Acids Res..

[33] Kant, R., Zeng, B., Thomas, C. J., Bothner, B. & Sprang, S. R. Ric-8A, a G protein chaperone with nucleotide exchange activity induces long-range secondary structure changes in Galpha. *eLife* **5**, 1–20 (2016). 10.7554/eLife.19238 PMC5182059 28008853

[34] Chalkley RJ, Baker PR, Medzihradszky KF, Lynn AJ, Burlingame AL (2008). In-depth analysis of tandem mass spectrometry data from disparate instrument types. Mol. Cell Proteom..

[35] Kleiger G, Saha A, Lewis S, Kuhlman B, Deshaies RJ (2009). Rapid E2-E3 assembly and disassembly enable processive ubiquitylation of cullin-RING ubiquitin ligase substrates. Cell.

[36] Pettersen EF (2004). UCSF Chimera–a visualization system for exploratory research and analysis. J. Comput. Chem..

[37] Pelikan M, Hura GL, Hammel M (2009). Structure and flexibility within proteins as identified through small angle X-ray scattering. Gen. Physiol. Biophys..

[38] Carpenter B, Nehme R, Warne T, Leslie AG, Tate CG (2016). Structure of the adenosine A(2A) receptor bound to an engineered G protein. Nature.

[39] Nehme R (2017). Mini-G proteins: novel tools for studying GPCRs in their active conformation. PLoS ONE.

[40] Kang Y (2018). Cryo-EM structure of human rhodopsin bound to an inhibitory G protein. Nature.

[41] Shimizu H, Toma-Fukai S, Kontani K, Katada T, Shimizu T (2018). GEF mechanism revealed by the structure of SmgGDS-558 and farnesylated RhoA complex and its implication for a chaperone mechanism. Proc. Natl Acad. Sci. USA.

[42] Graham TA, Ferkey DM, Mao F, Kimelman D, Xu W (2001). Tcf4 can specifically recognize beta-catenin using alternative conformations. Nat. Struct. Biol..

[43] Tarendeau F (2007). Structure and nuclear import function of the C-terminal domain of influenza virus polymerase PB2 subunit. Nat. Struct. Mol. Biol..

[44] Flock T (2015). Universal allosteric mechanism for Galpha activation by GPCRs. Nature.

[45] Papasergi-Scott Makaía M., Stoveken Hannah M., MacConnachie Lauren, Chan Pui-Yee, Gabay Meital, Wong Dorothy, Freeman Robert S., Beg Asim A., Tall Gregory G. (2018). Dual phosphorylation of Ric-8A enhances its ability to mediate G protein α subunit folding and to stimulate guanine nucleotide exchange. Science Signaling.

[46] Natochin M, Gasimov KG, Artemyev NO (2002). A GPR-protein interaction surface of Gi(alpha): implications for the mechanism of GDP-release inhibition. Biochemistry.

[47] Natochin M, Granovsky AE, Artemyev NO (1998). Identification of effector residues on photoreceptor G protein, transducin. J. Biol. Chem..

[48] Carpenter B, Tate CG (2016). Engineering a minimal G protein to facilitate crystallisation of G protein-coupled receptors in their active conformation. Protein Eng. Des. Sel..

[49] Markby DW, Onrust R, Bourne HR (1993). Separate GTP binding and GTPase activating domains of a G alpha subunit. Science.

[50] Goldschmidt L, Cooper DR, Derewenda ZS, Eisenberg D (2007). Toward rational protein crystallization: a Web server for the design of crystallizable protein variants. Protein Sci..

[51] Oldham WM, Hamm HE (2008). Heterotrimeric G protein activation by G-protein-coupled receptors. Nat. Rev. Mol. Cell Biol..

[52] Moon AF, Mueller GA, Zhong X, Pedersen LC (2010). A synergistic approach to protein crystallization: combination of a fixed-arm carrier with surface entropy reduction. Protein Sci..

[53] Laganowsky A (2011). An approach to crystallizing proteins by metal-mediated synthetic symmetrization. Protein Sci..

[54] Kabsch W (2010). Xds. Acta Crystallogr D. Biol. Crystallogr.

[55] Evans P (2006). Scaling and assessment of data quality. Acta Crystallogr. D Biol. Crystallogr..

[56] McCoy AJ (2007). Phaser crystallographic software. J. Appl. Crystallogr..

[57] Adams PD (2010). PHENIX: a comprehensive Python-based system for macromolecular structure solution. Acta Crystallogr. D Biol. Crystallogr..

[58] Baker NA, Sept D, Joseph S, Holst MJ, McCammon JA (2001). Electrostatics of nanosystems: application to microtubules and the ribosome. Proc. Natl Acad. Sci. USA.

[59] Cull MG, Schatz PJ (2000). Biotinylation of proteins in vivo and in vitro using small peptide tags. Methods Enzymol..

[60] Yu CL (2015). Rapid identification and quantitative validation of a caffeine-degrading pathway in Pseudomonas sp. CES. J. Proteome Res..

[61] Trnka MJ, Baker PR, Robinson PJ, Burlingame AL, Chalkley RJ (2014). Matching cross-linked peptide spectra: only as good as the worse identification. Mol. Cell Proteom..

[62] Mathew E, Mirza A, Menhart N (2004). Liquid-chromatography-coupled SAXS for accurate sizing of aggregating proteins. J. Synchrotron Radiat..

[63] Hopkins JB, Gillilan RE, Skou S (2017). BioXTAS RAW: improvements to a free open-source program for small-angle X-ray scattering data reduction and analysis. J. Appl. Crystallogr..

[64] Franke D (2017). ATSAS 2.8: a comprehensive data analysis suite for small-angle scattering from macromolecular solutions. J. Appl. Crystallogr..

[65] Humphrey W, Dalke A, Schulten K (1996). VMD: visual molecular dynamics. J. Mol. Graph.

[66] Ribeiro JV (2016). QwikMD - integrative molecular dynamics toolkit for novices and experts. Sci. Rep..

[67] Phillips JC (2005). Scalable molecular dynamics with NAMD. J. Comput. Chem..

[68] Best RB (2012). Optimization of the additive CHARMM all-atom protein force field targeting improved sampling of the backbone phi, psi and side-chain chi(1) and chi(2) dihedral angles. J. Chem. Theory Comput..

[69] Tanner DE, Phillips JC, Schulten K (2012). GPU/CPU algorithm for generalized born/solvent-accessible surface area implicit solvent calculations. J. Chem. Theory Comput..

[70] Tanner DE, Chan KY, Phillips JC, Schulten K (2011). Parallel generalized born implicit solvent calculations with NAMD. J. Chem. Theory Comput..

[71] Izrailev S, Stepaniants S, Balsera M, Oono Y, Schulten K (1997). Molecular dynamics study of unbinding of the avidin-biotin complex. Biophys. J..

[72] Santiago-Frangos, A., Jeliazkov, J. R., Gray, J. J. & Woodson, S. A. Acidic C-terminal domains autoregulate the RNA chaperone Hfq. *eLife* **6**, 1–25 (2017). 10.7554/eLife.27049 PMC5606850 28826489

[73] Kahraman A (2013). Cross-link guided molecular modeling with ROSETTA. PLoS ONE.

[74] Merkley ED (2014). Distance restraints from crosslinking mass spectrometry: mining a molecular dynamics simulation database to evaluate lysine-lysine distances. Protein Sci..

[75] Svergun D, Barberato C, Koch MHJ (1995). CRYSOL - A program to evaluate x-ray solution scattering of biological macromolecules from atomic coordinates. J. Appl. Crystallogr..

