# Supplementary material for: Structural underpinnings of Ric8A function as a G-protein α-subunit chaperone and guanine-nucleotide exchange factor
Source: Nat Commun. 2019 Jul 12;10:3084. doi: 10.1038/s41467-019-11088-x (PMC6625990; doi:10.1038/s41467-019-11088-x)
Supplement: Supplementary file 1 — Supplementary Information [file 41467_2019_11088_MOESM1_ESM.pdf]

Supplementary Information

Structural underpinnings of Ric8A function as a G-protein α-subunit chaperone and guanine-nucleotide exchange factor

Srivastava et al.

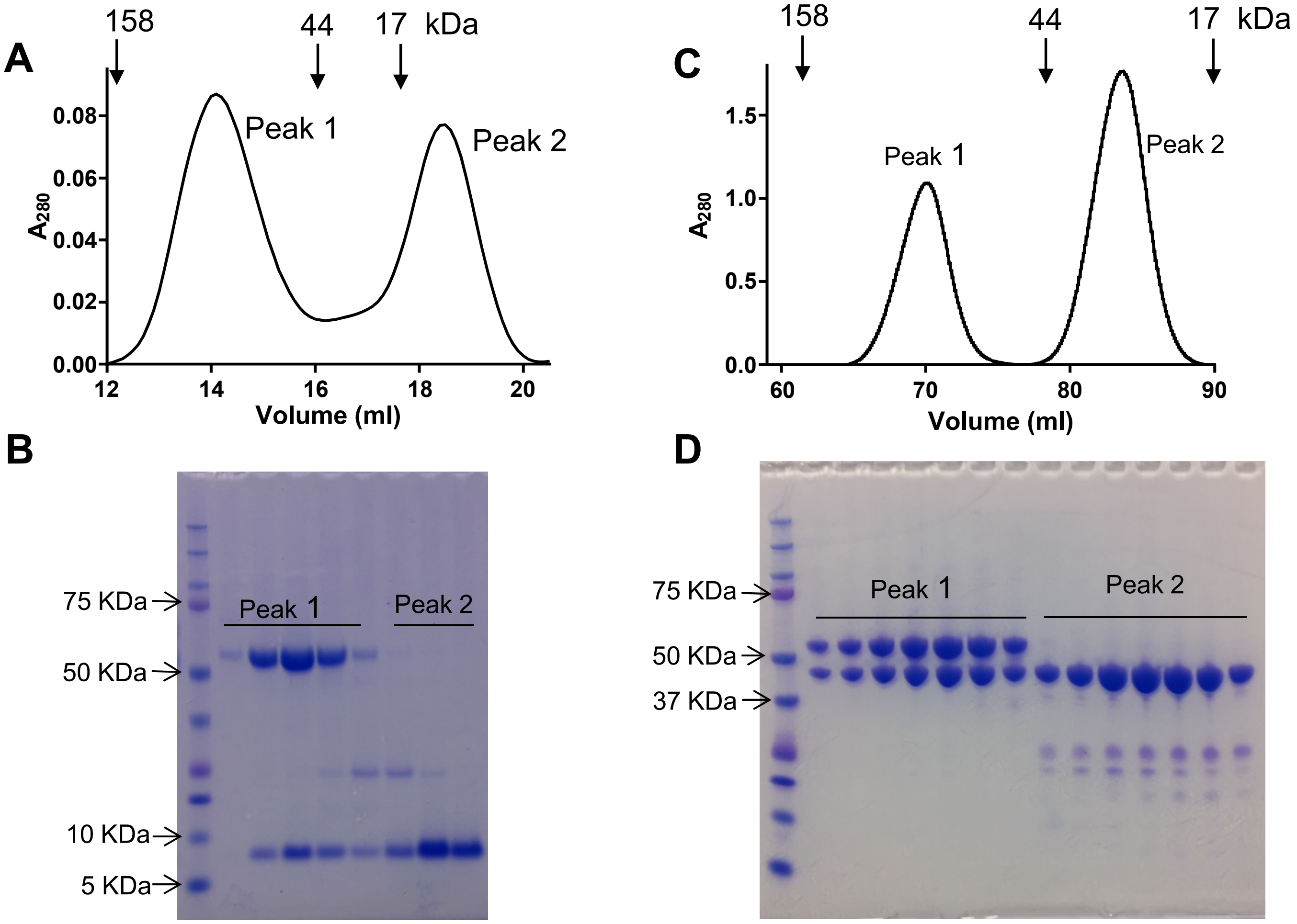

Supplementary Figure 1. Ric8A1-492 forms complexes with GB1-tagged Gα_t340-350 and MBP-tagged Gα_t333-350. (**A**) Ric8A1-492 was mixed with a molar excess of GB1-tagged Gα_t340-350 and loaded onto a MW-calibrated Superose 12 10/300 column. SEC profile (A_{280}). (**B**) Peak fractions in A analyzed by SDS-PAGE. (**C**) Ric8A1-492 was mixed with a molar excess of MBP-tagged Gα_t333-350 and loaded onto a MW-calibrated Superdex 200 16/600 column. SEC profile (A_{280}). (**D**) Peak fractions in C analyzed by SDS-PAGE. First lane in B and D - MW markers.

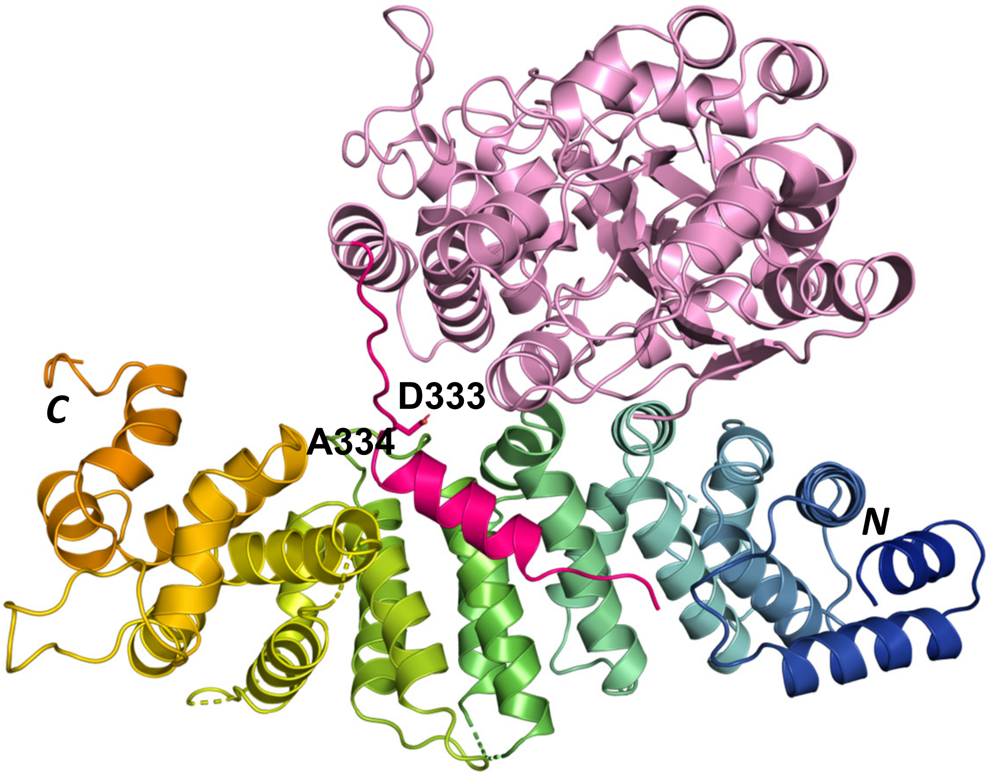

Supplementary Figure 2. Crystal structure of Ric8A1-492 in complex with MBP-tagged Gα_t327-350. Only residues 1-426 of Ric8A can be traced in the electron density map. Ric8A1-426 (rainbow), Gα_t327-350 (magenta), and MBP (pink) are shown in cartoon representation (PDB 6N85). The contacts made by D333 and A334 of Gα_t with Ric8A1-492 might be influenced by the position of MBP in the crystal.

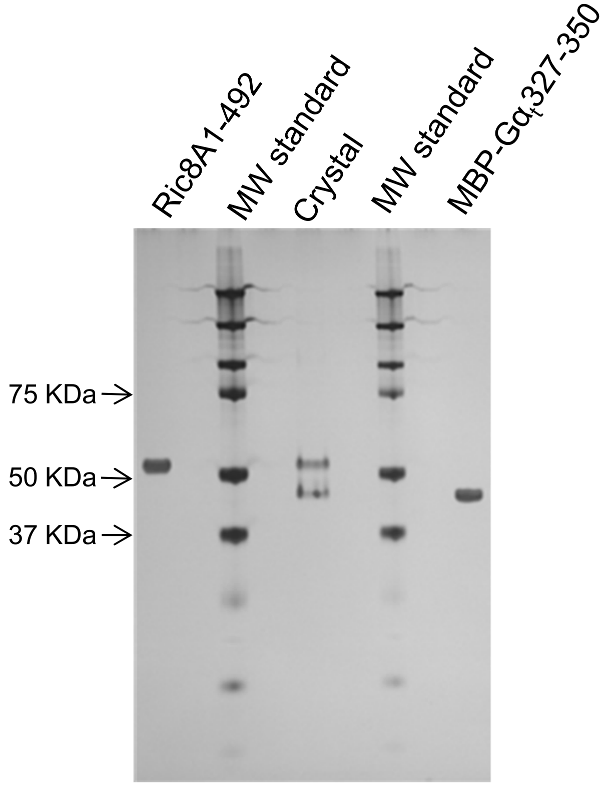

Supplementary Figure 3. Ric8A1-492 in the crystal is unproteolysed. Silver-stained gel. Following the X-ray data collection, the crystal of the Ric8A1-492/MBP-Gα_t327-350 complex was analyzed by SDS-PAGE. Ric8A1-492 and MBP-Gα_t327-350 were loaded for comparison.

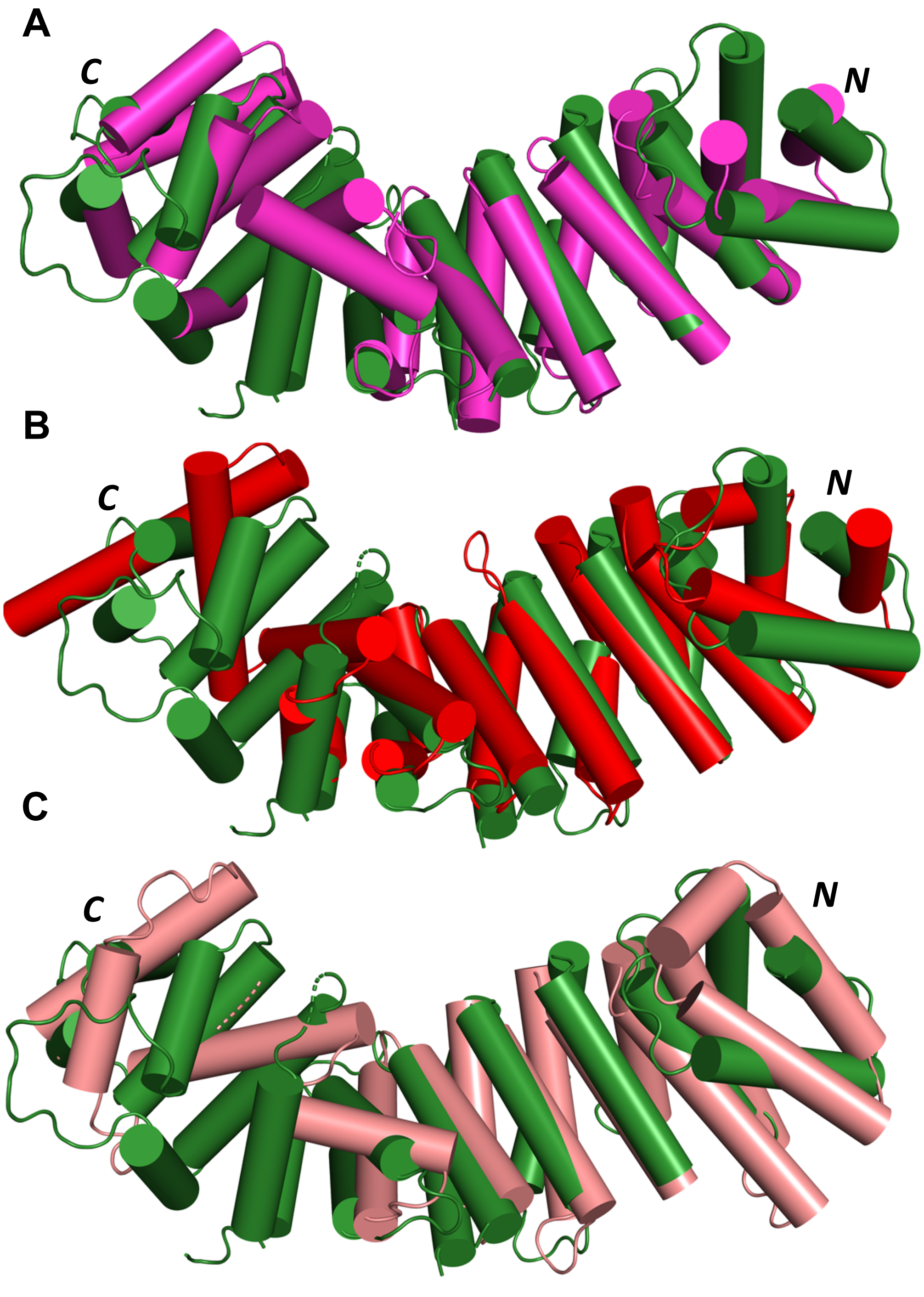

Supplementary Figure 4. Structural homologues of Ric8A. Superposition of the Ric8A armadillo core domain (PDB 6N85) with representative structural homologues β-catenin (PDB 1JDH-A) (**A**), importin-α (PDB 2JDQ-A) (**B**), and SmgGDS (PDB 5XGC-A) (**C**) using DALI server yields RMSD values (Å) of 4.2, 4.1, and 4.2, respectively. Ric8A1-426 is shown in green.

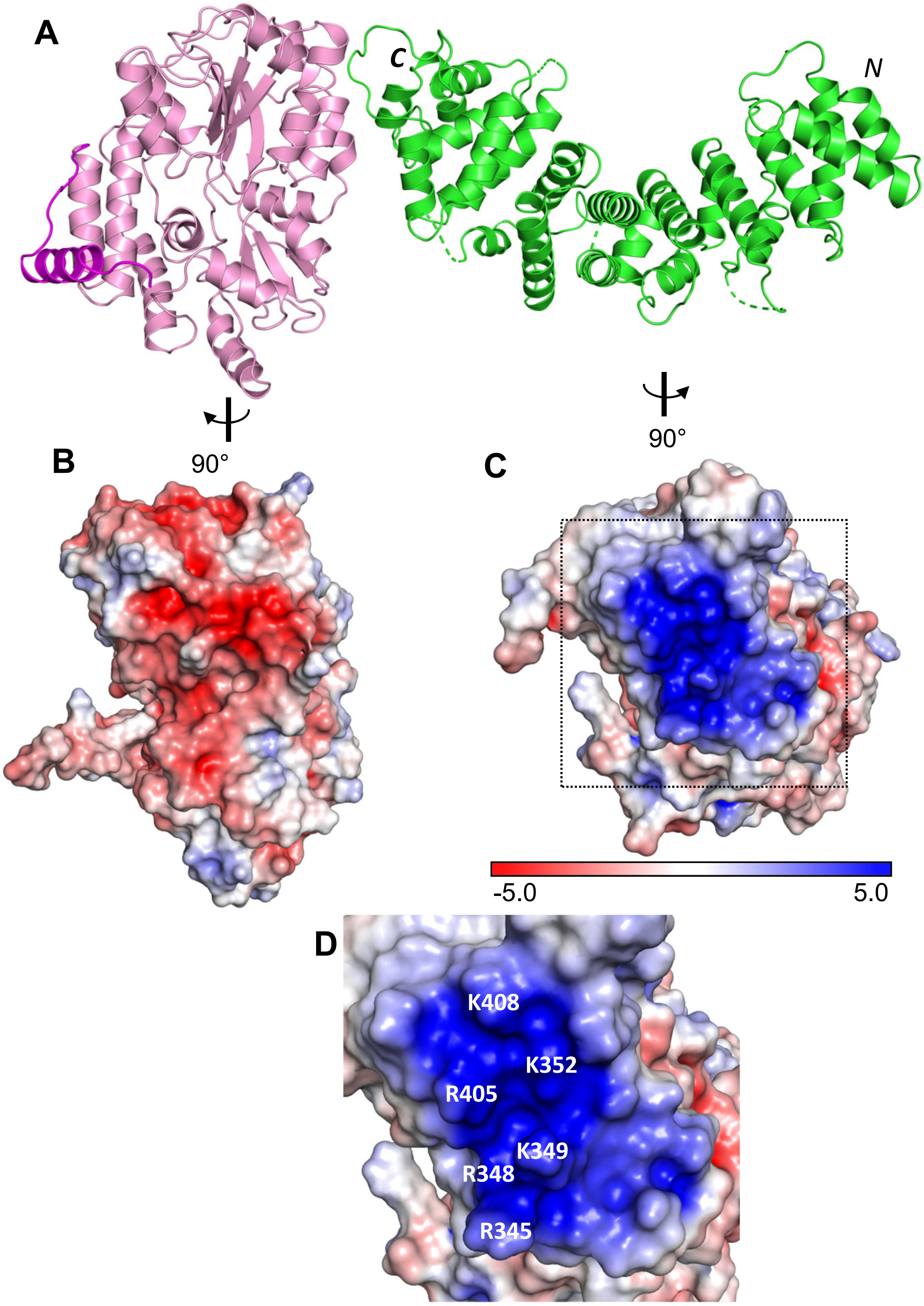

Supplementary Figure 5. Crystal packing in the Ric8A1-492/MBP-Gα_t327-350 crystal. (**A**) The positively charged region in the C-terminal part of the ARM domain of Ric8A packs against the negatively charged region of the MBP molecule from the neighboring asymmetric unit. (**B**) MBP-Gα_t327-350 is rotated along its y axis. (**C**) Ric8A is rotated along the y axis. Electrostatic surfaces are shown (units K_bT/e_c). (**D**) Close-up view of the positive surface boxed in (C).

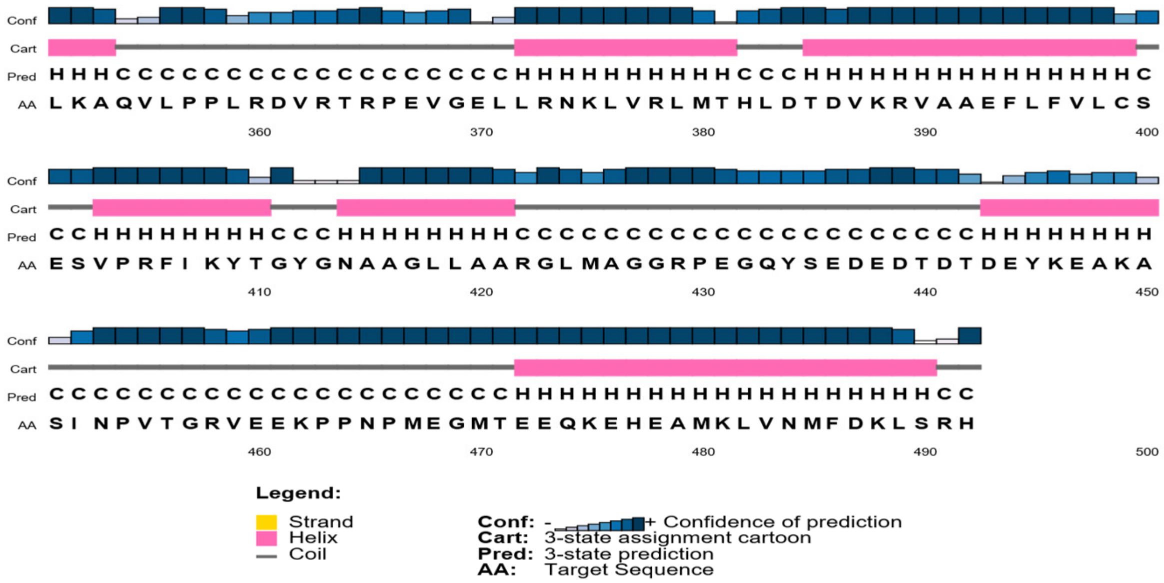

Supplementary Figure 6. Prediction of the secondary structure of the C-terminal tail of Ric8A. The prediction by PSIPRED server suggests that the C-terminal tail of Ric8A (residues 427-492) is largely unstructured with the exception of a short α-helix at residues 443-446 (low confidence) and a long α-helix at residues 471-489 (high confidence).

A

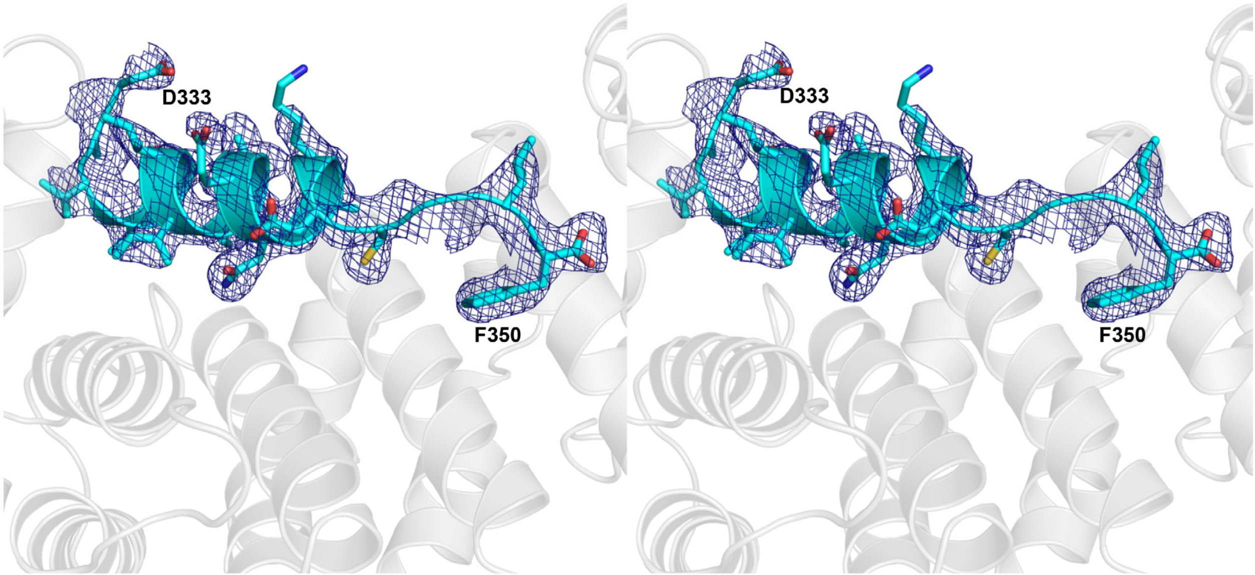

B

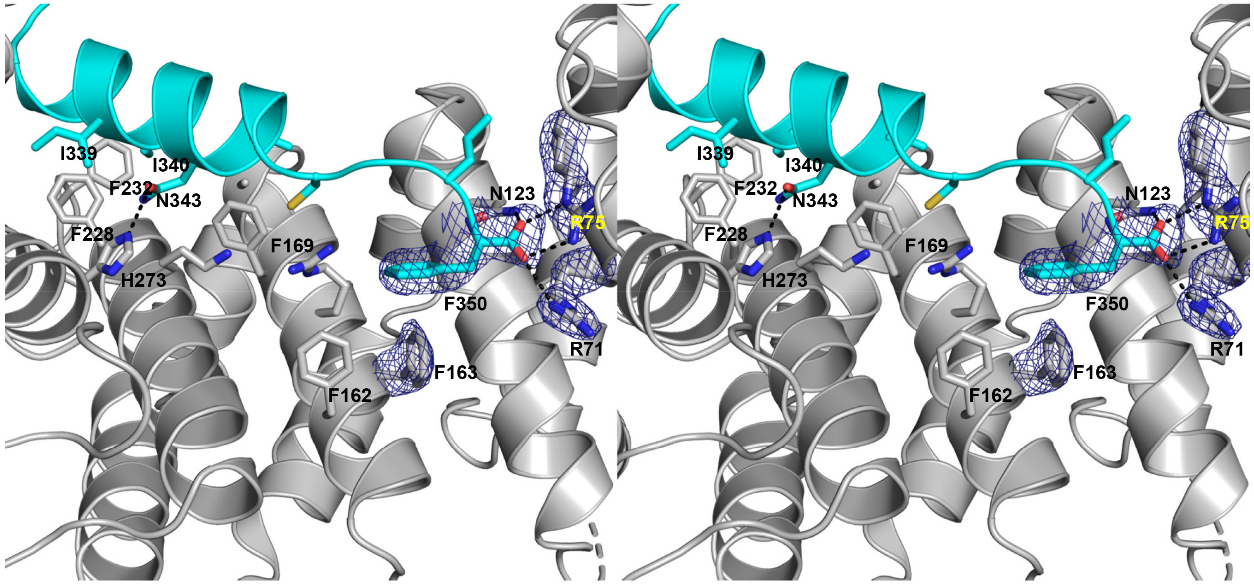

Supplementary Figure 7. Stereo images of Figures 3d and 3e.

(**A**) Fig. 3d. An omit map (Fo-Fc) for Gα_t333-350 is contoured at 2.5 σ and shown for 1.6 Å around the ligand. (**B**) Fig. 3e. Close-up of the interface between Ric8A (grey) and the Gα_t C-terminus (cyan). Omit maps (Fo-Fc) for Gα_t F350 and its critical Ric8A contact residues R71, R75, N123, and F163 are contoured at 2.5 σ and shown for 1.6 Å around the side chains.

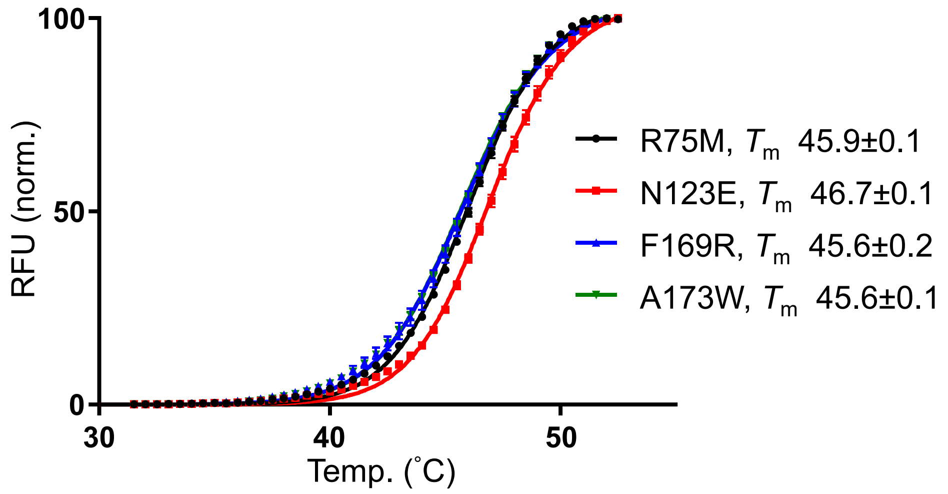

Supplementary Figure 8. Thermal stability of Ric8A1-492 mutants Average thermal denaturation curves of Ric8A1-492 mutants as determined by DSF. The T_m values (°C) are shown as mean±SD (n=4).

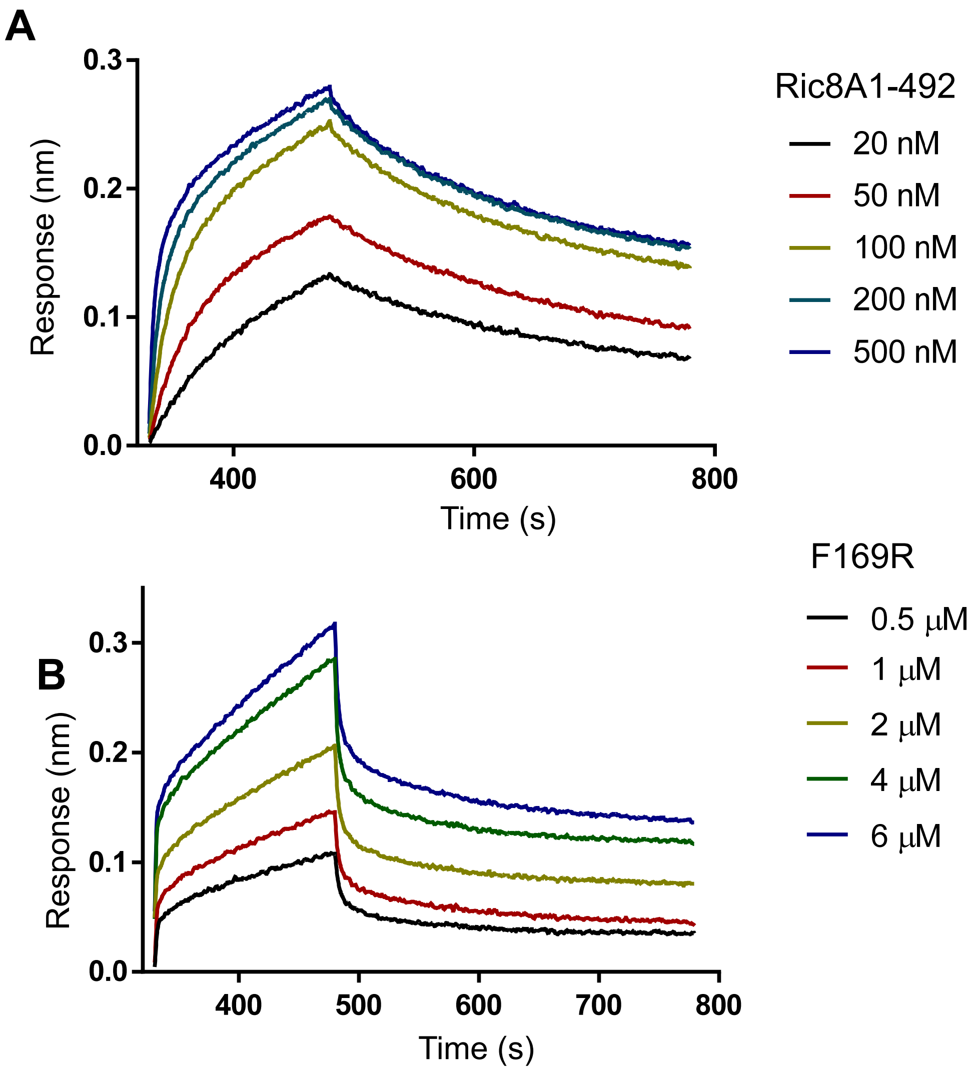

Supplementary Figure 9. Kinetics of association and dissociation of Ric8A1-492 (**A**) or its F169R mutant (**B**) and the Avi-tagged Gα_t coupled to a streptavidin biosensor as determined using BLI. Representative experiments are shown. The steady-state binding data are shown in Figure 3g.

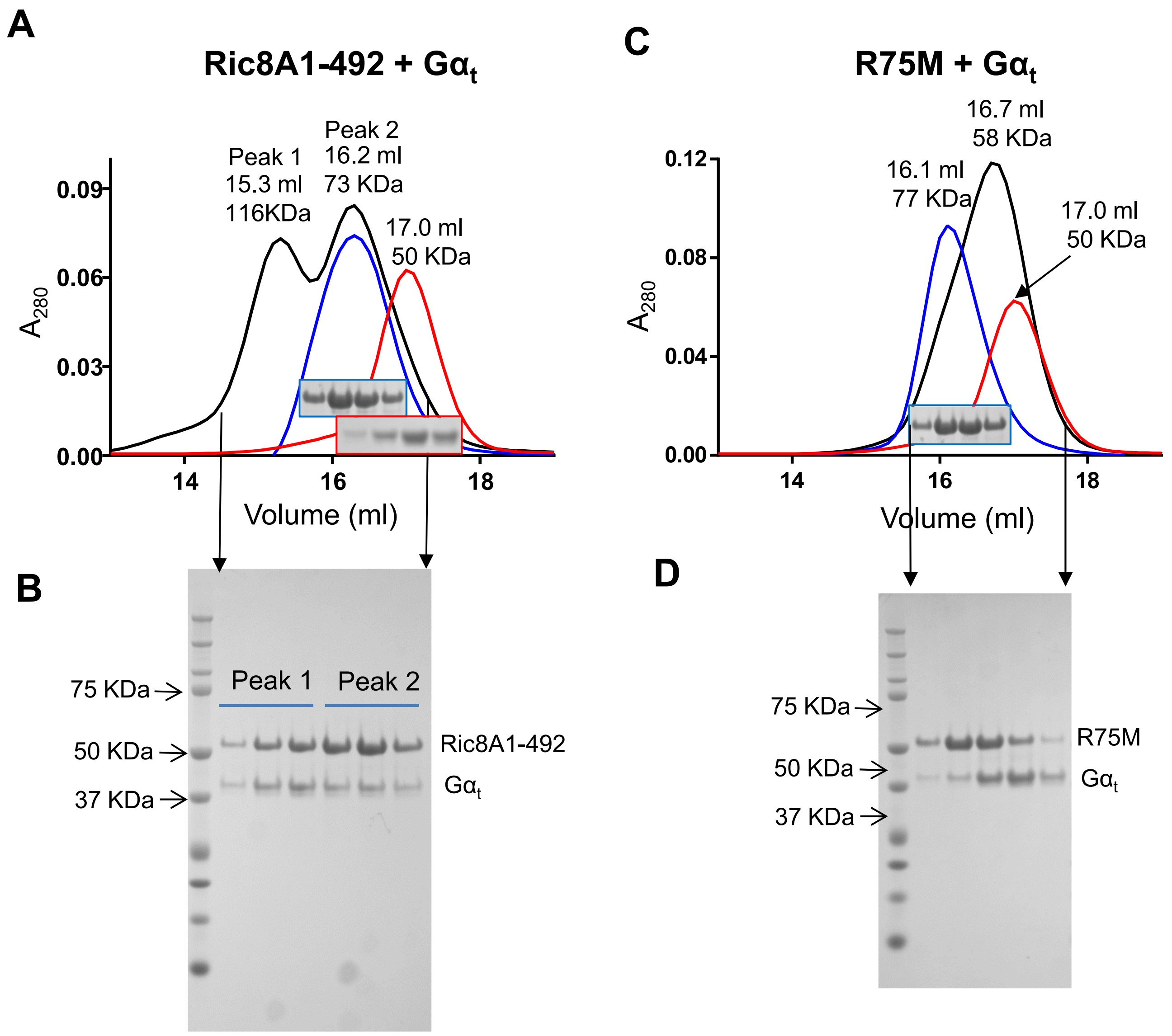

Supplementary Figure 10. The R75M mutant of Ric8A1-492 does not form a complex with Gα_t. (**A**) Ric8A1-492 was mixed with Gα_t and loaded onto a MW-calibrated Superdex 200 10/300 column. SEC profile - black curve. Blue curve and a gel in the inset with a blue outline represent SEC profile of Ric8A1-492 alone. Red curve and a gel in the inset with a red outline represent SEC profile of Gα_t alone. (**B**) Peak fractions in A analyzed by SDS-PAGE. Peak 1 containing the Ric8A1-492/Gα_t complex is not fully resolved from peak 2 corresponding to Ric8A1-492 alone. (**C**) The R75M mutant of Ric8A1-492 was mixed with Gα_t and loaded onto a MW-calibrated Superdex 200 16/600 column. SEC profile - black curve. Blue line and a gel in inset represent SEC profile of R75M alone. Red curve – SEC profile of Gα_t alone. (**D**) Peak fractions in C analyzed by SDS-PAGE. R75M and Gα_t are not resolved under these conditions and elute as a single peak with a shoulder corresponding to R75M. There is no peak corresponding to the R75M/Gα_t complex. First lane in (B) and (D) - MW markers.

A

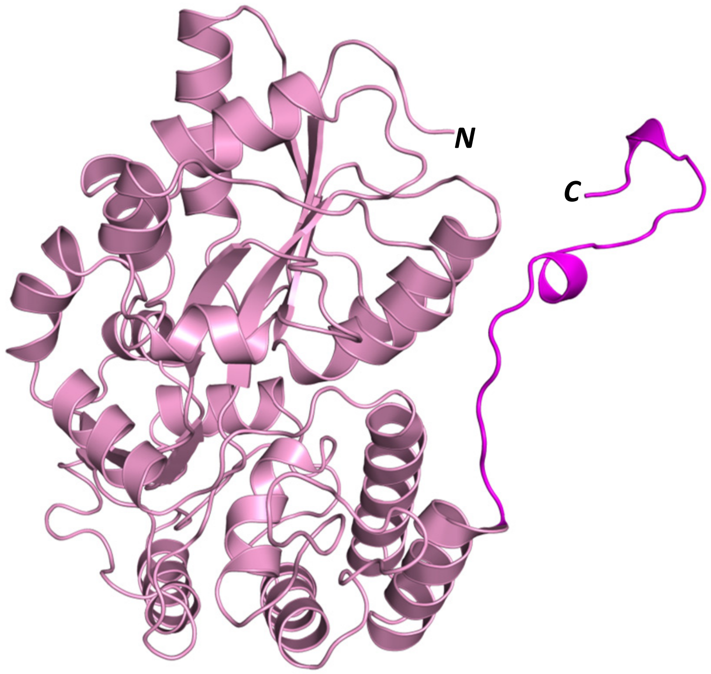

B

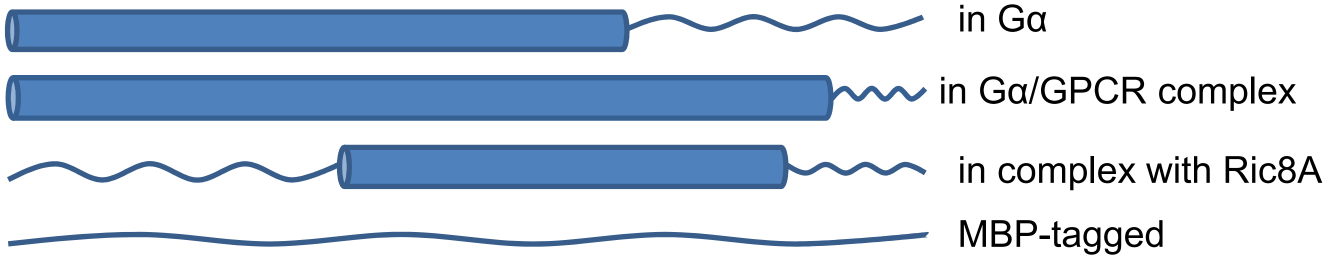

Supplementary Figure 11. Secondary structure of the C-terminal residues of Gα subunits in different crystal structures. (**A**) Crystal structure of MBP-Gα_t327-350. The Gα_t327-348 is colored magenta and MBP is colored pink. (**B**) The α5-helix is followed by eight to ten C-terminal residues that are unstructured or lack regular secondary structure in the GDP- and GTPγS-bound structures of Gα_t (PDBs 1TND, 1TAG, 3V00). In the complexes with GPCRs, the α5-helix extends towards the C-terminus (PDBs 3SN6, 6FUF, 6CMO, 6DDE, 6N4B). An α-helix is formed by residues Gα_t327-346 in the Ric8A/MBP-Gα_t327-350 complex (PDB 6N85). Gα_t327-350 largely lacks secondary structure in the crystal structure of MBP-Gα_t327-350 (PDB 6N84).

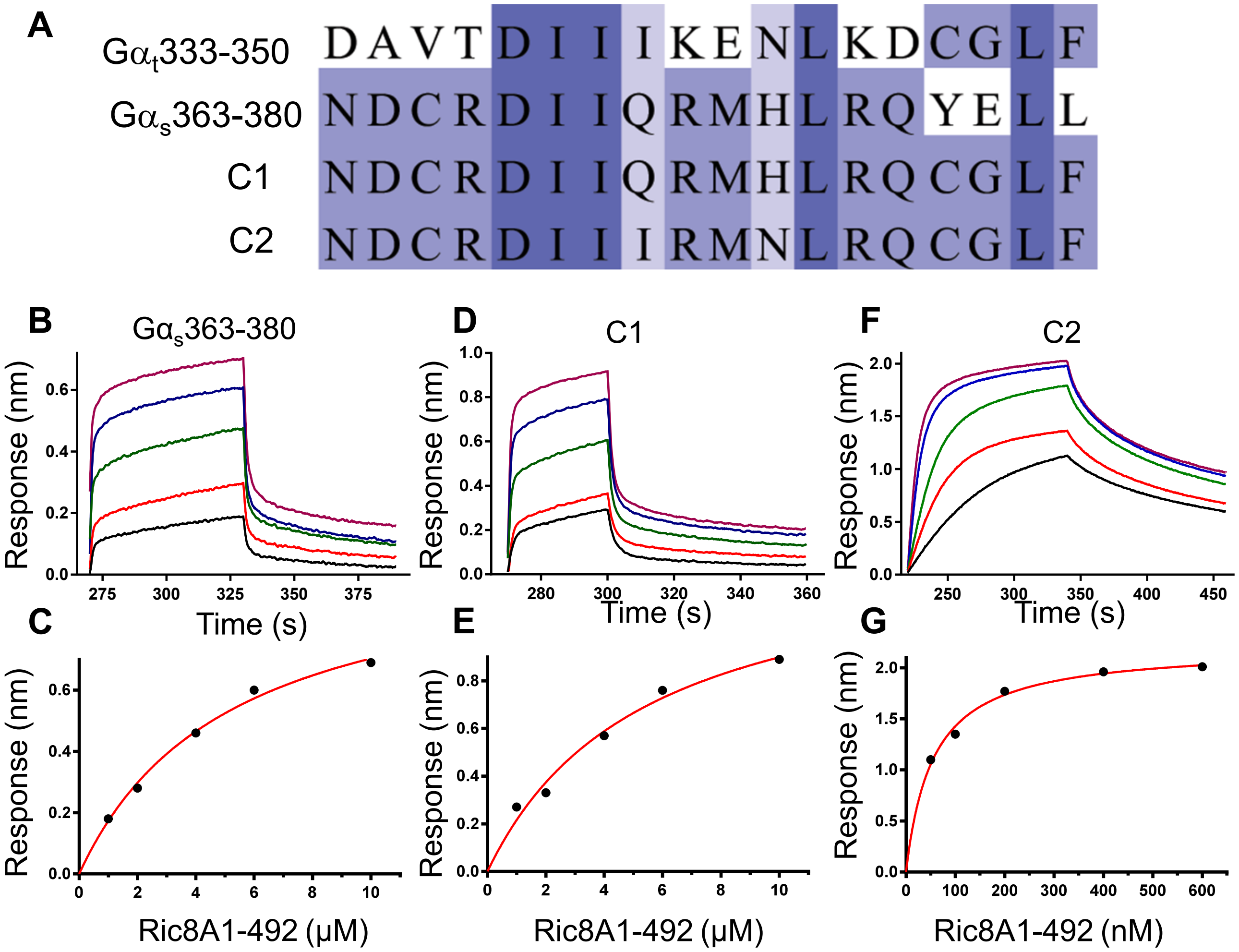

Supplementary Figure 12. Binding of Ric8A1-492 to the C-terminal Gα$_s$ and chimeric Gα$_{s/t}$ peptides. (**A**) Sequence alignment of Gα$_t$333-350, Gα$_s$363-380 and chimeric Gα$_{s/t}$ peptides C1 and C2. (**B,D,F**) Kinetics of association and dissociation for Ric8A1-492 and biotinylated peptides coupled to a streptavidin biosensor as determined using BLI. Representative curves are shown. (**C,E,G**) Steady-state binding curves as determined from data in (B,D,F). For experiments performed in triplicate; Gα$_s$363-380, K_D=4.4±0.8 μM; C1, K_D=4.7±0.6 μM; C2, K_D=63±28 nM (mean±SD).

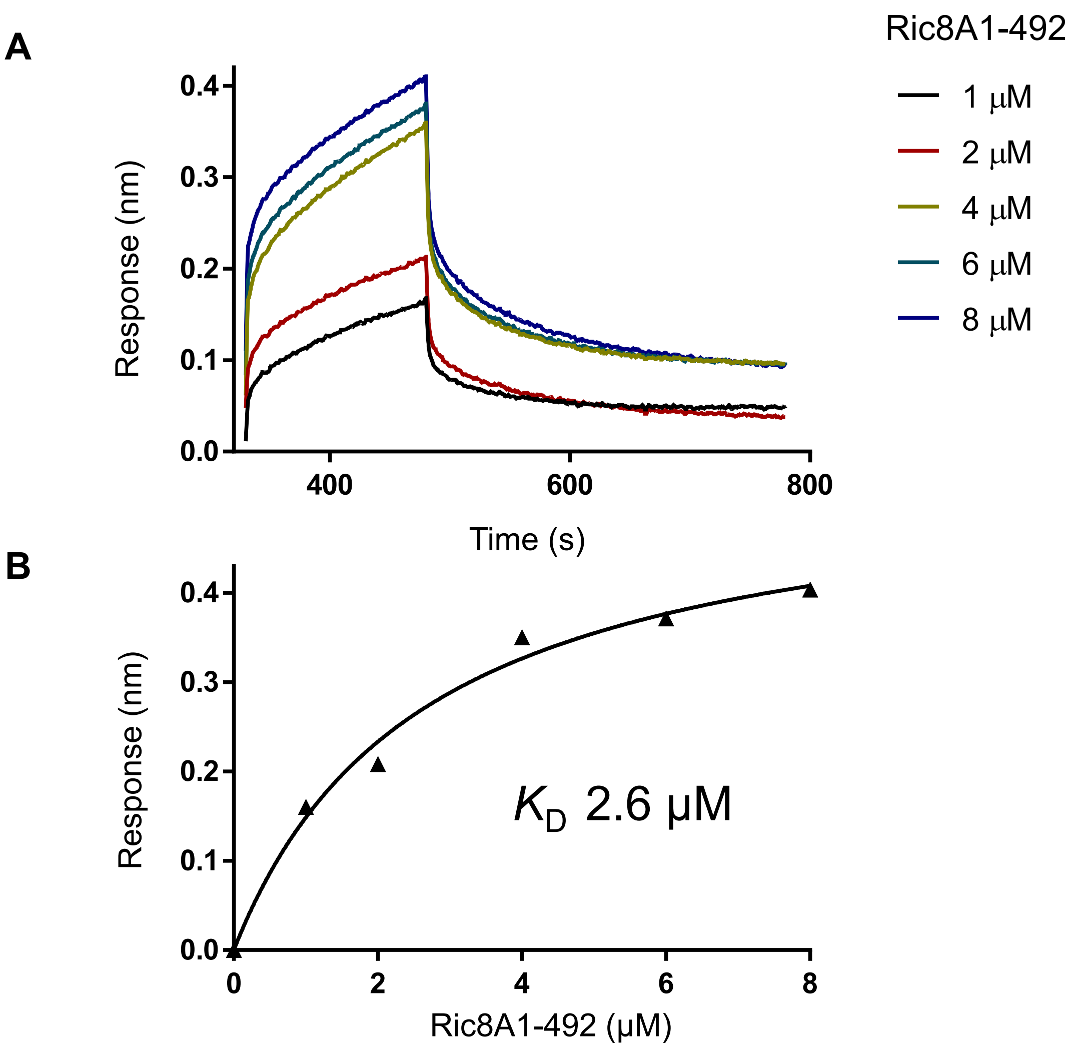

Supplementary Figure 13. (**A**) Kinetics of association and dissociation of Ric8A1-492 and the Avi-tagged I340Q/N343H mutant of $G\alpha_t$ coupled to a streptavidin biosensor as determined using BLI. Representative experiment is shown. (**B**) The steady-state binding curve. For experiments performed in triplicate K_D=2.3±0.4 μM (mean±SD).

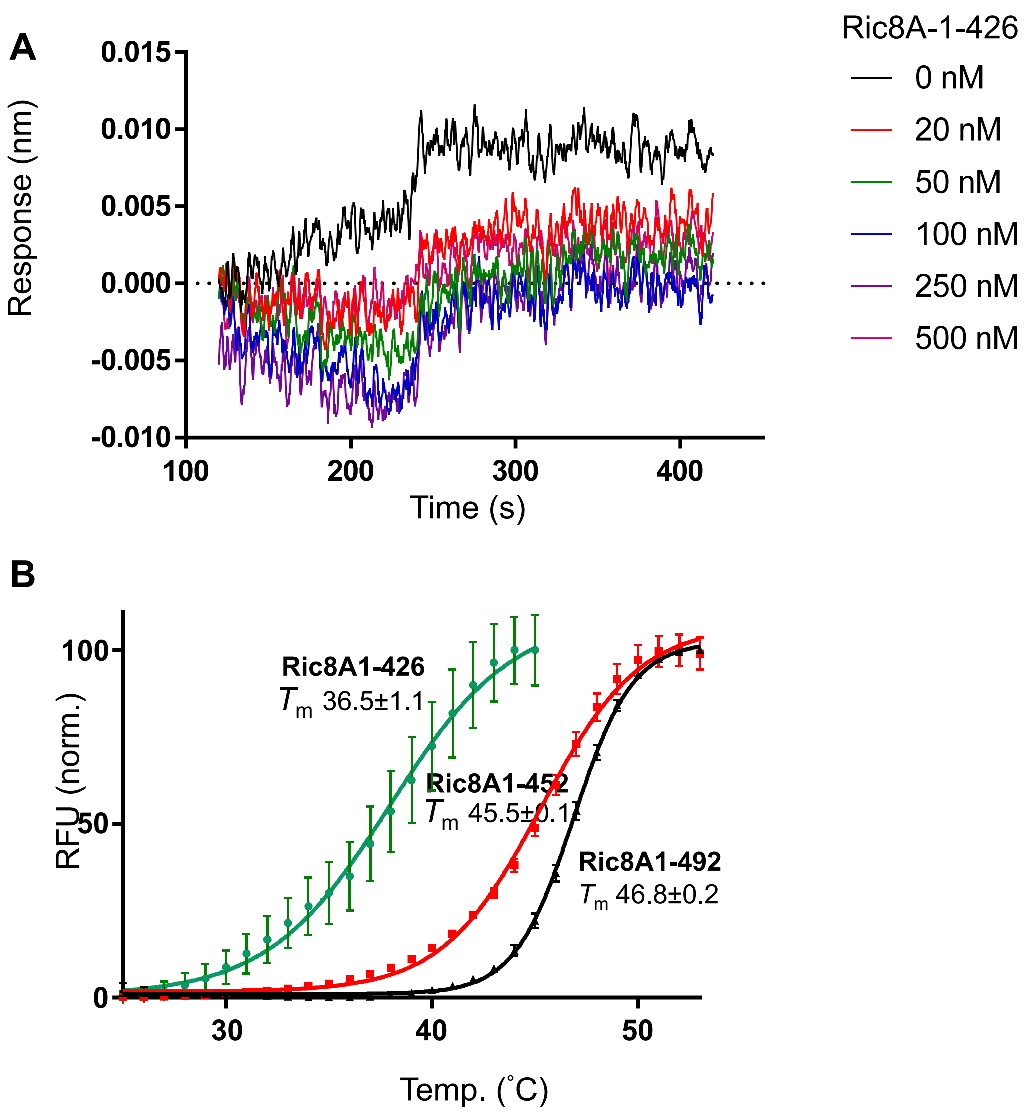

Supplementary Figure 14. Ric8A1-426 does not interact with Gα_t333-350. (**A**) The processed BLI data curves for Ric8A1-426 and biotinylated Gα_t333-350 coupled to a streptavidin biosensor. The BLI response curves are at the level of instrument noise, indicating no binding between Ric8A1-426 and Gα_t333-350 at these concentrations of Ric8A1-426 (**B**) Average thermal denaturation curve of Ric8A1-426 (green) as determined by DSF. The DSF data for Ric8A1-492 (black) and Ric8A1-452 (red) from figures 1A and 5A are included for comparison. The T_m values (°C) are shown as mean±SD (n=3).

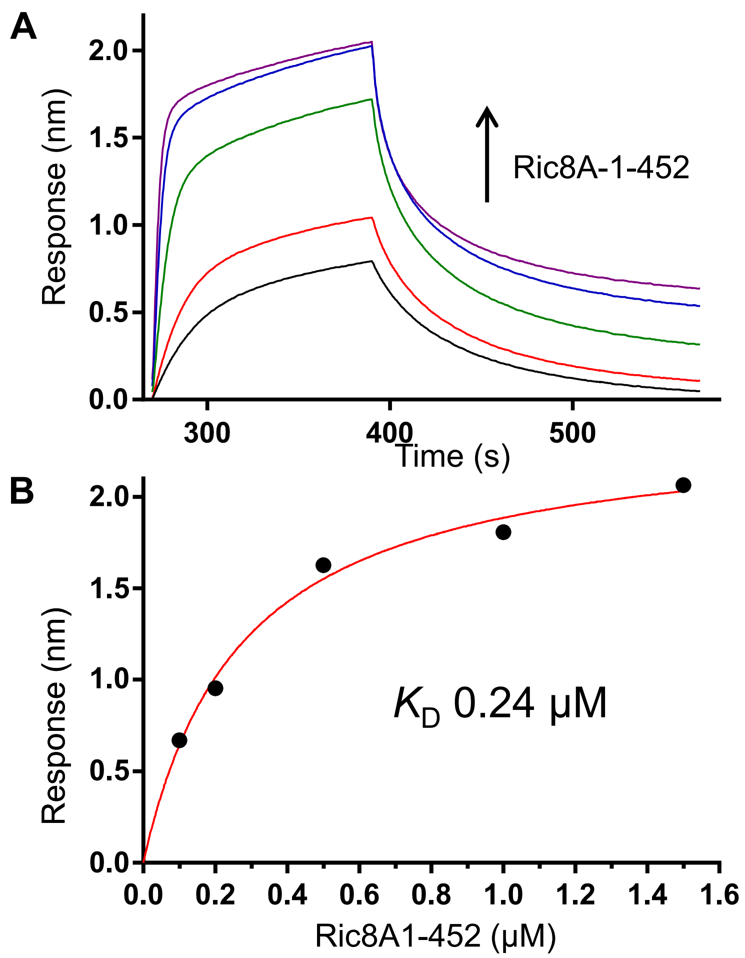

Supplementary Figure 15. Binding of Ric8A1-452 to Gα_t333-350. (**A**) Kinetics of association and dissociation for Ric8A1-452 and biotinylated Gα_t333-350 coupled to a streptavidin biosensor as determined using BLI. Representative processed data curves are shown. (**B**) Steady-state binding as determined from data in (A). For experiments performed in triplicate, K_D=0.24±0.03 µM (mean±SD).

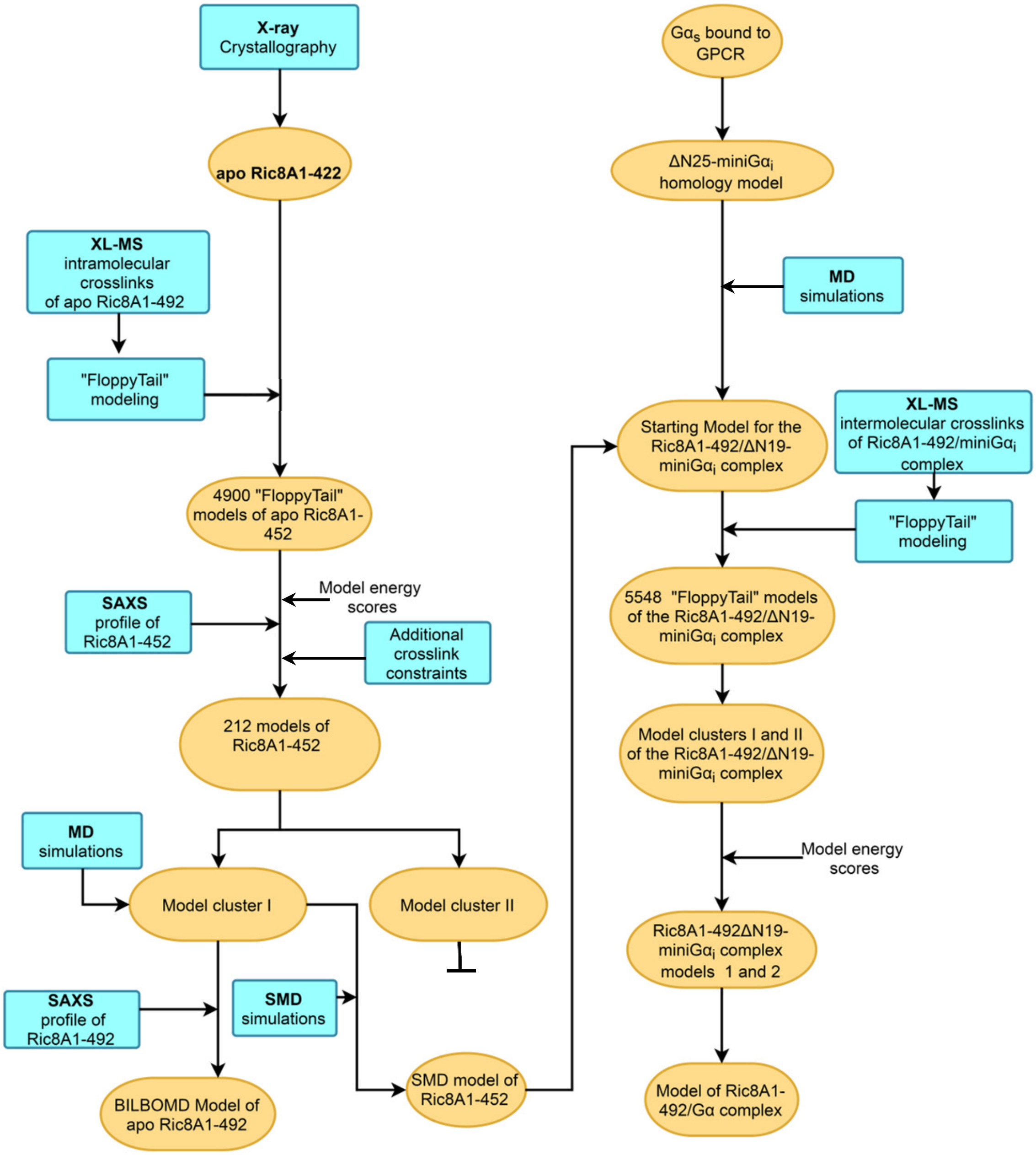

Supplementary Figure. 16. Flow chart of structural modeling of apo Ric8A1-492 and the Ric8A1-492/Gα complex.

Models of Ric8A1-452

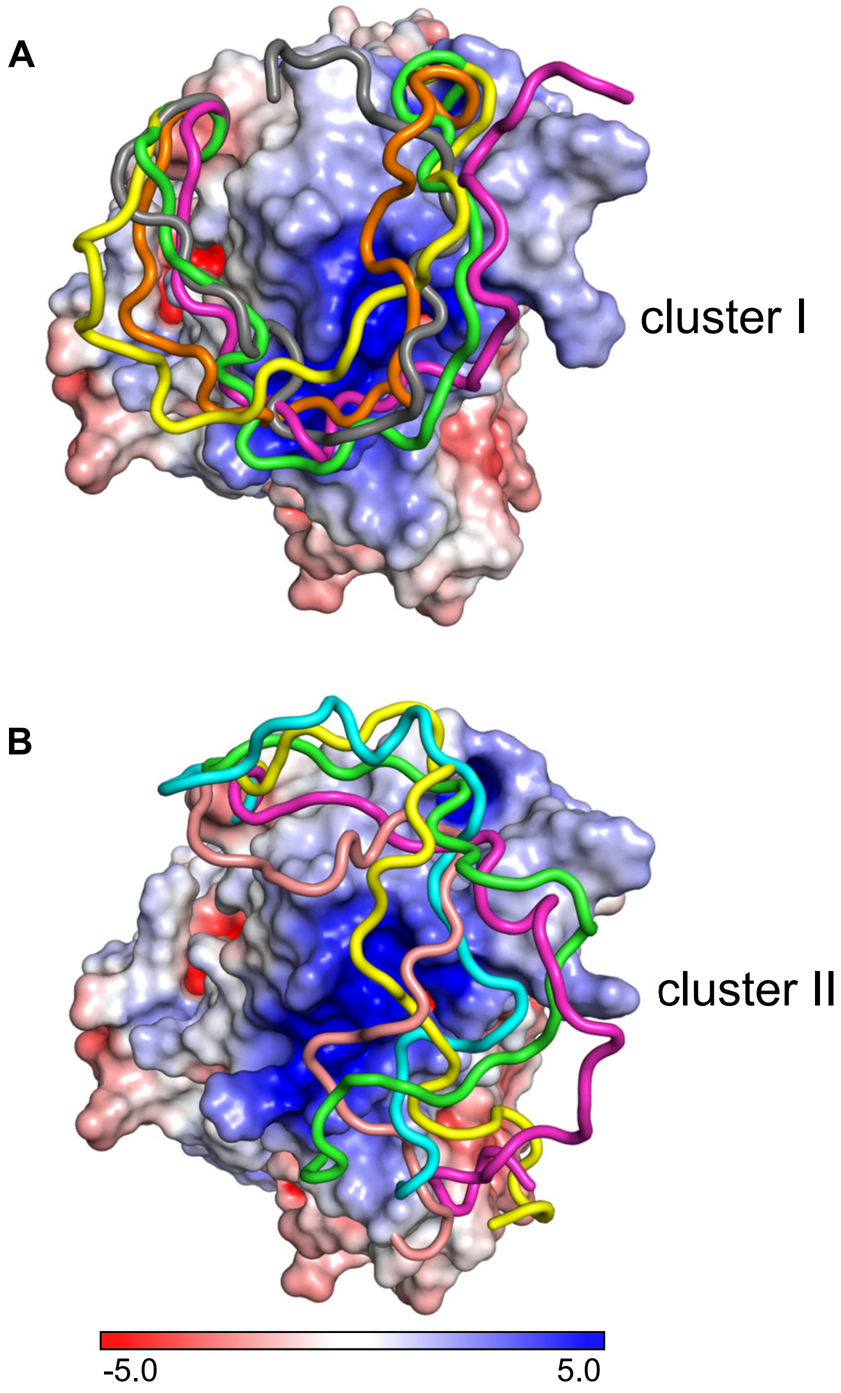

Supplementary Figure 17. Top energy score models of Ric8A1-452 from clusters I and II. Top five models of Ric8A1-452 from clusters I (**A**) and II (**B**) obtained by clustering 212 selected FloppyTail models. Electrostatic surface of Ric8A1-422 (units K_bT/e_c) is combined with the tube representation of the modelled C-terminal tail region Ric8A423-452.

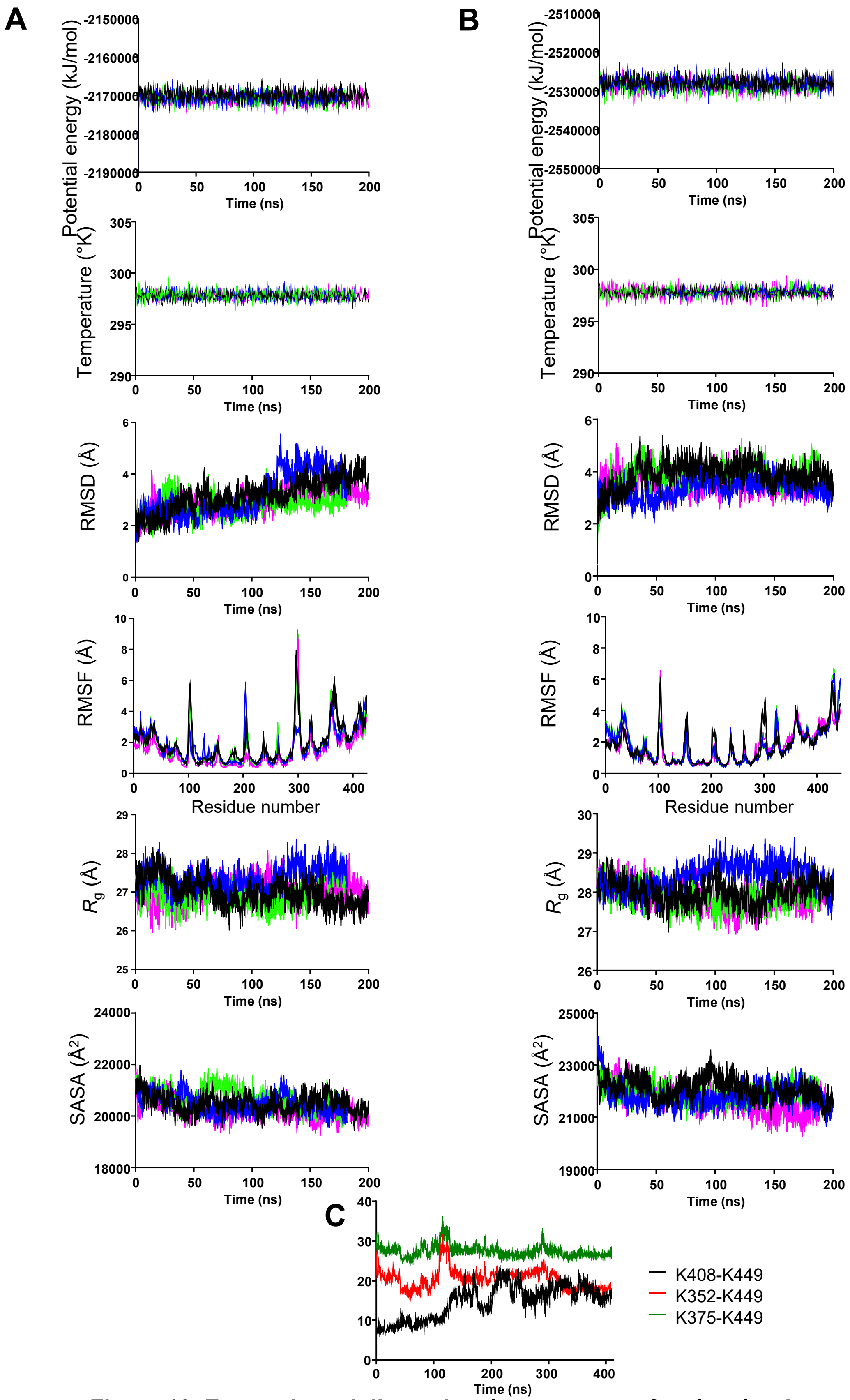

Supplementary Figure 18. Energetic and dimensional parameters of molecular dynamics (MD) simulations of Ric8A1-426 and the model of Ric8A1-452. MD simulation parameters (total potential energy, temperature, pressure, RMSD, RMSF, radius of gyration (R_g) and solvent accessible surface area (SASA)) are shown for each of the four 200-ns simulations of Ric8A1-426 (**A**) and Ric8A1-452 (**B**). (**C**) Distance evolution for the three crosslink pairs used in modeling/model selection during a 400-ns MD simulation of Ric8A1-452.

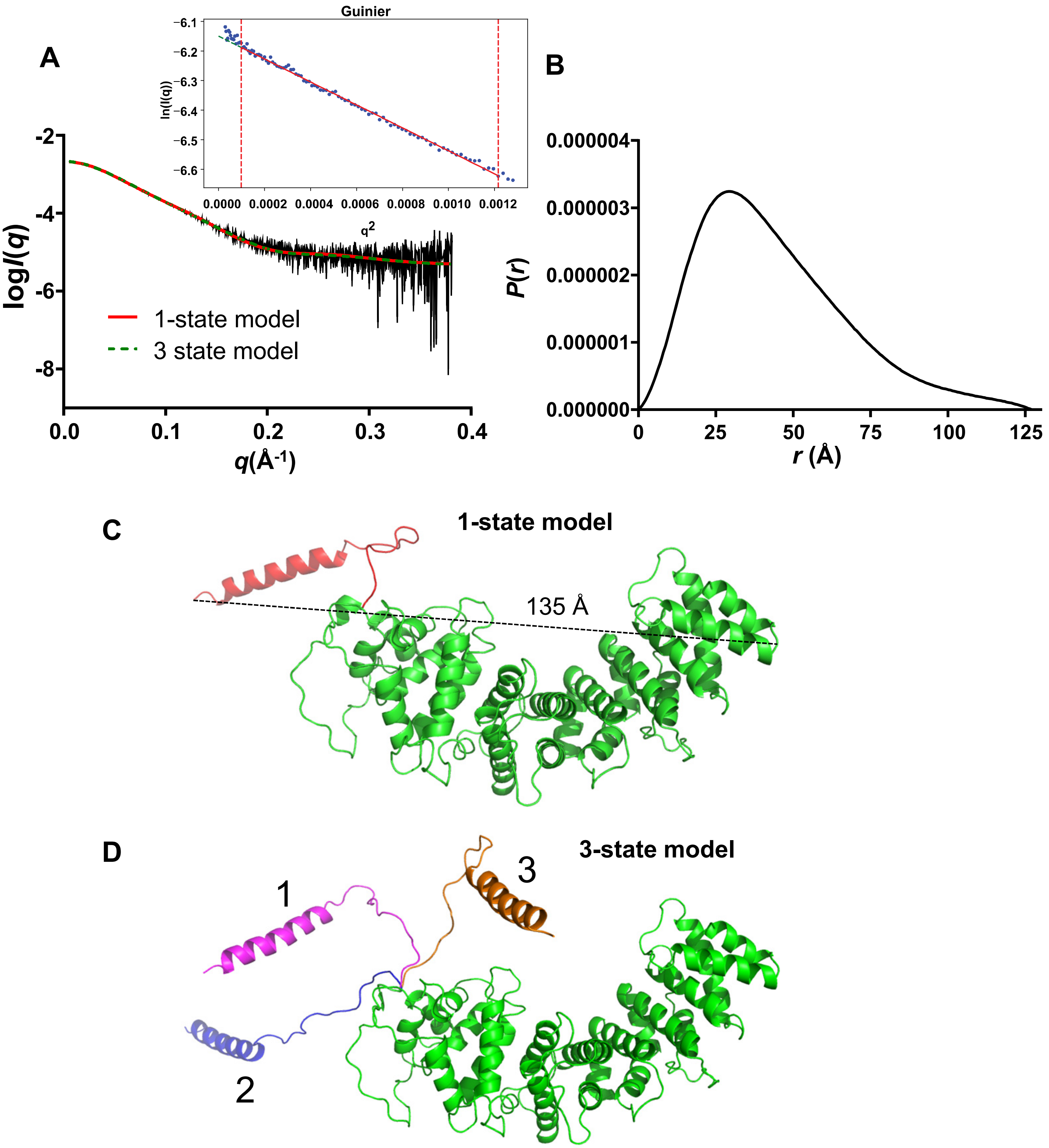

Supplementary Figure 19. The BILBOMD models of apo Ric8A1-492. (**A**) Experimental SAXS data for apo Ric8A1-492 (black curve). The theoretical SAXS profiles calculated for the 1-state (red curve) and 3-state (green dashed curve) models of apo Ric8A1-492 fit the data with χ^2 values of 1.51 and 1.19, respectively. The Guinier plot for the low q region ($q{\cdot}R_g < 1.3$) is shown in the inset. (**B**) The pairwise distance distribution function $P(r)$ indicates a an elongated molecule with a maximum dimension D_{max} of ~127 Å. (**C**) The best-scoring 1-state BILBOMD model features D_{max} of 135 Å. (**D**) The best scoring 3-state model consists of conformations 1, 2, 3 with the weights of 0.59, 0.29, and 0.12, respectively. Modeled residues 453-492 are shown in red (C), magenta, blue, or orange (D).

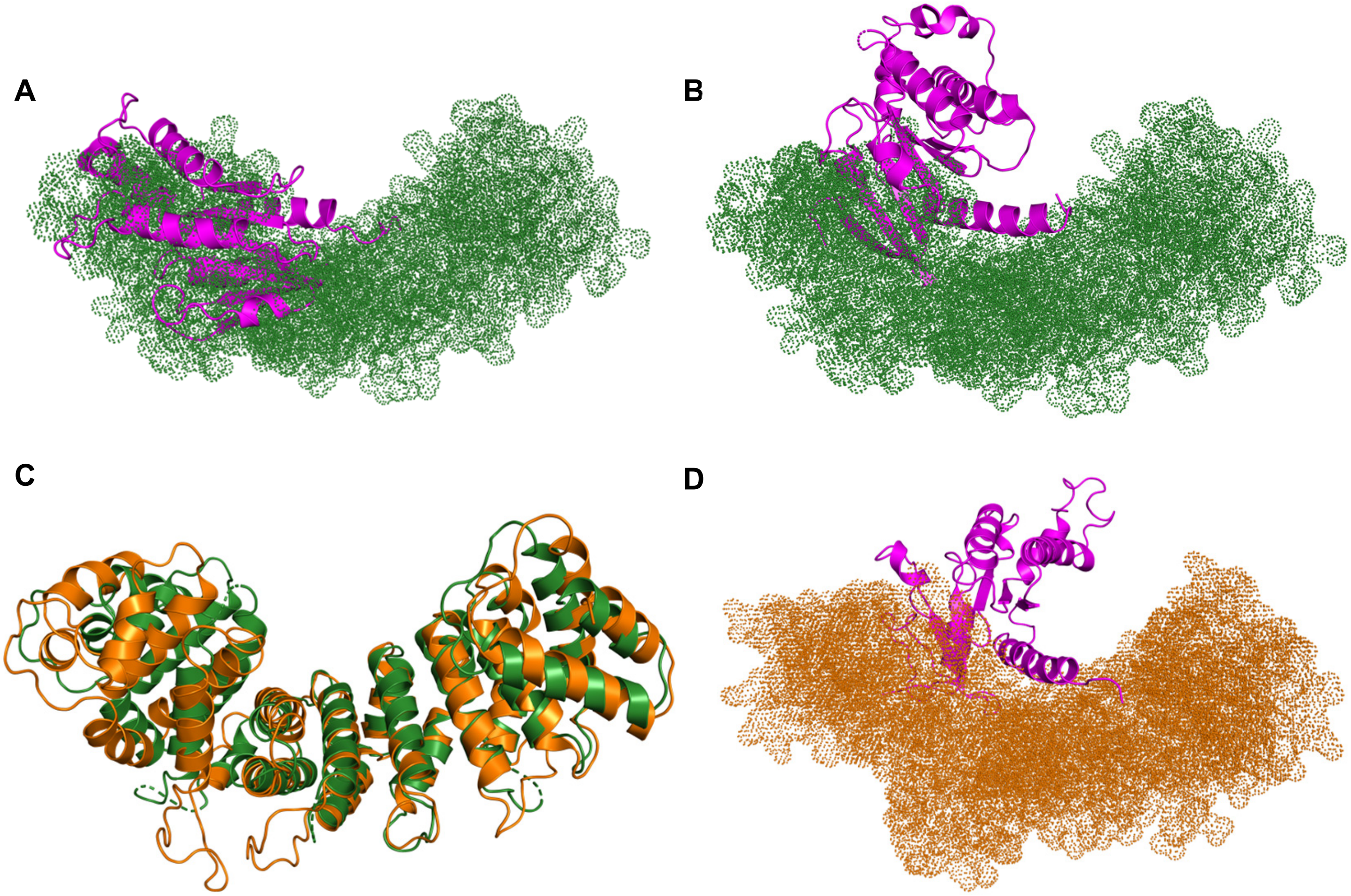

Supplementary Figure 20. Modeling of the Ric8A1-452/miniGα$_i$ complex. (**A**) Superimposition of the Gα$_t$ helical fragment from the Ric8A1-492/MBP-Gα$_t$327-350 structure with the α5 helix of GαGDP (PDB 1TAG) results in major steric clashes between Ric8A (green mesh) and the RD domain of Gα (magenta cartoon). There are no significant clashes with the HD domain of Gα, which is omitted for clarity. (**B**) The steric clashes are markedly reduced using the RD domain of GPCR-bound Gα$_s$ (PDB 3SN6). (**C**) Overlay of model 1 of Ric8A1-452 (green) with its "open" conformation after SMD simulation (orange) designed to mimic the forces that act on Ric8A on binding of ΔN25-miniGα$_i$. (**D**) There is no clashes between of Ric8A1-452 in open conformation and ΔN25-miniGα$_i$.

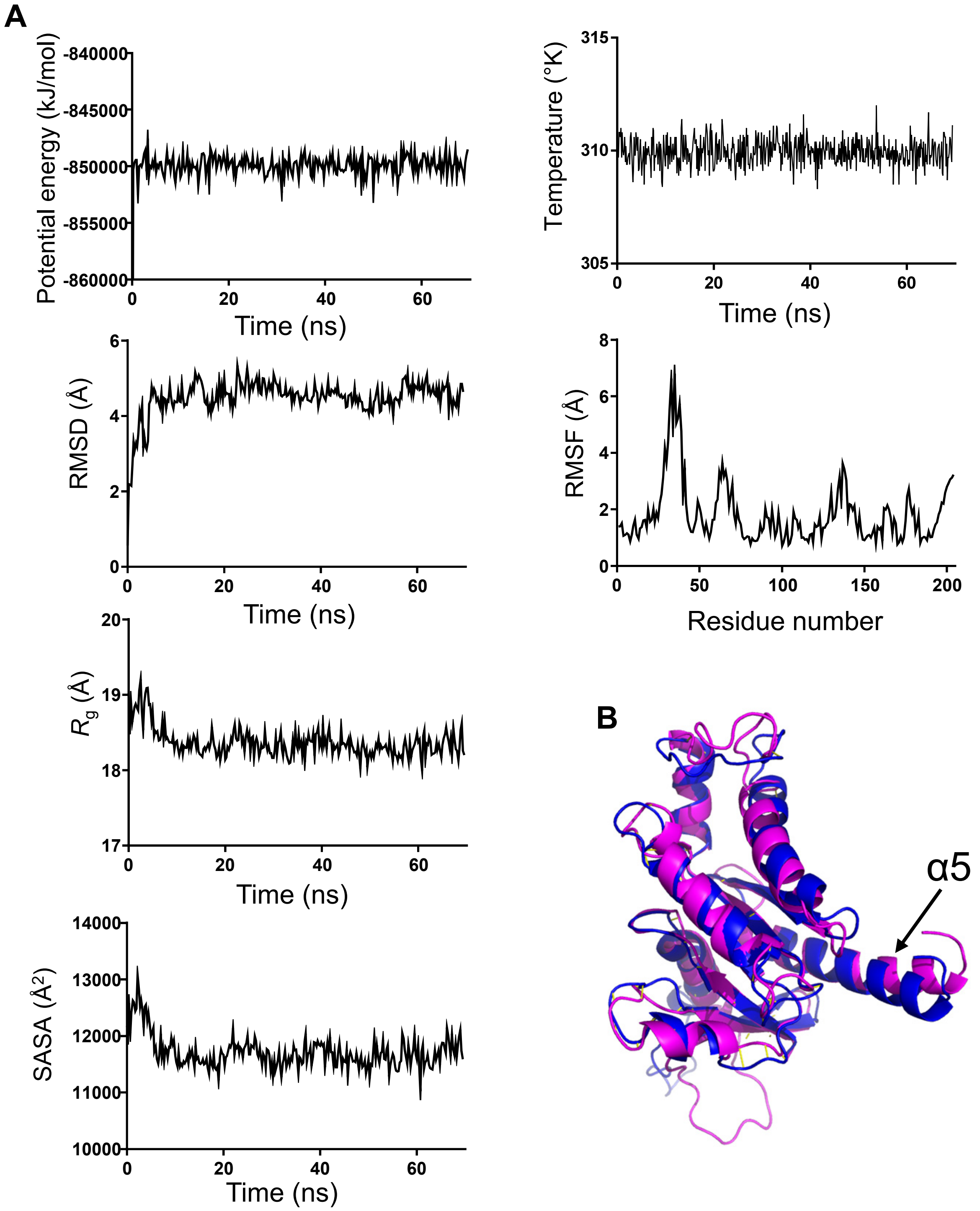

Supplementary Figure 21. Modeling ΔN25-miniGα$_i$. Energetic and dimensional parameters of MD simulation of ΔN25-miniGα$_i$. (**A**) MD simulation parameters (total energy, temperature, pressure, RMSD, RMSF, radius of gyration (R_g) and solvent accessible surface area (SASA)) are shown for the 70-ns simulation of a homology model of ΔN25-miniGα$_i$. (**B**) Overlay: blue - homology model of ΔN25-miniGα$_i$ before simulation; magenta - conformation of ΔN25-miniGα$_i$ (magenta) selected from the MD trajectory in (A) for modeling of the Ric8A1-452/miniGα$_i$ complex.

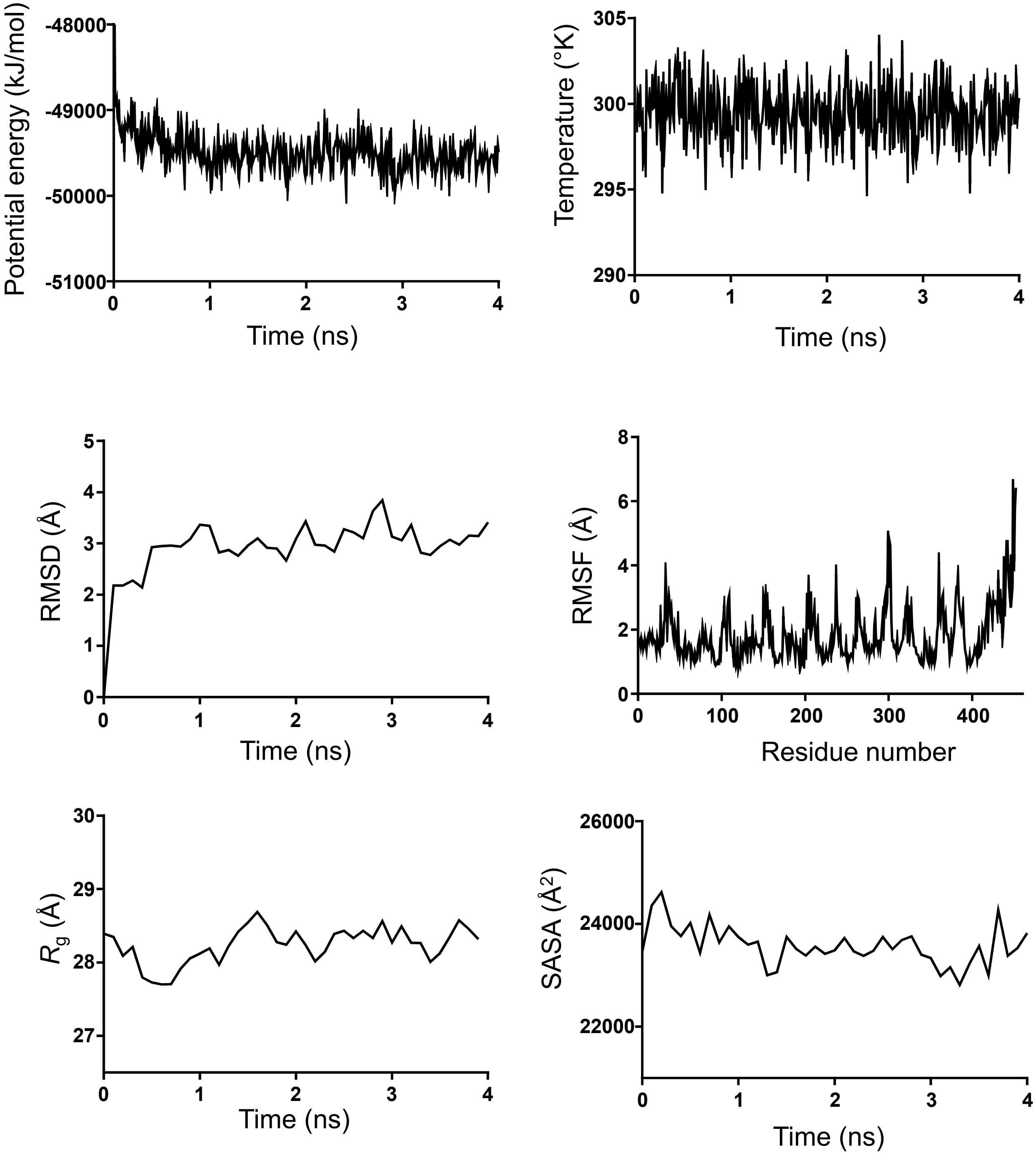

Supplementary Figure 22. Energetic and dimensional parameters of SMD simulation of the Ric8A1-452 model. Total energy, temperature, RMSD, RMSF, radius of gyration (R_g) and solvent accessible surface area (SASA) are shown for a 4-ns SMD of Ric8A1-452. The values of pressure are not available since the SMD was performed in Implicit Solvent model with no explicit definition of a simulation box.

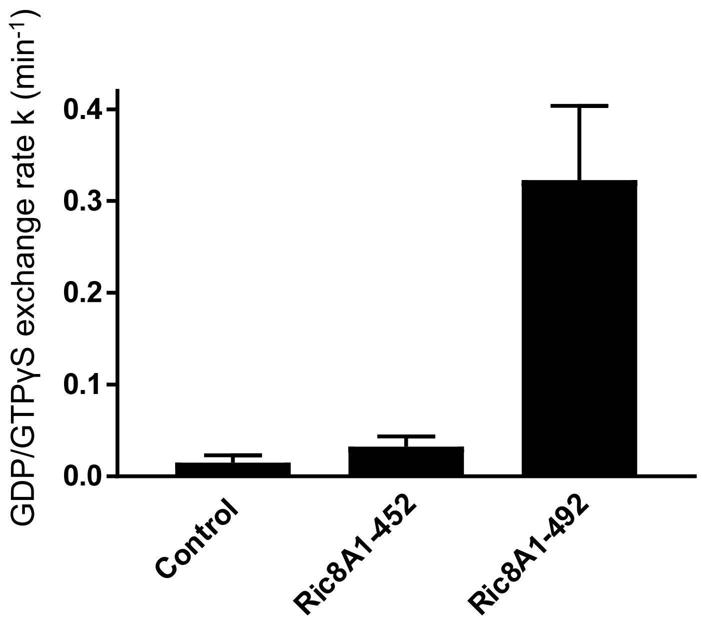

Supplementary Figure 23. The distal portion of the Ric8A C-terminal tail is important for its GEF activity. Rates of GDP/GTPγS exchange reaction on $G\alpha_i$ in the absence (control) or presence of Ric8A1-452 and Ric8A1-492 as measured from the kinetics of tryptophan fluorescence change of $G\alpha_i$ (mean±SD, n=3).

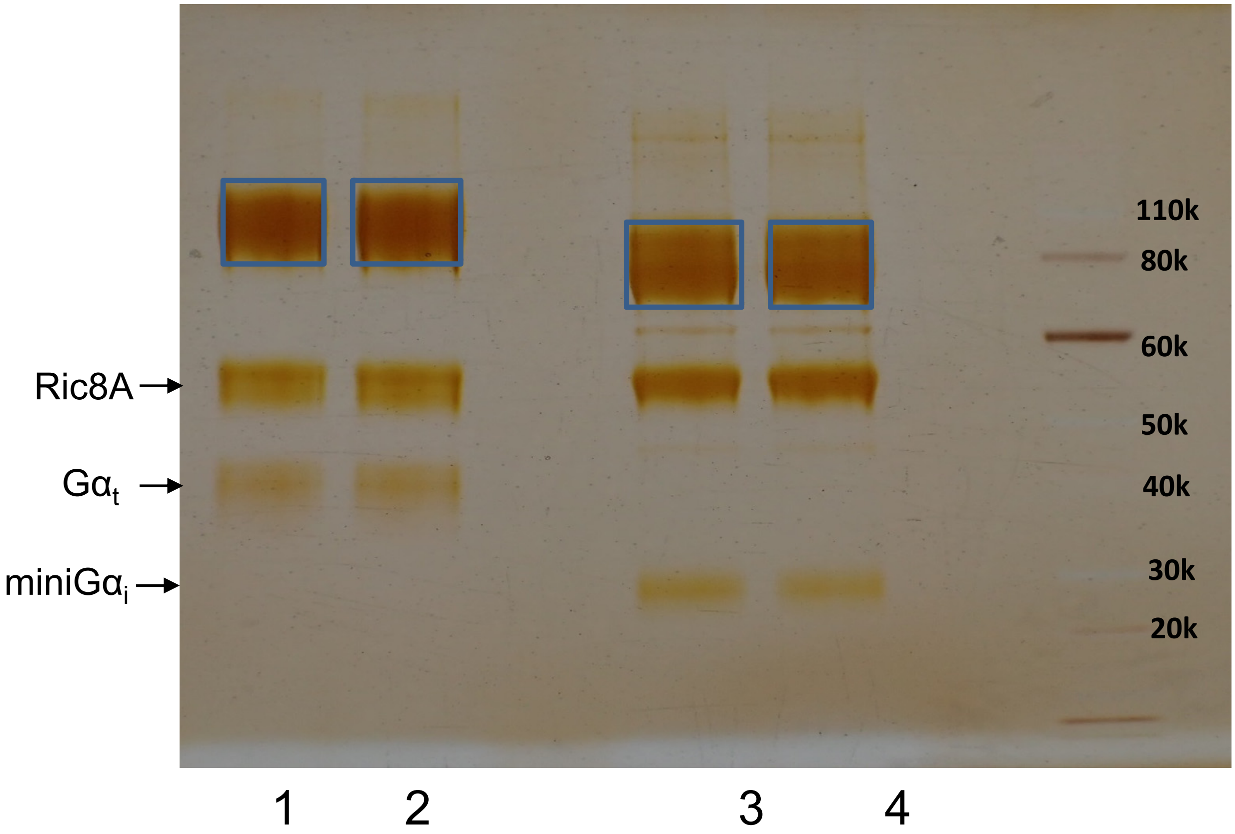

Supplementary Figure 24. Crosslinking of Ric8A1-492 to $G\alpha_t$ and $miniG\alpha_i$. Purified complexes Ric8A1-492/$G\alpha_t$ (lanes 1,2) and Ric8A1-492/$miniG\alpha_i$ were crosslinked using DSS and separated by SDS-PAGE. The crosslinked products (outlined) were excised from a silver-stained gel for in-gel digest with trypsin and LC-MS/MS.

.

Models of Ric8A1-492

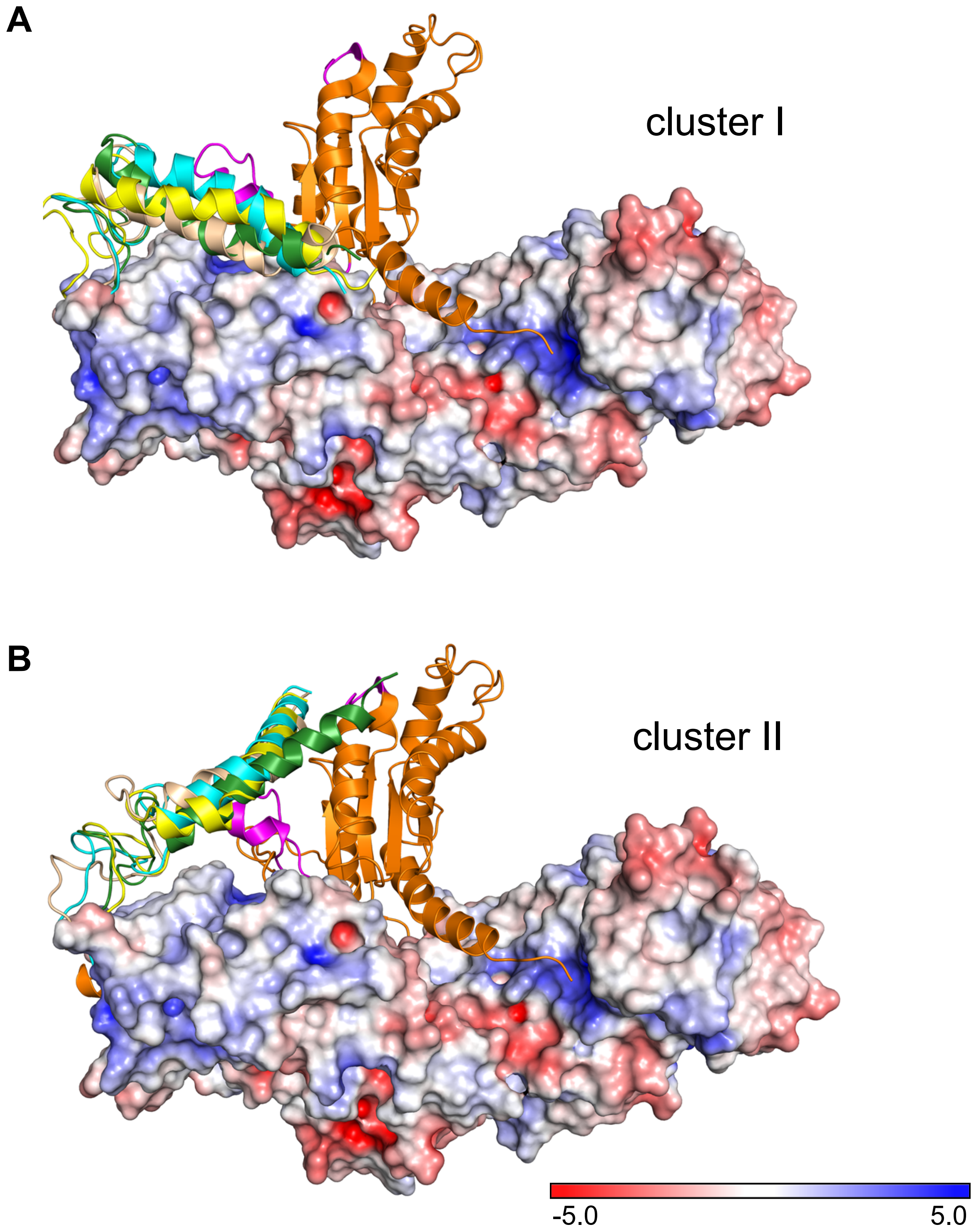

Supplementary Figure 25. Models of the Ric8A1-492/ΔN19-miniGα_i complex. Representative top models of the Ric8A1-492/ΔN19-miniGαi complex from clusters I (**A**) and II (**B**) obtained by clustering of the "FloppyTail" models. Electrostatic surface representation (units K_bT/e_c) of Ric8A1-452 is combined with the cartoon representations of the C-terminal tail region Ric8A453-492 (forest green, yellow, cyan and deep olive) and ΔN19-miniGαi (orange, switch II – magenta).

.

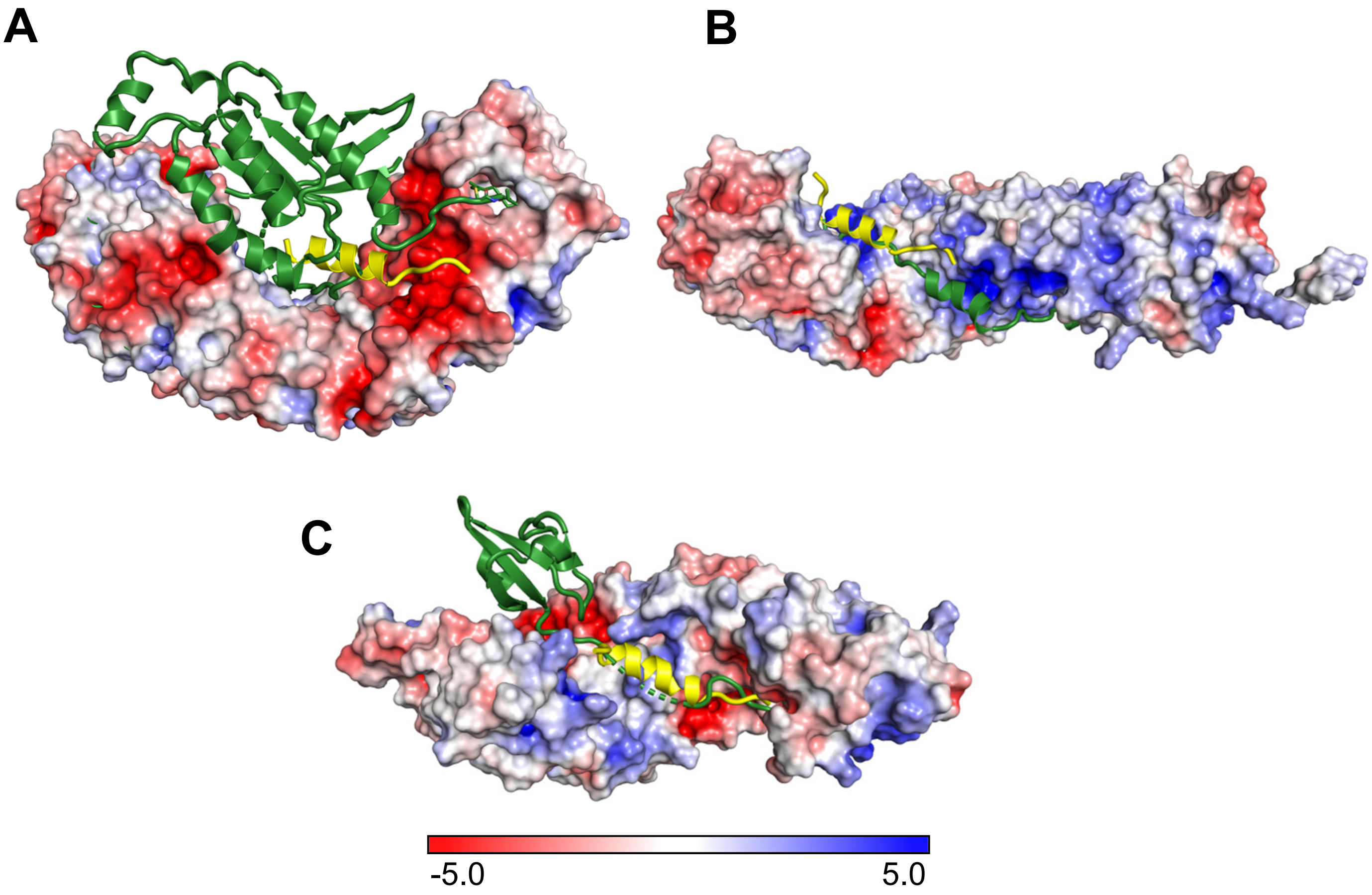

Supplementary Figure 26. Ligand binding by structural homologues of Ric8A. (**A**) Structure of SmgGDS in complex with farnesylated RhoA (PDB 5ZHX). RhoA is colored green and farnesyl group and cysteine is shown as sticks. SmgGDS is a GEF for the small GTPases RhoA and RhoC. A negatively charged region of SmgGDS interacts with a polybasic region near the prenylated C-terminus of RhoA, and a second interaction occurs between a positively-charged region of SmgGDS and the switch II region of RhoA, which becomes highly disordered. The second interaction site in SmgGDS partially overlaps with the surface of Ric8A that is involved in binding the Gα_t C-terminus. Gα_t333-350 (yellow) (A,B,C) is superposed from the structure of the Ric8A1-492/MBP-Gα_t327-350 complex.

B) Structure of β-catenin in complex with its ligand HTCF-4, a transcription factor of the Tcf/Lef-family (green) (PDB 1JDH). The Tcf/Lef binding site spans nearly the entire concave surface of β-catenin, and the region that binds the N-terminal fragment of the ligand overlaps with the Gα_t C-terminal binding site of Ric8A[47].

(**C**) Structure of importin-α in complex with the C terminal domain of influenza A virus polymerase PB2 subunit (green) (PDB 2JDQ). Two negatively charged nuclear localization signal (NLS)-binding sites on importin-α have been recognized; these are the major and minor binding sites in the N-terminal and C-terminal portions of the protein. The Ric8A Gα C-terminal binding site corresponds to the groove between the two NLS-binding sites of importin-α [50,51]

SmgGDS, importin-α and β-catenin are shown in electrostatic surface representation (units K_bT/e_c).

Supplementary Table 1. Data collection and refinement statistics (molecular replacement)

	MBP-Gα_t327-350 (PDB 6N84)	Ric8A1-492/MBP-Gα_t327-350 (PDB 6N85)	apo Ric8A1-492 (PDB 6N86)
**Data collection**			
Space group	P 63	P 21	F 222
Cell dimensions			
a, *b*, *c* (Å)	83.1 83.1 109.2	65.8 92.0 86.3	95.6 134.3 221.8
α, β, γ (°)	90.0 90.0 120.0	90.0 112.3 90.0	90.0 90.0 90.0
Resolution (Å)	60.1-1.75 (1.84-1.75) *	60.3-2.50(2.64-2.50)*	57.5-3.90(4.11-3.90)*
R_{merge}	0.062(0.753)	0.060(0.554)	0.097(0.799)
R-meas	0.069(0.850)	0.071(0.664)	0.107(0.867)
R-pim	0.021(0.277)	0.026(0.250)	0.028(0.237
CC1/2	1.0(0.809)	0.999(0.929)	0.999(0.955)
CC*	1.0(0.945)	1(0.977)	1(0.99)
$I / \sigma I$	29.0(2.5)	21.9(3.2)	18.4(3.3)
Completeness (%)	100.0(100.0)	99.8(99.7)	99.9(100.0)
Redundancy	10.7 (9.3)	7.3 (6.9)	14.3 (13.3)
Total reflection	460070 (58704)	240572 (33146)	95266 (12660)
Unique reflection	43096 (6311)	32979 (4820)	6668 (954)
Wilson B –factor (Å^2)	20.7	44.7	148.4
**Refinement**			
Reflections used in refinement	43058(4304)	32861(3269)	6642(644)
Reflections used for R-free	2076(209)	1664(195)	297(27)
R_{work} / R_{free}	15.65/ 18.49	19.64/24.89	25.58/29.45
CC(work)	0.968(0.893)	0.963(0.898)	0.944(0.854)
CC(free)	0.973(0.814)	0.949(0.782)	0.934(0.840)
No. atoms			
Protein	3058	6221	3157
Ligand/ion	63	23	0
Water	478	95	0
Protein Residues	392	796	399
B-factors	25.58	58.24	191.1
Protein	23.52	58.45	191.1
Ligand/ion	39.43	44.59	
Water	36.92	47.58	
R.m.s. deviations			
Bond lengths (Å)	0.006	0.002	0.001
Bond angles (°)	0.8	0.5	0.4

*Values in parentheses are for highest-resolution shell.

Supplementary Table 2. SAXS data-collection and scattering-derived parameters

**(a) Sample details**	Ric8A1-452	Ric8A1-492
SEC-SAS loading volume/concentration, flow rate	250 µl, 10 mg/ml, 0.9 ml/min	250 µl, 10 mg/ml, 0.9 ml/min
**(b) SAXS data collection parameters**		
Source, instrument and description or reference	Advanced Photon Source, Bio-CAT beamline 18-ID-D Pilatus 3X 1M pixel detector (DECTRIS)	
Wavelength	1.033 Å	
Sample to detector distance	3.5 m.	
q-measurement range (Å^{-1} or nm^{-1})	0.005 – 0.388	
Exposure time	0.5 second every 3 second	
Sample configuration including path length and flow rate where relevant	quartz Capillary diameter 1.5 mm with 10 µM wall	
Sample temperature	Room temperature	
**(c) Software employed for SAXS data reduction**		
SAXS data reduction	BioXTAS RAW	
Basic analyses: Guinier, P(r), Porod volume, pair distribution	BioXTAS RAW, ATSAS	
Calculation and comparison of scattering data	CRYSOL	
**(d) Structural parameters**		
Guinier Analysis		
R_g	29.6 ± 0.1	34.0 ± 0.1
qR_g range	0.30-1.26	0.34-1.20
Quality-of-fit parameter (R^2)	0.998	0.997
P(r) analysis		
R_g	30.4 ± 0.1	35.0 ± 0.1
D_{max}	110	127
q range	0.01-0.25	0.01-0.25
Quality-of-fit parameter (χ^2)*	1.18	1.28
**(e) Data deposition IDs**	SASDF75	SASDF65

* for the fits of the pair distance distribution functions to the experimental data

Supplementary Table 3. List of primers used in this study

Primer Purpose	Primer Sequence (5' to 3')	
	Forward	Reverse
Ric8A		
1-492	AATTATCATATGGAGCCCCGGGCAGTTG	TAAATTCTCGAGTTAGTGCCTGGAAAGCTTGTCGAAC
1-452	AATTATCATATGGAGCCCCGGGCAGTTG	ATTTTAGGATCCTTATATGCTGGCCTTGGCTTCC
1-425	AATTATCATATGGAGCCCCGGGCAGTTG	AATTTAGGATCCTTATGCCATGAGGCCCCTAGC
$^{460}EEK^{462} \rightarrow AAA$	GGGTAGCGGCAGCGCCTCCCAACCCCATGGA	GGAGGCGCTGCCGCTACCCTTCCAGTCACTGGG
R75M	CATCTTGTCCATGGACCGCAGCTGCCTG	GCAGCTGCGGTCCATGGACAAGATGCGG
N123E	CCTTAAGTGCCTGTGCGAGCTCGTGCTAAGCAGCC	GGCTGCTTAGCACGAGCTCGCACAGGCACTTAAGG
F169R	CTTTGACTTGCGTCTTCTCCGCTTGCTAACTGCACTCCGC	GCGGAGTGCAGTTAGCAAGCGGAGAAGACGCAAGTCAAAG
A173W	GCGTCTTCTCTTCTTGCTAACTTGGCTCCGCACCGACGT	ACGTCGGTGCGGAGCCAAGTTAGCAAGAAGAGAAGACGC
$G\alpha_t$ 1-350	TTAATTCCATGGGGGCTGGGGCCAGC	AATTTACTCGAGTCAGAAGAGCCCGCAGTCTTTG
Avi tag	CTTCGAAGCTCAGAAAATCGAATGGCACGAAGAAAACCTG TATTTTCAGGGC	GATTTTCTGAGCTTCGAAGATGTCGTTCAGACCGGTCGTTG GGATATCGTAATC
I340Q/N343H	ATCCAGAAGGAGCACCTCAAAGACTGCGGGCTC	GAGGTGCTCCTTCTGGATGATGTCGGTGACAGC
$G\alpha_i$	AATTTACCATGGGCTGCACACTGAGCGC	TTAAATCTCGAGTTAGAAGAGACCACAGTCTTTTAG
mini$G\alpha_i$	TAAATACCATGGACAAGGCGGCCGTGG	CAATTGTGCTCTTCCCGGAGTTATCAGCACCCAGCAGCAGC
	CGAGGCTGGCTACTCAGAGGGTGGCAGCACCCAGCAGGA TGTTCTC	ACAGAAGATGATGGCAGCCACGTCTTCAAAGCAGTGAATC
	GCTGCCATCATCTTCTGTGTGGACCTGAGTGACTATGAAG AAATGAACCGGATGC	TTAAATCTCGAGTTAGAAGAGACCACAGTCTTTTAG
MBP-$G\alpha_t$ peptide	CCCCTCACGATATGCTATCAAGAATATGCAGGCTCAAAC*	
	AATATATTACATATGAAAATCGAAGAAGGTAAACTGGTAAT CTG*	
	GGCTGAAATCACCCCGGCCGCAGCGTTCCAGGACAAGC	GCTTGTCCTGGAACGCTGCGGCCGGGGTGATTTCAGCC
	GGGGGTTATGCGTTCAAGTATGCAGCCGGCAAGTACGAC ATTAAAGA	TCTTTAATGTCGTACTTGCCGGCTGCATACTTGAACGCATA ACCCCC
	GTCCAACATCGACACCAGCGCAGTGAATTATGGTGTAACG	CGTTACACCATAATTCACTGCGCTGGTGTCGATGTTGGAC
	CCCTGAAAGACGCGCAGACTGCGGCCGCGCATAACGTCA AGTTTGTCTTCGAC	AATTTACTCGAGTCAGAAGAGCCCGCAGTCTTTG
GB1-11mer	AATTTAGGATCCATCAAAGAAAACCTGAAAGACTGCGGT	TAAATTAAGCTTAGAACAGACCGCAGTCTTTCAGGTTTTC

* primer paired with PCR product

Supplementary Note 1. Rossetta scripts and commands for FloppyTail modeling

Rosetta flag file for Ric8a 1-452 model

```
# modified from the script available through Rosetta distribution and
written by Steven Lewis.
-s ../Bt452_no_Helix.pdb
-ex1
-ex2
-use_input_sc
-packing:repack_only
-run:min_type dfpmin_armijo_nonmonotone
-FloppyTail:flexible_start_resnum 423
-FloppyTail:flexible_stop_resnum 452
-FloppyTail:flexible_chain A
-FloppyTail:short_tail:short_tail_off 0
-FloppyTail:short_tail:short_tail_fraction 1.0
-constraints:cst_file ../constraints
-constraints:cst_weight 10
-constraints:cst_fa_file ../constraints
-constraints:cst_fa_weight 10
-FloppyTail:shear_on .33333333333333333333
-FloppyTail:publication false
-FloppyTail:perturb_temp  0.8
-FloppyTail:refine_temp  0.8
-FloppyTail:refine_repack_cycles 100
-FloppyTail:perturb_cycles 5000
-FloppyTail:refine_cycles 3000
-nstruct 5000
```

Constrain file

```
AtomPair CA 408A CA 449A FLAT_HARMONIC 15 1 15
AtomPair CA 352A CA 449A FLAT_HARMONIC 15 1 15
```

Rosetta flag file for Ric8a 1-492 MiniG model

```
# modified from the script available through Rosetta distribution and
written by Steven Lewis.
-s ../Ric8a_miniGi.pdb
-ex1
-ex2
-use_input_sc
-packing:repack_only
-run:min_type dfpmin_armijo_nonmonotone
-FloppyTail:flexible_start_resnum 448
-FloppyTail:flexible_stop_resnum 492
-FloppyTail:flexible_chain B
-FloppyTail:short_tail:short_tail_off 0
-FloppyTail:short_tail:short_tail_fraction 1.0
-constraints:cst_file ../constraints
-constraints:cst_weight 10
-constraints:cst_fa_file ../constraints
-constraints:cst_fa_weight 10
-FloppyTail:shear_on .33333333333333333333
-FloppyTail:publication false
-FloppyTail:perturb_temp  0.8
-FloppyTail:refine_temp  0.8
-FloppyTail:refine_repack_cycles 30
-FloppyTail:perturb_cycles 12000
-FloppyTail:refine_cycles 3000
-nstruct 5000
```

Constraints file

```
AtomPair CA 121A CA 488B FLAT_HARMONIC 15 1 15
AtomPair CA  22A CA 462B FLAT_HARMONIC 15 1 15
```

Code used for starting multiple independent calculations

```
for ((i=0; i<$1; i++))
do
  mkdir run$i
  cd run$i
  cp ../flag_file .
  pwd
  FloppyTail.linuxgccrelease @flag_file > run$i.log &
  cd ..
done
```

Code used for pooling results of multiple independent calculations

```
#!/bin/sh
find run* -name "*.pdb" | awk '{print "cp " $1 " final/" NR
".pdb"}'
```

Command used for scoring the pooled models

```
score_jd2.linuxgccrelease –s *.pdb
```
