# Supplementary material for: Structural underpinnings of Ric8A function as a G-protein α-subunit chaperone and guanine-nucleotide exchange factor
Source: Nat Commun. 2019 Jul 12;10:3084. doi: 10.1038/s41467-019-11088-x (PMC6625990; doi:10.1038/s41467-019-11088-x)
Supplement: Supplementary file 3 — Description of Additional Supplementary Files [file 41467_2019_11088_MOESM3_ESM.docx]

**Description of Supplementary Files**

**File Name:** **Supplementary Data 1**

**Description:** Amino Acid Conservation Scores from the ConSurf analysis of 250 Ric8 homologues

**File Name:** **Supplementary Data 2**

**Description:** Intramolecular DSS-crosslinked peptides of apo Ric8A1-492

**File Name:** **Supplementary Data 3**

**Description:** Intermolecular DSS-crosslinked peptides of the Ric8A1-492/miniGαi complex

**File Name:** **Supplementary Data 4**

**Description:** Intermolecular DSS-crosslinked peptides of the Ric8A1-492/Gαt complex

**File Name:** **Supplementary Data 5**

**Description:** Coordinates file for the model of Ric8A1-452

**File Name:** **Supplementary Data 6**

**Description:** Coordinates file for the model of apo Ric8A1-492

**File Name:** **Supplementary Data 7**

**Description:** Coordinates file for the model of Ric8A/minGi complex

**File Name:** **Supplementary Data 8**

**Description:** Coordinates file for the model Ric8A/Gαi complex
