# Supplementary material for: Structural underpinnings of Ric8A function as a G-protein α-subunit chaperone and guanine-nucleotide exchange factor
Source: Nat Commun. 2019 Jul 12;10:3084. doi: 10.1038/s41467-019-11088-x (PMC6625990; doi:10.1038/s41467-019-11088-x)
Supplement: Supplementary file 4 — Supplementary Data 1 [file 41467_2019_11088_MOESM4_ESM.pdf]

Supplementary Data 1. Amino Acid Conservation Scores from the ConSurf analysis of 250 Ric8 homologues. The sequences for the analysis with maximal identity of 97% and minimal identity of 30% were collected from the UniProt database. Residues involved in the interface with $G\alpha_t$333-350 are underlined.

Amino Acid Conservation Scores
=======================================

- POS: The position of the AA in the SEQRES derived sequence.
- SEQ: The SEQRES derived sequence in one letter code.
- 3LATOM: The ATOM derived sequence in three letter code, including the AA's positions as they appear in the PDB file and the chain identifier.
- SCORE: The normalized conservation scores.
- COLOR: The color scale representing the conservation scores (9 - conserved, 1 - variable).
- CONFIDENCE INTERVAL: When using the bayesian method for calculating rates, a confidence interval is assigned to each of the inferred evolutionary conservation scores.
- CONFIDENCE INTERVAL COLORS: When using the bayesian method for calculating rates. The color scale representing the lower and upper bounds of the confidence interval.
- MSA DATA: The number of aligned sequences having an amino acid (non-gapped) from the overall number of sequences at each position.
- RESIDUE VARIETY: The residues variety at each position of the multiple sequence alignment.

POS	SEQ	3LATOM	SCORE (normalized)	COLOR	CONFIDENCE INTERVAL	CONFIDENCE INTERVAL COLORS	MSA DATA	RESIDUE VARIETY
1	M	MET1:A	-0.701	7	-1.016,-0.505	8,7	43/250	M,E,L
2	E	GLU2:A	0.107	5	-0.427, 0.393	6,4	48/250	G,L,E,D
3	P	PRO3:A	0.870	2*	0.084, 1.187	5,1	53/250	V,A,F,P,G,L,E
4	R	ARG4:A	2.464	1	1.187, 3.096	1,1	66/250	D,G,A,I,K,Q,N,S,H,V,R
5	A	ALA5:A	0.962	2	0.393, 1.187	4,1	73/250	G,T,A,S,L,K,V,R,H
6	V	VAL6:A	0.266	4	-0.153, 0.596	6,3	89/250	M,V,A,I,T,E,L
7	A	ALA7:A	-0.077	5	-0.427, 0.084	6,5	90/250	E,L,F,I,A,V
8	D	ASP8:A	0.404	4	-0.043, 0.596	5,3	95/250	T,G,D,M,A,Q,K,L,S,E,V
9	A	ALA9:A	1.320	1	0.596, 1.712	3,1	104/250	C,V,H,E,N,S,K,Q,A,I,D,T
10	L	LEU10:A	0.091	5	-0.253, 0.227	6,4	123/250	G,M,F,I,E,S,L,V
11	E	GLU11:A	0.589	3	0.227, 0.848	4,2	127/250	K,Q,S,L,E,H,V,R,C,T,G,D,A
12	T	THR12:A	1.118	1	0.596, 1.187	3,1	129/250	R,V,E,S,L,N,K,Q,A,I,G,T
13	G	GLY13:A	-0.187	6	-0.505,-0.043	7,5	133/250	A,G,D,T,R,V,C,N,S,E,K
14	E	GLU14:A	0.901	2	0.393, 1.187	4,1	135/250	E,S,N,K,Q,A,D,G,T
15	E	GLU15:A	0.489	3	0.084, 0.596	5,3	135/250	G,T,I,N,L,E,K,Q,V,R,P,W,D,A,S,Y
16	D	ASP16:A	0.708	3	0.227, 0.848	4,2	138/250	P,R,H,E,S,N,K,Q,A,D,G,T
17	V	VAL17:A	0.979	2	0.393, 1.187	4,1	138/250	D,G,T,I,A,E,S,L,K,R,V,H
18	V	VAL18:A	0.363	4	0.084, 0.596	5,3	140/250	C,V,L,F,A,I,D,T
19	M	MET19:A	1.229	1	0.596, 1.712	3,1	143/250	H,P,R,V,Q,K,E,L,N,M,I,T,G,C,S,A,D
20	E	GLU20:A	1.765	1	1.187, 1.712	1,1	146/250	R,V,P,S,N,L,E,Q,K,A,I,M,D,T
21	A	ALA21:A	-0.014	5	-0.253, 0.084	6,5	148/250	T,G,M,A,I,Q,L,S,E,H,R,V,P
22	L	LEU22:A	-1.151	9	-1.252,-1.101	9,9	151/250	V,I,L
23	R	ARG23:A	2.399	1	1.187, 3.096	1,1	154/250	E,N,L,Q,K,V,R,H,G,T,F,I,Y,S,D,A
24	A	ALA24:A	3.004	1	1.712, 3.096	1,1	154/250	E,N,L,K,Q,W,R,V,H,G,T,I,S,C,D,A
25	Y	TYR25:A	-0.292	6	-0.576,-0.153	7,6	159/250	Y,L,F,A,I,V
26	N	ASN26:A	-0.530	7	-0.706,-0.427	7,6	162/250	L,S,N,Q,T,V,I

27	R	ARG27:A	1.647	1	0.848, 1.712	2,1	164/250	Y,S,C,D,A,K,Q,E,L,N,H,P,R,V,T,G,I
28	E	GLU28:A	0.908	2	0.393, 1.187	4,1	165/250	H,R,Q,K,N,S,L,E,A,T,D
29	N	ASN29:A	-0.592	7	-0.764,-0.505	8,7	171/250	H,Y,N,S,Q,K,F,A,M
30	S	SER30:A	0.077	5	-0.153, 0.227	6,4	171/250	A,F,G,D,T,R,C,N,L,S,E,Q,K
31	Q	GLN31:A	0.133	5	-0.153, 0.227	6,4	171/250	G,D,T,A,N,S,L,E,K,Q,R,H
32	S	SER32:A	0.112	5	-0.153, 0.227	6,4	172/250	T,I,A,M,L,S,K,R,V,P,C
33	F	PHE33:A	-1.019	8	-1.141,-0.922	9,8	173/250	I,R,F,S,L,T
34	T	THR34:A	0.385	4	0.084, 0.596	5,3	175/250	H,R,V,Q,K,E,N,L,M,F,I,T,G,S,A,D
35	F	PHE35:A	-1.118	9	-1.252,-1.059	9,9	175/250	Y,L,F,I,A
36	D	ASP36:A	1.397	1	0.848, 1.712	2,1	175/250	T,G,D,M,I,A,K,Q,S,L,N,E,H,V,P
37	D	ASP37:A	1.235	1	0.596, 1.712	3,1	176/250	F,T,W,P,R,H,E,N,L,Q,K,A,D,C,Y,S
38	A	ALA38:A	0.712	3	0.227, 0.848	4,2	176/250	K,E,L,N,H,V,T,G,M,F,I,Y,S,C,D,A
39	Q	GLN39:A	-0.056	5	-0.345, 0.084	6,5	177/250	P,H,E,N,L,S,Q,A,I,D,G,T
40	Q	GLN40:A	1.519	1	0.848, 1.712	2,1	178/250	S,C,D,A,K,Q,L,N,E,H,R,V,P,W,T,G,M,I,F
41	E	GLU41:A	0.668	3	0.227, 0.848	4,2	180/250	X,A,F,T,G,D,R,C,K,Q,L,N,S,E
42	D	ASP42:A	0.378	4	0.084, 0.596	5,3	181/250	A,D,G,T,C,V,R,E,S,N,L,Q,K
43	R	ARG43:A	-0.402	6	-0.576,-0.253	7,6	182/250	T,F,I,Q,K,N,L,S,V,R
44	K	LYS44:A	0.497	3	0.084, 0.596	5,3	184/250	R,V,H,Y,E,S,L,Q,K,F,I,A,M,T
45	R	ARG45:A	0.549	3	0.227, 0.848	4,2	190/250	D,A,Y,S,C,G,T,I,E,L,N,Q,K,V,R,H
46	L	LEU46:A	-0.615	7	-0.820,-0.505	8,7	194/250	C,P,R,V,L,F,A,I,M
47	A	ALA47:A	-0.415	6	-0.643,-0.253	7,6	204/250	A,M,G,W,C,R,V,E,Y,L,S
48	K	LYS48:A	1.368	1	0.848, 1.712	2,1	204/250	G,T,I,M,E,N,L,K,Q,V,R,H,D,A,Y,S
49	L	LEU49:A	0.403	4	0.084, 0.596	5,3	204/250	T,D,G,M,A,I,Q,K,E,N,S,L,R,V
50	L	LEU50:A	0.038	5	-0.253, 0.227	6,4	204/250	F,A,I,M,T,R,V,L
51	V	VAL51:A	0.108	5	-0.153, 0.227	6,4	204/250	E,S,L,V,G,T,F,A,I,M
52	S	SER52:A	1.700	1	1.187, 1.712	1,1	204/250	D,A,Y,S,C,G,T,F,I,M,E,N,L,K,Q,P,R,H
53	V	VAL53:A	0.910	2	0.393, 1.187	4,1	204/250	H,C,W,R,V,K,Y,L,M,F,I,A,T
54	L	LEU54:A	-0.665	7	-0.872,-0.576	8,7	204/250	F,A,I,M,V,E,S,L
55	E	GLU55:A	1.218	1	0.596, 1.712	3,1	204/250	D,G,T,A,E,N,L,S,Q,K,V,R
56	Q	GLN56:A	0.925	2	0.393, 1.187	4,1	204/250	S,L,N,E,K,Q,V,R,P,C,G,D,T,A
57	G	GLY57:A	1.383	1	0.848, 1.712	2,1	204/250	C,Y,S,A,D,P,R,V,H,E,N,Q,K,F,I,G,T
58	L	LEU58:A	1.396	1	0.848, 1.712	2,1	205/250	P,R,V,E,L,N,S,M,F,A,I,T,D,G
59	P	PRO59:A	1.102	1	0.596, 1.187	3,1	205/250	T,G,M,F,K,Q,L,N,E,H,R,P,D,A,S,Y
60	P	PRO60:A	0.931	2	0.393, 1.187	4,1	204/250	I,T,G,H,R,V,W,P,K,Q,L,N,E,A,C,S
61	S	SER61:A	1.110	1	0.596, 1.187	3,1	195/250	A,D,C,S,I,F,G,T,R,P,H,N,L,E,K,Q
62	R	ARG62:A	1.324	1	0.848, 1.712	2,1	204/250	T,M,F,I,Q,E,N,L,H,V,R,D,A,Y,S,C
63	R	ARG63:A	-0.190	6	-0.427,-0.043	6,5	206/250	M,I,A,G,H,R,C,Q,K,L,S,Y
64	V	VAL64:A	0.934	2	0.393, 1.187	4,1	206/250	T,M,F,I,A,Q,K,Y,E,L,S,H,P,V,R
65	I	ILE65:A	1.374	1	0.848, 1.712	2,1	206/250	S,Y,A,D,H,R,V,P,K,Q,N,L,E,M,I,T,G
66	W	TRP66:A	-0.197	6	-0.427,-0.043	6,5	208/250	D,T,F,A,I,Y,L,S,W,C,V

67	L	LEU67:A	-1.194	9	-1.286,-1.141	9,9	210/250	I,F,L,D
68	Q	GLN68:A	-0.195	6	-0.427,-0.043	6,5	212/250	E,N,S,Q,K,C,V,R,H,T,A,I,M
69	S	SER69:A	-0.501	7	-0.706,-0.427	7,6	212/250	A,I,V,C,L,S,T
70	I	ILE70:A	-0.280	6	-0.505,-0.153	7,6	213/250	I,V,F,M,L,E
71	R	ARG71:A	-1.082	9	-1.179,-1.016	9,8	213/250	K,L,R,I
72	I	ILE72:A	-0.961	8	-1.059,-0.922	9,8	214/250	F,V,I,M,L,Q
73	L	LEU73:A	-0.921	8	-1.059,-0.820	9,8	214/250	M,F,I,V,L
74	S	SER74:A	-1.086	9	-1.179,-1.059	9,9	214/250	S,T,C,A,I,H
75	R	ARG75:A	-1.339	9	-1.368,-1.320	9,9	214/250	R
76	D	ASP76:A	-1.095	9	-1.179,-1.059	9,9	214/250	D,E,S,A
77	R	ARG77:A	-0.786	8	-0.922,-0.706	8,7	214/250	G,H,R,C,P,Q,K,N,E
78	S	SER78:A	1.056	1	0.596, 1.187	3,1	214/250	T,G,M,F,K,Q,N,L,E,H,R,V,D,A,S,Y,C
79	C	CYS79:A	0.202	4	-0.043, 0.393	5,4	214/250	H,C,V,K,E,Y,N,L,S,F,I,A,T,D,G
80	L	LEU80:A	-0.461	7	-0.643,-0.345	7,6	214/250	Q,S,L,V,C,W,T,M,A,I
81	D	ASP81:A	0.965	2	0.596, 1.187	3,1	214/250	Q,K,E,S,L,N,P,C,V,D,G,M,A
82	S	SER82:A	1.319	1	0.848, 1.712	2,1	214/250	S,A,D,H,P,V,R,Q,K,E,N,L,M,F,T,G
83	F	PHE83:A	-0.310	6	-0.505,-0.153	7,6	214/250	H,V,C,L,N,S,M,I,A,F,T
84	T	THR84:A	0.333	4	0.084, 0.393	5,4	214/250	I,A,F,G,T,V,C,L,N,S,E,Y
85	S	SER85:A	-0.238	6	-0.427,-0.153	6,6	214/250	I,A,M,D,G,T,C,R,E,N,S,K,Q
86	R	ARG86:A	0.968	2	0.596, 1.187	3,1	214/250	S,C,D,A,E,N,L,K,Q,W,P,V,R,H,G,T,M
87	R	ARG87:A	1.440	1	0.848, 1.712	2,1	214/250	T,D,G,F,A,I,Q,K,E,N,S,H,P,V,R
88	S	SER88:A	0.314	4	-0.043, 0.393	5,4	214/250	Y,S,C,D,A,K,Q,E,N,L,H,W,R,V,T,G,M,F,I
89	L	LEU89:A	0.608	3	0.227, 0.848	4,2	214/250	I,F,M,D,T,V,L,S,Y
90	Q	GLN90:A	0.835	2	0.393, 1.187	4,1	214/250	R,V,H,S,N,L,E,Q,K,I,A,F,G,D,T
91	A	ALA91:A	0.208	4	-0.043, 0.393	5,4	214/250	G,T,I,A,M,L,Y,K,V,C,H
92	L	LEU92:A	-0.946	8	-1.101,-0.872	9,8	214/250	L,V,I,A,M
93	A	ALA93:A	0.135	5	-0.153, 0.227	6,4	214/250	V,C,L,S,E,Q,K,A,I,F,M,G,T
94	C	CYS94:A	1.163	1	0.596, 1.187	3,1	214/250	D,A,Y,S,C,G,T,M,E,N,L,Q,K,R,V,H
95	Y	TYR95:A	0.897	2	0.393, 1.187	4,1	214/250	C,W,R,V,H,Y,E,L,N,K,F,I,A,M
96	A	ALA96:A	-0.961	8	-1.059,-0.922	9,8	214/250	G,S,T,A,I,R
97	G	GLY97:A	0.500	3	0.084, 0.596	5,3	213/250	H,R,P,C,K,N,S,L,E,M,A,F,G,D
98	I	ILE98:A	-0.373	6	-0.576,-0.253	7,6	213/250	R,V,L,E,M,I,F,T
99	S	SER99:A	2.901	1	1.712, 3.096	1,1	212/250	C,S,Y,A,D,H,V,R,P,Q,K,L,N,E,F,T,G
100	A	ALA100:A	3.096	1	1.712, 3.096	1,1	212/250	Q,K,E,L,N,H,P,V,R,T,G,M,F,I,Y,S,C,D,A
101	S	SER101:A	2.911	1	1.712, 3.096	1,1	209/250	D,A,Y,S,C,G,T,F,I,M,E,N,L,Q,K,P,W,V,R,H
102	Q	GLN102:A	3.048	1	1.712, 3.096	1,1	124/250	E,L,S,K,Q,C,P,V,D,G,T,F,A,M
103	G	GLY103:A	3.089	1	1.712, 3.096	1,1	159/250	N,L,E,K,Q,V,R,P,H,G,T,I,M,S,C,D,A
104	S	SER104:A	3.096	1	1.712, 3.096	1,1	188/250	P,R,V,H,E,L,N,Q,K,F,I,M,G,T,Y,S,A,D
105	V	VAL105:A	3.095	1	1.712, 3.096	1,1	201/250	H,R,V,W,P,Q,K,N,L,E,M,I,F,T,G,C,S,A,D
106	P	PRO106:A	3.092	1	1.712, 3.096	1,1	209/250	A,D,C,S,Y,I,F,G,T,R,V,W,P,H,L,N,E,Q,K

107	E	GLU107:A	2.022	1	1.187, 3.096	1,1	216/250	S,C,D,A,E,L,N,K,Q,P,W,V,R,H,G,T,F,I
108	P	PRO108:A	3.095	1	1.712, 3.096	1,1	217/250	M,I,F,T,G,H,R,V,P,Q,K,L,N,E,A,D,C,S,Y
109	L	LEU109:A	2.636	1	1.187, 3.096	1,1	216/250	H,P,W,V,K,Q,E,N,L,M,F,I,T,G,C,Y,S,A,D
110	N	ASN110:A	1.152	1	0.596, 1.187	3,1	219/250	E,L,N,K,Q,P,V,R,H,G,T,I,Y,S,C,D,A
111	M	MET111:A	2.436	1	1.187, 3.096	1,1	222/250	A,D,Y,S,F,I,M,G,T,P,V,H,E,L,K,Q
112	D	ASP112:A	1.528	1	0.848, 1.712	2,1	224/250	M,I,T,G,H,P,R,V,K,Q,E,N,L,A,D,C,S
113	V	VAL113:A	-0.114	5	-0.345,-0.043	6,5	226/250	C,V,R,E,S,N,L,Q,K,F,A,I,D,G,T
114	V	VAL114:A	0.179	4	-0.043, 0.393	5,4	227/250	Q,K,N,S,L,Y,E,R,V,C,T,M,A,I
115	L	LEU115:A	0.092	5	-0.153, 0.227	6,4	230/250	G,T,A,I,F,M,N,S,L,E,K,Q,V
116	E	GLU116:A	-1.157	9	-1.252,-1.101	9,9	240/250	A,N,G,L,Y,E
117	S	SER117:A	-0.737	7	-0.872,-0.643	8,7	240/250	G,S,V,R,A
118	L	LEU118:A	-0.242	6	-0.505,-0.153	7,6	240/250	E,L,Q,K,C,V,R,M
119	K	LYS119:A	-1.279	9	-1.352,-1.252	9,9	240/250	H,S,K
120	C	CYS120:A	-0.596	7	-0.764,-0.505	8,7	240/250	V,C,S,L,A,I,T,G
121	L	LEU121:A	-0.943	8	-1.059,-0.872	9,8	240/250	F,C,I,V,S,L
122	C	CYS122:A	-0.897	8	-1.059,-0.820	9,8	240/250	S,L,Y,W,C,F
123	N	ASN123:A	-1.330	9	-1.368,-1.320	9,9	240/250	S,N
124	L	LEU124:A	-0.195	6	-0.427,-0.043	6,5	240/250	M,V,A,I,L
125	V	VAL125:A	-0.459	7	-0.643,-0.345	7,6	240/250	C,V,L,S,F,I,A,M,T
126	L	LEU126:A	-0.111	5	-0.345, 0.084	6,5	240/250	F,L,Y
127	S	SER127:A	-0.830	8	-0.970,-0.764	8,8	240/250	I,T,H,S,N,L,K,Q
128	S	SER128:A	-1.008	8	-1.101,-0.970	9,8	240/250	T,G,F,N,S,L,H,C
129	P	PRO129:A	2.173	1	1.187, 3.096	1,1	240/250	H,P,V,R,K,Q,E,N,L,M,I,T,G,C,S,A,D
130	V	VAL130:A	1.627	1	1.187, 1.712	1,1	240/250	D,A,S,Y,G,T,I,M,N,L,E,K,Q,R,V,W,P,H
131	A	ALA131:A	-0.815	8	-0.970,-0.764	8,8	240/250	C,R,V,S,L,I,A,M,G
132	Q	GLN132:A	-0.736	7	-0.872,-0.643	8,7	240/250	A,I,R,V,Q,K,L,N,S
133	A	ALA133:A	1.019	2	0.596, 1.187	3,1	240/250	H,R,V,K,Q,L,N,S,E,M,A,I,T,G,D
134	L	LEU134:A	1.060	1	0.596, 1.187	3,1	239/250	L,E,K,Q,R,V,H,T,I,F,M,S,Y,C,D,A
135	A	ALA135:A	0.136	5	-0.153, 0.227	6,4	240/250	D,G,T,F,I,A,M,L,S,Q,K,C,V
136	A	ALA136:A	0.056	5	-0.153, 0.227	6,4	240/250	P,C,R,V,Q,K,E,N,L,S,I,A,T,G
137	E	GLU137:A	1.167	1	0.596, 1.187	3,1	240/250	V,R,P,C,H,N,L,S,E,K,Q,A,G,D,T
138	A	ALA138:A	-0.380	6	-0.576,-0.253	7,6	239/250	M,F,I,T,G,H,V,K,Q,E,N,L,A,C,Y,S
139	G	GLY139:A	0.574	3	0.227, 0.848	4,2	239/250	Q,K,N,S,E,H,R,C,P,W,T,G,D,A
140	L	LEU140:A	0.083	5	-0.153, 0.227	6,4	240/250	L,S,V,W,C,G,T,A,I,F
141	V	VAL141:A	-0.019	5	-0.253, 0.084	6,5	240/250	T,D,M,I,A,L,E,V,C
142	V	VAL142:A	0.764	2	0.393, 0.848	4,2	240/250	F,T,G,H,R,V,P,K,Q,N,L,E,A,D,C,S
143	R	ARG143:A	0.394	4	0.084, 0.596	5,3	241/250	A,Y,S,C,T,G,M,K,Q,E,N,L,H,P,V,R
144	L	LEU144:A	-0.484	7	-0.643,-0.345	7,6	241/250	Y,L,S,I,A,V
145	A	ALA145:A	0.644	3	0.227, 0.848	4,2	241/250	A,I,F,M,T,V,W,C,H,L,S,K
146	E	GLU146:A	1.071	1	0.596, 1.187	3,1	241/250	H,P,R,V,K,Q,E,S,L,N,M,A,T,D,G

147	R	ARG147:A	-0.639	7	-0.820,-0.576	8,7	241/250	R,C,H,N,L,S,K,Q,T
148	V	VAL148:A	-0.294	6	-0.505,-0.153	7,6	241/250	C,V,I,M,S,L,T
149	G	GLY149:A	0.842	2	0.393, 1.187	4,1	234/250	N,L,S,E,K,Q,V,R,C,H,G,D,A,F,M
150	L	LEU150:A	0.619	3	0.227, 0.848	4,2	234/250	P,W,V,R,H,E,L,N,Q,K,F,I,M,G,T,C,S,A,D
151	C	CYS151:A	1.162	1	0.596, 1.187	3,1	239/250	T,G,I,F,Q,L,N,E,H,V,W,P,D,A,S,Y,C
152	R	ARG152:A	0.790	2	0.393, 0.848	4,2	238/250	S,Y,A,D,R,V,P,H,N,L,E,Q,K,I,F,M,G,T
153	Q	GLN153:A	0.815	2	0.393, 0.848	4,2	239/250	A,T,D,G,H,P,W,V,R,K,Q,E,S,N
154	S	SER154:A	2.452	1	1.187, 3.096	1,1	205/250	D,A,Y,S,C,T,G,M,I,Q,K,E,L,N,H,W,P,R,V
155	S	SER155:A	1.339	1	0.848, 1.712	2,1	216/250	Q,K,L,N,S,E,H,R,V,P,T,G,D,M,A
156	F	PHE156:A	1.542	1	0.848, 1.712	2,1	239/250	A,D,C,S,Y,I,F,M,G,T,V,R,P,W,H,L,N
157	P	PRO157:A	1.258	1	0.848, 1.712	2,1	240/250	T,G,I,A,K,Q,N,L,S,E,H,V,R,P,C
158	H	HIS158:A	0.691	3	0.393, 0.848	4,2	240/250	Q,K,E,N,L,H,W,P,T,G,M,F,I,Y,S,C,D
159	D	ASP159:A	0.225	4	-0.043, 0.393	5,4	241/250	A,F,D,T,V,N,S,E,Y,K,Q
160	V	VAL160:A	-0.347	6	-0.576,-0.253	7,6	239/250	G,T,A,I,M,N,S,L,V,C,H
161	Q	GLN161:A	-0.239	6	-0.427,-0.153	6,6	239/250	S,N,L,E,Q,K,R,W,H,G,T,A,I,F,M
162	F	PHE162:A	0.140	5	-0.153, 0.393	6,4	239/250	I,F,T,H,V,C,Q,L,Y
163	F	PHE163:A	-0.852	8	-1.016,-0.764	8,8	239/250	T,Y,L,F,I
164	D	ASP164:A	-0.818	8	-0.970,-0.764	8,8	239/250	M,I,A,T,D,V,K,S,N,E,Y
165	L	LEU165:A	-0.575	7	-0.764,-0.505	8,7	239/250	L,T,I,A,V,M
166	R	ARG166:A	-1.148	9	-1.252,-1.101	9,9	239/250	R,Q,K
167	L	LEU167:A	-0.556	7	-0.764,-0.427	8,6	238/250	L,Y,T,V,I,F,M
168	L	LEU168:A	-0.296	6	-0.505,-0.153	7,6	239/250	F,I,A,M,T,C,V,L
169	F	PHE169:A	-1.210	9	-1.286,-1.179	9,9	237/250	F,L,S
170	L	LEU170:A	-0.961	8	-1.101,-0.872	9,8	238/250	A,V,I,L,G,Y
171	L	LEU171:A	-0.139	5	-0.345,-0.043	6,5	237/250	M,A,I,F,S,L,V,C
172	T	THR172:A	-1.232	9	-1.286,-1.216	9,9	237/250	S,T,R
173	A	ALA173:A	-1.166	9	-1.252,-1.141	9,9	239/250	A,I,P,C,T,G,L
174	L	LEU174:A	-0.817	8	-0.970,-0.706	8,7	241/250	V,Y,S,L,Q,F,I,A,M
175	R	ARG175:A	-0.203	6	-0.427,-0.043	6,5	241/250	T,D,I,A,Q,K,N,L,S,E,H,R,C,P
176	T	THR176:A	0.741	3	0.393, 0.848	4,2	241/250	L,E,Q,K,V,R,P,H,G,T,I,M,S,C,D,A
177	D	ASP177:A	-0.061	5	-0.345, 0.084	6,5	242/250	A,T,G,D,H,V,C,Q,K,N,S,E
178	V	VAL178:A	0.011	5	-0.253, 0.084	6,5	242/250	V,C,L,S,N,I,A,M,G,D,T
179	R	ARG179:A	-1.033	8	-1.141,-0.970	9,8	240/250	A,T,G,R,K,Q,S,N
180	Q	GLN180:A	0.930	2	0.596, 1.187	3,1	239/250	D,A,S,T,G,M,I,K,Q,L,N,E,H,V,R,P
181	Q	GLN181:A	0.319	4	-0.043, 0.393	5,4	240/250	I,A,F,M,G,D,T,R,V,P,L,S,E,K,Q
182	L	LEU182:A	-0.346	6	-0.576,-0.253	7,6	240/250	I,A,V,F,M,L
183	F	PHE183:A	0.522	3	0.227, 0.596	4,3	240/250	M,A,I,F,T,H,V,R,C,Q,K,S,N,L,Y
184	Q	GLN184:A	1.012	2	0.596, 1.187	3,1	242/250	Q,K,Y,E,N,S,H,R,V,T,D,F,I
185	E	GLU185:A	-0.373	6	-0.576,-0.253	7,6	241/250	C,Q,K,E,S,N,I,A,T,D
186	L	LEU186:A	0.035	5	-0.253, 0.227	6,4	212/250	A,S,Y,C,T,G,M,I,F,Q,L,N,H,V,R,W

187	Q	GLN187:A	-0.008	5	-0.253, 0.084	6,5	240/250	H,C,R,Q,K,Y,N,S,F,A,D,G
188	G	GLY188:A	-0.912	8	-1.059,-0.820	9,8	241/250	E,G,V,A,I
189	V	VAL189:A	-0.293	6	-0.505,-0.153	7,6	244/250	P,V,R,L,S,F,A,I,M,D
190	R	ARG190:A	1.005	2	0.596, 1.187	3,1	244/250	M,F,I,T,G,H,P,V,R,K,Q,E,L,N,A,D,C,S
191	L	LEU191:A	0.291	4	-0.043, 0.393	5,4	245/250	H,R,V,P,Q,N,L,E,M,I,F,T,G,C,S,Y,D
192	L	LEU192:A	-0.963	8	-1.101,-0.872	9,8	245/250	L,I,F,M
193	T	THR193:A	-0.113	5	-0.345,-0.043	6,5	244/250	M,A,I,T,G,V,C,K,L,N,S,Y
194	R	ARG194:A	0.663	3	0.227, 0.848	4,2	244/250	K,Q,E,L,N,S,H,P,R,V,T,D,G,I,A
195	A	ALA195:A	0.142	5	-0.153, 0.227	6,4	243/250	H,V,C,W,Q,K,L,N,Y,M,I,A,F,T
196	L	LEU196:A	-0.910	8	-1.059,-0.820	9,8	243/250	V,A,I,M,L
197	E	GLU197:A	-0.351	6	-0.576,-0.253	7,6	243/250	R,L,N,E,Y,K,Q,A,G,D,T
198	L	LEU198:A	0.870	2	0.393, 1.187	4,1	244/250	D,A,S,Y,C,G,T,I,F,M,N,L,E,K,Q,V,R,W,H
199	T	THR199:A	0.105	5	-0.153, 0.227	6,4	243/250	V,R,C,L,S,E,K,Q,I,A,F,M,T
200	L	LEU200:A	0.231	4	-0.043, 0.393	5,4	243/250	H,C,V,K,Q,Y,S,L,M,F,I,A,T,G
201	G	GLY201:A	1.114	1	0.596, 1.187	3,1	240/250	S,C,D,A,E,L,N,K,Q,P,V,R,H,G,T,I
202	M	MET202:A	0.740	3	0.393, 0.848	4,2	232/250	R,V,P,L,N,E,K,Q,I,M,G,T,C,S,Y,A,D
203	T	THR203:A	1.600	1	1.187, 1.712	1,1	233/250	D,A,Y,S,C,T,G,F,I,Q,K,E,L,N,H,P,V,R
204	E	GLU204:A	1.721	1	0.848, 1.712	2,1	181/250	V,C,P,Q,K,L,N,S,E,M,A,T,G,D
205	G	GLY205:A	3.074	1	1.712, 3.096	1,1	189/250	H,P,R,V,Q,K,E,L,N,S,I,A,T,D,G
206	E	GLU206:A	3.022	1	1.712, 3.096	1,1	193/250	S,D,A,Q,K,L,N,E,H,R,V,P,T,G,M,I
207	R	ARG207:A	2.420	1	1.187, 3.096	1,1	194/250	D,A,Y,S,G,T,I,M,L,N,K,Q,P,V,R,H
208	H	HIS208:A	1.229	1	0.596, 1.712	3,1	199/250	H,V,P,Q,K,N,L,E,M,I,F,T,G,C,S,Y,A,D
209	P	PRO209:A	0.411	4*	-0.345, 0.848	6,2	39/250	P,G,L
210	E	GLU210:A	2.598	1	1.187, 3.096	1,1	39/250	K,D,E,R,V,A
211	L	LEU211:A	1.419	1	0.848, 1.712	2,1	221/250	A,D,S,Y,M,I,F,T,G,V,R,P,Q,K,L,E
212	L	LEU212:A	0.296	4	-0.043, 0.393	5,4	244/250	V,K,Q,L,N,E,Y,M,A,I,F,T
213	P	PRO213:A	0.243	4	-0.043, 0.393	5,4	245/250	Y,S,C,D,Q,K,E,L,N,H,P,V,R,T,G,F,I
214	P	PRO214:A	0.872	2	0.393, 1.187	4,1	246/250	E,N,S,L,K,Q,C,P,V,R,D,T,A,M
215	Q	GLN215:A	0.915	2	0.393, 1.187	4,1	247/250	A,F,M,G,D,T,R,P,H,L,N,S,E,K,Q
216	E	GLU216:A	0.704	3	0.393, 0.848	4,2	247/250	V,R,H,E,Y,L,S,N,Q,K,A,M,D,G,T
217	T	THR217:A	-0.180	6	-0.427,-0.043	6,5	247/250	T,G,M,F,I,A,Q,E,N,S,L,H,R,V
218	E	GLU218:A	-0.253	6	-0.427,-0.153	6,6	247/250	A,I,D,T,V,H,E,L,N,Q,K
219	R	ARG219:A	-0.025	5	-0.253, 0.084	6,5	250/250	F,I,T,D,H,C,V,R,Q,K,Y,L
220	A	ALA220:A	0.244	4	-0.043, 0.393	5,4	250/250	M,I,A,T,G,C,V,L,S
221	M	MET221:A	-0.044	5	-0.253, 0.084	6,5	250/250	L,N,S,Q,C,V,D,G,T,A,I,M
222	E	GLU222:A	-1.232	9	-1.320,-1.216	9,9	250/250	A,W,G,D,E
223	I	ILE223:A	-0.147	5	-0.345,-0.043	6,5	250/250	T,M,A,I,S,L,E,V,C
224	L	LEU224:A	-1.182	9	-1.286,-1.141	9,9	250/250	S,L,F,M
225	K	LYS225:A	-1.285	9	-1.352,-1.252	9,9	250/250	Q,K,A
226	V	VAL226:A	-0.530	7	-0.706,-0.427	7,6	250/250	F,A,I,V,L,T

227	L	LEU227:A	-0.902	8	-1.059,-0.820	9,8	250/250	M,F,I,Q,L
228	F	PHE228:A	-1.133	9	-1.252,-1.059	9,9	250/250	Y,F
229	N	ASN229:A	-1.169	9	-1.252,-1.141	9,9	250/250	S,N,T,H
230	I	ILE230:A	-0.244	6	-0.427,-0.153	6,6	249/250	V,I,L
231	T	THR231:A	-0.845	8	-0.970,-0.764	8,8	249/250	A,I,F,M,G,T,V,C,N,S,L,Y
232	F	PHE232:A	0.444	4	0.084, 0.596	5,3	249/250	S,L,Y,E,K,V,C,H,T,I,F,M
233	D	ASP233:A	0.458	3	0.084, 0.596	5,3	247/250	C,Y,S,A,D,P,R,V,H,E,N,L,K,Q,I,M,G,T
234	S	SER234:A	0.895	2	0.393, 1.187	4,1	247/250	F,I,M,G,T,P,R,V,E,L,N,Q,K,A,D,C,Y,S
235	I	ILE235:A	1.069	1	0.596, 1.187	3,1	249/250	D,A,S,Y,T,G,M,I,F,K,Q,L,N,E,H,V,R,W,P
236	K	LYS236:A	1.127	1	0.596, 1.187	3,1	248/250	H,P,V,R,Q,K,E,L,N,M,I,T,G,C,S,A,D
237	R	ARG237:A	0.987	2	0.596, 1.187	3,1	249/250	H,R,V,W,P,K,Q,N,L,E,M,I,F,T,G,C,S,Y,A,D
238	E	GLU238:A	0.859	2	0.393, 1.187	4,1	245/250	I,A,G,D,T,V,P,H,S,L,N,E,Q,K
239	V	VAL239:A	0.665	3	0.227, 0.848	4,2	175/250	H,P,V,K,Q,Y,E,S,L,M,I,A,T,D,G
240	D	ASP240:A	0.360	4	0.084, 0.596	5,3	200/250	C,P,V,R,H,E,Y,L,S,K,F,A,D,G,T
241	E	GLU241:A	-0.071	5	-0.345, 0.084	6,5	220/250	V,K,Q,E,Y,S,N,M,I,T,D
242	E	GLU242:A	-0.108	5	-0.345, 0.084	6,5	244/250	A,G,D,T,V,P,H,L,N,S,E,Q,K
243	D	ASP243:A	0.072	5	-0.153, 0.227	6,4	247/250	T,D,G,A,Q,K,E,Y,N,L,S,H
244	A	ALA244:A	0.125	5	-0.153, 0.227	6,4	248/250	D,A,S,Y,C,G,T,I,F,M,N,L,E,K,Q,V,R,H
245	A	ALA245:A	-0.080	5	-0.345, 0.084	6,5	248/250	D,A,Y,S,T,M,I,Q,K,E,N,L,H,P,R,V
246	L	LEU246:A	0.146	5	-0.153, 0.227	6,4	248/250	H,V,R,C,K,Q,S,L,E,M,I,A,F,T,D
247	Y	TYR247:A	0.432	4	0.084, 0.596	5,3	248/250	T,M,I,F,Q,N,S,L,Y,H,C,W
248	R	ARG248:A	-0.016	5	-0.253, 0.084	6,5	249/250	H,R,V,C,K,Q,L,N,E,M,A,I,T,D
249	H	HIS249:A	0.269	4	-0.043, 0.393	5,4	248/250	H,P,V,R,Q,K,E,N,L,T,G,C,Y,S,A,D
250	L	LEU250:A	-0.573	7	-0.764,-0.427	8,6	249/250	L,W,V,G,T,F,I,A,M
251	G	GLY251:A	-0.263	6	-0.505,-0.153	7,6	249/250	T,D,G,M,A,I,E,L,C,V
252	T	THR252:A	-0.282	6	-0.505,-0.153	7,6	249/250	C,S,A,D,P,R,V,H,E,L,K,Q,F,I,M,G,T
253	L	LEU253:A	-0.409	6	-0.576,-0.345	7,6	249/250	I,R,V,P,M,L,S
254	L	LEU254:A	-0.260	6	-0.505,-0.153	7,6	249/250	M,C,V,I,T,S,L
255	R	ARG255:A	-0.570	7	-0.764,-0.505	8,7	249/250	T,G,A,K,Y,S,N,H,W,C,R
256	H	HIS256:A	-0.082	5	-0.345, 0.084	6,5	249/250	M,A,T,D,H,R,V,Q,K,Y,E,N,S,L
257	C	CYS257:A	-0.200	6	-0.427,-0.043	6,5	248/250	C,V,Y,N,L,S,F,I,M
258	V	VAL258:A	-0.806	8	-0.970,-0.706	8,7	248/250	F,C,I,V,M,D,L
259	M	MET259:A	-0.038	5	-0.253, 0.084	6,5	248/250	R,V,Y,L,S,M,F,I,T,G
260	L	LEU260:A	0.857	2	0.393, 1.187	4,1	248/250	A,I,F,T,R,V,C,H,N,S,L,Y,K
261	A	ALA261:A	1.996	1	1.187, 1.712	1,1	241/250	D,A,S,C,G,T,F,M,N,L,E,K,Q,V,R,P,H
262	A	ALA262:A	0.073	5	-0.153, 0.227	6,4	242/250	V,C,K,L,N,S,Y,E,I,A,T,G,D
263	A	ALA263:A	0.866	2	0.393, 1.187	4,1	240/250	N,L,S,E,Q,K,V,P,W,H,D,T,A,F,M
264	G	GLY264:A	0.091	5	-0.153, 0.227	6,4	234/250	C,S,A,D,P,R,V,K,Q,E,L,N,M,I,T,G
265	D	ASP265:A	0.670	3	0.227, 0.848	4,2	245/250	P,V,Q,K,E,S,L,N,M,I,A,D
266	R	ARG266:A	0.105	5	-0.153, 0.227	6,4	244/250	P,R,V,H,E,N,L,Q,K,F,I,M,G,T,S,A,D

267	T	THR267:A	-0.131	5	-0.345,-0.043	6,5	244/250	S,C,D,A,E,L,N,Q,K,V,R,H,G,T,I,M
268	E	GLU268:A	0.071	5	-0.153, 0.227	6,4	248/250	S,Y,C,D,A,N,L,E,Q,K,R,V,W,P,H,I,M
269	E	GLU269:A	-0.335	6	-0.505,-0.253	7,6	248/250	E,S,N,L,Q,A,D,G,T
270	L	LEU270:A	-0.344	6	-0.576,-0.253	7,6	248/250	T,F,I,A,M,L,S,V
271	H	HIS271:A	0.002	5	-0.253, 0.084	6,5	249/250	L,N,E,Y,Q,K,R,V,W,C,H,D,T,I,M
272	G	GLY272:A	-0.581	7	-0.764,-0.505	8,7	249/250	R,C,S,N,K,A,M,G,T
273	H	HIS273:A	-0.827	8	-0.970,-0.764	8,8	250/250	H,N,S,E,Y,Q,F,M,D
274	A	ALA274:A	-0.334	6	-0.505,-0.253	7,6	250/250	T,S,I,V,A,C
275	V	VAL275:A	-0.472	7	-0.643,-0.345	7,6	249/250	T,L,G,M,V,A,I
276	N	ASN276:A	-1.229	9	-1.286,-1.216	9,9	249/250	H,S,N,D
277	L	LEU277:A	-0.904	8	-1.059,-0.820	9,8	247/250	M,A,V,I,F,T,L
278	L	LEU278:A	-1.144	9	-1.252,-1.101	9,9	249/250	I,F,M,L
279	G	GLY279:A	-0.850	8	-0.970,-0.764	8,8	249/250	G,T,I,M,N,S,Y,V
280	N	ASN280:A	-0.932	8	-1.059,-0.872	9,8	249/250	A,T,V,C,P,S,N,L,Y
281	L	LEU281:A	-0.477	7	-0.643,-0.345	7,6	249/250	L,C,A,V,I,M
282	P	PRO282:A	-1.022	8	-1.141,-0.970	9,8	249/250	I,P,H,L,N,S,K
283	V	VAL283:A	0.541	3	0.227, 0.848	4,2	249/250	S,Y,D,A,K,N,L,E,H,R,V,P,T,G,I,F
284	K	LYS284:A	0.436	4	0.084, 0.596	5,3	249/250	T,G,M,F,K,Q,N,L,E,H,R,V,P,D,A,S,Y,C
285	C	CYS285:A	-0.229	6	-0.505,-0.043	7,5	249/250	G,T,F,A,M,Y,N,S,Q,C,H
286	L	LEU286:A	-0.603	7	-0.820,-0.505	8,7	249/250	L,Y,Q,V,P,H,F,M
287	D	ASP287:A	-0.262	6	-0.505,-0.153	7,6	249/250	Q,K,E,N,S,H,P,V,R,T,D,G,M,A
288	V	VAL288:A	-0.458	7	-0.643,-0.345	7,6	249/250	N,L,S,Y,E,K,Q,V,P,G,T,I,A,F,M
289	L	LEU289:A	-0.908	8	-1.059,-0.820	9,8	249/250	L,D,K,I,V,M
290	L	LEU290:A	-0.092	5	-0.345, 0.084	6,5	249/250	V,H,Y,L,F,A,I,M,T
291	T	THR291:A	0.888	2	0.393, 1.187	4,1	248/250	C,S,A,D,H,P,W,V,R,Q,E,N,L,X,M,F,I,T
292	L	LEU292:A	0.407	4	0.084, 0.596	5,3	246/250	K,Q,S,L,E,V,P,T,G,D,M,I,A
293	E	GLU293:A	1.398	1	0.848, 1.712	2,1	247/250	T,G,M,I,F,Q,K,N,L,E,H,R,V,P,W,D,A,S,Y,C
294	P	PRO294:A	1.012	2	0.596, 1.187	3,1	248/250	T,G,F,I,K,Q,E,N,L,H,P,V,D,A,Y,S,C
295	H	HIS295:A	1.666	1	1.187, 1.712	1,1	248/250	G,T,F,I,E,N,L,K,Q,P,R,V,H,D,A,Y,S
296	E	GLU296:A	1.340	1	0.848, 1.712	2,1	177/250	A,T,G,H,R,V,P,Q,K,S,L,N,E
297	G	GLY297:A	0.320	4	-0.043, 0.596	5,3	175/250	H,P,E,L,N,S,M,A,D,G
298	S	SER298:A	0.421	4	0.084, 0.596	5,3	245/250	H,C,R,V,K,Q,E,L,S,N,A,I,T,D,G
299	L	LEU299:A	2.332	1	1.187, 3.096	1,1	245/250	T,G,M,I,F,Q,K,L,N,E,H,R,V,P,D,A,S,Y,C
300	E	GLU300:A	0.542	3	0.227, 0.848	4,2	248/250	S,C,D,A,Q,K,L,N,E,H,R,V,P,W,T,G,M,I,F
301	F	PHE301:A	0.786	2	0.393, 0.848	4,2	250/250	M,F,A,I,T,H,W,C,R,V,K,Y,L,S,N
302	L	LEU302:A	0.234	4	-0.043, 0.393	5,4	249/250	M,F,D,G,H,P,R,K,Q,E,N,L,S
303	G	GLY303:A	-0.070	5	-0.345, 0.084	6,5	250/250	Q,K,E,N,S,H,P,W,C,T,D,G,A
304	V	VAL304:A	0.422	4	0.084, 0.596	5,3	250/250	T,F,I,M,E,L,N,K,Q,W,P,V,R,H,D,A,Y,C
305	N	ASN305:A	-0.903	8	-1.016,-0.820	8,8	250/250	D,S,G,N,K,V,R
306	M	MET306:A	-0.804	8	-0.922,-0.706	8,7	250/250	T,L,M,C,F,I,V

307	D	ASP307:A	0.087	5	-0.153, 0.227	6,4	250/250
Y,E,N,S,Q,K,R,H,D,G,T,F,I,A,M							
308	V	VAL308:A	-0.589	7	-0.764,-0.505	8,7	250/250
C,P,V,E,S,L,I,A,T,G							
309	I	ILE309:A	-0.299	6	-0.505,-0.153	7,6	250/250
L,M,V,I,F,P							
310	R	ARG310:A	1.696	1	1.187, 1.712	1,1	250/250
H,V,R,Q,K,E,L,N,M,F,I,T,G,C,Y,S,A,D							
311	V	VAL311:A	0.785	2	0.393, 0.848	4,2	250/250
Q,K,S,L,N,E,V,C,T,M,I,A							
312	L	LEU312:A	-0.658	7	-0.820,-0.576	8,7	250/250
L,T,V,I,F,M							
313	L	LEU313:A	-0.467	7	-0.643,-0.345	7,6	250/250
M,I,V,C,F,T,L							
314	S	SER314:A	1.823	1	1.187, 1.712	1,1	250/250
A,D,Y,S,M,I,T,G,H,R,V,Q,K,E,L,N							
315	F	PHE315:A	-0.497	7	-0.706,-0.345	7,6	250/250
Q,Y,L,H,F,V,I							
316	M	MET316:A	-1.023	8	-1.141,-0.970	9,8	249/250
L,T,I,V,F,M							
317	E	GLU317:A	0.001	5	-0.253, 0.084	6,5	249/250
S,L,N,E,Y,Q,K,R,H,G,D,T,I							
318	K	LYS318:A	1.197	1	0.596, 1.187	3,1	249/250
K,Q,E,Y,N,S,L,H,R,V,T,D,G,I,A							
319	R	ARG319:A	-0.438	6	-0.643,-0.345	7,6	249/250
M,I,A,T,G,H,R,V,K,Q,N,Y							
320	L	LEU320:A	-0.907	8	-1.059,-0.820	9,8	249/250
M,I,V,R,C,F,L							
321	H	HIS321:A	0.273	4	-0.043, 0.393	5,4	249/250
T,G,D,M,A,Q,K,S,L,N,E,H,R,C							
322	Q	GLN322:A	0.486	3	0.084, 0.596	5,3	249/250
M,I,T,H,R,V,P,Q,K,N,L,E,A,D,C,S,Y							
323	T	THR323:A	0.481	3	0.084, 0.596	5,3	244/250
I,A,M,G,T,R,V,P,H,N,S,E,Q,K							
324	H	HIS324:A	0.670	3	0.227, 0.848	4,2	245/250
C,Y,S,A,D,P,V,R,H,E,N,L,K,Q,F,I,M,G,T							
325	R	ARG325:A	0.127	5	-0.153, 0.227	6,4	249/250
N,S,Q,K,P,C,V,R,H,D,G,T,I,A,M							
326	L	LEU326:A	0.116	5	-0.153, 0.227	6,4	249/250
Q,K,E,N,L,H,P,R,V,T,M,F,I,Y,S,D							
327	K	LYS327:A	0.300	4	-0.043, 0.393	5,4	249/250
S,Y,D,A,Q,K,N,L,H,R,V,P,T,G,I,F							
328	E	GLU328:A	-0.842	8	-0.970,-0.764	8,8	249/250
A,G,D,P,Q,S,N,E							
329	S	SER329:A	0.777	2	0.393, 0.848	4,2	248/250
H,R,V,P,Q,K,L,N,E,M,I,F,T,G,C,S,Y,A,D							
330	V	VAL330:A	-0.419	6	-0.643,-0.253	7,6	248/250
S,L,M,F,A,V,I							
331	A	ALA331:A	-0.559	7	-0.706,-0.505	7,7	246/250
M,A,T,P,C,R,V,Q,E,N,L,S							
332	P	PRO332:A	-1.263	9	-1.352,-1.216	9,9	247/250
D,P,A							
333	V	VAL333:A	-0.613	7	-0.764,-0.505	8,7	249/250
F,I,A,T,W,C,V,E,S,L							
334	L	LEU334:A	-0.844	8	-0.970,-0.764	8,8	249/250
I,V,F,M,L,Q							
335	S	SER335:A	-0.500	7	-0.643,-0.427	7,6	249/250
Q,E,S,L,N,H,C,W,V,T,M,F,A,I							
336	V	VAL336:A	-0.557	7	-0.706,-0.427	7,6	249/250
H,C,V,S,L,M,F,A,I,T							
337	L	LEU337:A	-0.856	8	-1.016,-0.764	8,8	248/250
L,M,F,C,W,A							
338	T	THR338:A	-0.540	7	-0.706,-0.427	7,6	248/250
S,N,L,V,C,T,M,I,A,F							
339	E	GLU339:A	-0.470	7	-0.643,-0.345	7,6	248/250
A,G,T,C,R,V,H,E,N,S,K,Q							
340	C	CYS340:A	-0.119	5	-0.345, 0.084	6,5	248/250
T,G,M,F,A,I,L,S,C,V							
341	A	ALA341:A	-0.455	7	-0.643,-0.345	7,6	248/250
S,T,V,I,A,C							
342	R	ARG342:A	-0.675	7	-0.820,-0.576	8,7	248/250
I,A,T,H,R,C,K,Q,S,N,E							
343	M	MET343:A	0.612	3	0.227, 0.848	4,2	249/250
M,I,F,T,G,H,R,V,Q,K,N,L,A,C,S,Y							
344	H	HIS344:A	-0.624	7	-0.764,-0.505	8,7	248/250
D,G,I,E,N,S,Q,K,C,V,H							
345	R	ARG345:A	-0.805	8	-0.922,-0.706	8,7	249/250
K,D,S,G,P,A,R							
346	P	PRO346:A	0.657	3	0.227, 0.848	4,2	249/250
L,N,E,Q,K,V,P,G,T,I,F,M,S,Y,C,D,A							

347	A	ALA347:A	-0.483	7	-0.643,-0.427	7,6	249/250	T,I,A,F,M,L,N,Y,Q,V,P,H
348	R	ARG348:A	-1.266	9	-1.320,-1.252	9,9	248/250	R,C,P,F
349	K	LYS349:A	-0.562	7	-0.706,-0.427	7,6	248/250	M,H,R,P,C,K,L,E
350	F	PHE350:A	-0.409	6	-0.643,-0.253	7,6	248/250	H,A,W,F,G,Y,E
351	L	LEU351:A	-0.391	6	-0.576,-0.253	7,6	248/250	L,Q,C,P,V,T,F,I
352	K	LYS352:A	-0.930	8	-1.059,-0.872	9,8	248/250	R,N,S,G,K
353	A	ALA353:A	0.545	3	0.227, 0.596	4,3	248/250	N,L,E,Q,K,V,R,W,P,H,T,I,F,M,S,Y,D,A
354	Q	GLN354:A	0.059	5	-0.153, 0.227	6,4	246/250	N,E,Y,K,Q,V,R,H,I,A,M
355	V	VAL355:A	-0.759	8	-0.922,-0.706	8,7	245/250	L,R,I,V
356	L	LEU356:A	-1.295	9	-1.352,-1.252	9,9	245/250	M,L
357	P	PRO357:A	-1.227	9	-1.320,-1.179	9,9	247/250	T,G,P,A
358	P	PRO358:A	-0.954	8	-1.101,-0.872	9,8	247/250	K,T,Y,L,N,H,P
359	L	LEU359:A	-1.213	9	-1.320,-1.179	9,9	247/250	G,L,M,R
360	R	ARG360:A	-0.256	6	-0.505,-0.153	7,6	247/250	G,T,A,M,S,Q,K,R,V
361	D	ASP361:A	-0.946	8	-1.059,-0.872	9,8	247/250	A,Q,D,E,G,N
362	V	VAL362:A	-1.103	9	-1.179,-1.059	9,9	248/250	S,L,M,I,A,V,F
363	R	ARG363:A	0.300	4	-0.043, 0.393	5,4	248/250	A,D,Y,S,F,I,M,G,T,W,R,H,E,L,K,Q
364	T	THR364:A	0.632	3	0.227, 0.848	4,2	248/250	M,I,T,D,H,C,V,R,K,Q,E,L,S,N
365	R	ARG365:A	-1.014	8	-1.141,-0.970	9,8	248/250	K,E,L,P,R
366	P	PRO366:A	-1.301	9	-1.368,-1.286	9,9	248/250	A,P
367	E	GLU367:A	-1.230	9	-1.320,-1.179	9,9	246/250	W,Q,D,E,G
368	V	VAL368:A	-0.312	6	-0.505,-0.153	7,6	248/250	E,L,N,Q,K,V,H,D,G,T,I
369	G	GLY369:A	-0.990	8	-1.141,-0.922	9,8	248/250	D,E,L,G,T
370	E	GLU370:A	0.645	3	0.227, 0.848	4,2	249/250	H,V,P,K,L,S,N,E,M,A,T,G,D
371	L	LEU371:A	-0.421	6	-0.576,-0.345	7,6	249/250	G,T,A,M,E,L,S,K,Q,H
372	L	LEU372:A	-0.177	6	-0.427,-0.043	6,5	249/250	T,I,F,M,L,S,R,V
373	R	ARG373:A	-1.192	9	-1.286,-1.141	9,9	249/250	R,K,Y,S
374	N	ASN374:A	-1.015	8	-1.101,-0.970	9,8	249/250	F,A,V,G,N,S
375	K	LYS375:A	-0.591	7	-0.764,-0.505	8,7	249/250	H,R,P,K,Q,L,S,N,Y,M,I
376	L	LEU376:A	-0.975	8	-1.101,-0.922	9,8	249/250	L,C,F,V,I,M
377	V	VAL377:A	-1.050	9	-1.141,-0.970	9,8	249/250	S,V,A,I,P,C
378	R	ARG378:A	-0.913	8	-1.016,-0.820	8,8	249/250	C,R,V,K,Q,S,G
379	L	LEU379:A	-0.823	8	-0.970,-0.706	8,7	249/250	I,F,M,T,R,V,C,H,L,Y
380	M	MET380:A	-1.042	9	-1.141,-0.970	9,8	249/250	M,I,F,Q,N,L,R,C
381	T	THR381:A	-1.222	9	-1.286,-1.179	9,9	249/250	G,T,A,F,M,H
382	H	HIS382:A	-0.740	7	-0.872,-0.643	8,7	248/250	T,A,S,N,L,Y,C,H
383	L	LEU383:A	-0.532	7	-0.706,-0.427	7,6	246/250	T,M,A,I,K,N,L,S,P,C,V
384	D	ASP384:A	-0.240	6	-0.427,-0.153	6,6	247/250	G,D,M,I,A,F,Q,N,S,L,E,H,V,C
385	T	THR385:A	-0.674	7	-0.820,-0.576	8,7	246/250	D,G,T,F,I,A,M,S,L,N,V
386	D	ASP386:A	-0.294	6	-0.505,-0.153	7,6	249/250	C,R,V,H,E,N,S,L,Q,A,I,M,D,G,T

387	V	VAL387:A	-0.635	7	-0.764,-0.576	8,7	249/250	A,I,V,L,T
388	K	LYS388:A	-0.906	8	-1.016,-0.820	8,8	249/250	A,T,R,V,H,N,S,Y,E,K
389	R	ARG389:A	-0.337	6	-0.505,-0.253	7,6	249/250	A,F,M,G,D,T,R,C,H,S,L,N,E,Y,Q
390	V	VAL390:A	-0.326	6	-0.505,-0.153	7,6	249/250	G,T,A,I,M,S,N,L,W,P,C,V
391	A	ALA391:A	-0.723	7	-0.872,-0.643	8,7	249/250	I,V,A,S,T
392	A	ALA392:A	-1.153	9	-1.252,-1.101	9,9	249/250	A,T,D,G,C,R,E,Y,S
393	E	GLU393:A	-0.749	8	-0.922,-0.643	8,7	249/250	S,L,E,Q,R,H,D,T
394	F	PHE394:A	-0.598	7	-0.764,-0.505	8,7	247/250	L,E,V,F
395	L	LEU395:A	-1.030	8	-1.141,-0.970	9,8	249/250	L,N,I,C
396	F	PHE396:A	-1.054	9	-1.179,-0.970	9,8	249/250	Y,L,S,C,F
397	V	VAL397:A	-0.821	8	-0.970,-0.764	8,8	249/250	A,V,I,T,S,L
398	L	LEU398:A	-1.103	9	-1.216,-1.059	9,9	249/250	L,S,F,C,V,M
399	C	CYS399:A	-1.280	9	-1.352,-1.252	9,9	249/250	P,C
400	S	SER400:A	-1.143	9	-1.252,-1.101	9,9	248/250	K,S,N,R,I,A
401	E	GLU401:A	-1.005	8	-1.141,-0.922	9,8	249/250	K,Q,S,E,W,C,T,G,A,F
402	S	SER402:A	-0.718	7	-0.872,-0.643	8,7	249/250	K,N,S,P,R,T,D,A
403	V	VAL403:A	-1.151	9	-1.252,-1.101	9,9	249/250	Q,T,S,A,V,P
404	P	PRO404:A	0.229	4	-0.043, 0.393	5,4	249/250	A,M,G,D,T,R,P,N,S,L,K,Q
405	R	ARG405:A	-1.149	9	-1.252,-1.101	9,9	249/250	R,H,N,S,T,K
406	F	PHE406:A	-1.021	8	-1.141,-0.970	9,8	249/250	L,Y,M,A,F
407	I	ILE407:A	-0.904	8	-1.016,-0.820	8,8	249/250	L,I,V
408	K	LYS408:A	-1.337	9	-1.368,-1.320	9,9	249/250	K
409	Y	TYR409:A	-1.126	9	-1.252,-1.059	9,9	248/250	C,H,Y
410	T	THR410:A	-1.290	9	-1.352,-1.252	9,9	248/250	F,S,T
411	G	GLY411:A	-1.328	9	-1.368,-1.320	9,9	249/250	G
412	Y	TYR412:A	-1.035	8	-1.141,-0.970	9,8	249/250	Y,F
413	G	GLY413:A	-1.294	9	-1.352,-1.252	9,9	249/250	S,G
414	N	ASN414:A	-1.331	9	-1.368,-1.320	9,9	249/250	G,N
415	A	ALA415:A	-1.195	9	-1.286,-1.141	9,9	249/250	C,F,A,M,S,G
416	A	ALA416:A	-1.308	9	-1.352,-1.286	9,9	249/250	K,V,A
417	G	GLY417:A	-1.328	9	-1.368,-1.320	9,9	249/250	G
418	L	LEU418:A	-0.965	8	-1.101,-0.872	9,8	249/250	I,F,M,L,N,Q,W,H
419	L	LEU419:A	-1.026	8	-1.141,-0.970	9,8	249/250	M,F,L,S
420	A	ALA420:A	-1.137	9	-1.216,-1.101	9,9	249/250	S,C,A,V,R,M
421	A	ALA421:A	-0.466	7	-0.643,-0.345	7,6	249/250	H,R,Q,K,N,S,E,A,T,D
422	R	ARG422:A	-0.933	8	-1.059,-0.872	9,8	249/250	R,H,L,S,N,K,F,A
423	G	GLY423:A	-1.213	9	-1.320,-1.179	9,9	249/250	K,G,D,V
424	L	LEU424:A	-0.993	8	-1.101,-0.922	9,8	249/250	M,V,A,I,W,L
425	M	MET425:A	-0.738	7	-0.872,-0.643	8,7	249/250	V,M,L
426	A	ALA426:A	-0.031	5	-0.253, 0.084	6,5	249/250	T,G,D,M,A,F,Q,K,S,L,N,H,R,C

427	G	GLY427:A	-0.689	7	-0.872,-0.576	8,7	248/250	K,Q,L,S,C,P,R,T,G
428	G	GLY428:A	-0.012	5	-0.253, 0.084	6,5	245/250	E,Y,N,S,Q,K,P,V,R,H,D,G,T,A,I
429	R	ARG429:A	0.381	4	0.084, 0.596	5,3	244/250	K,Q,N,S,H,R,P,C,W,T,G,D,M,A,F
430	P	PRO430:A	0.769	2	0.393, 1.187	4,1	179/250	E,S,N,P,C,D,G,T,A,M
431	E	GLU431:A	1.178	1	0.596, 1.187	3,1	248/250	Q,K,E,S,N,H,P,C,R,V,T,D,G,A
432	G	GLY432:A	0.738	3	0.393, 0.848	4,2	248/250	R,V,P,C,H,N,L,S,E,Q,A,G,D,T
433	Q	GLN433:A	1.243	1	0.848, 1.712	2,1	247/250	T,G,F,I,K,Q,E,N,L,H,P,W,V,R,D,A,S,C
434	Y	TYR434:A	-1.074	9	-1.179,-1.016	9,8	246/250	Y,D,N,K,F,H,X
435	S	SER435:A	-1.197	9	-1.286,-1.179	9,9	249/250	E,D,S,T,F
436	E	GLU436:A	-0.286	6	-0.505,-0.153	7,6	249/250	P,C,V,E,N,S,A,D,G
437	D	ASP437:A	-1.122	9	-1.216,-1.059	9,9	249/250	E,D,G,S
438	E	GLU438:A	-0.697	7	-0.872,-0.576	8,7	246/250	D,E,S,G,T
439	D	ASP439:A	-0.858	8	-0.970,-0.764	8,8	249/250	N,G,E,D,F
440	T	THR440:A	-1.243	9	-1.320,-1.216	9,9	249/250	R,T,S
441	D	ASP441:A	-1.000	8	-1.101,-0.922	9,8	249/250	V,E,D,G,S
442	T	THR442:A	-1.312	9	-1.352,-1.286	9,9	249/250	R,T,S
443	D	ASP443:A	-1.014	8	-1.141,-0.970	9,8	249/250	D,E,P
444	E	GLU444:A	-1.202	9	-1.286,-1.179	9,9	249/250	T,E,D,G,M,I
445	Y	TYR445:A	-1.288	9	-1.352,-1.252	9,9	249/250	Y,T
446	K	LYS446:A	0.347	4	0.084, 0.596	5,3	249/250	A,I,T,R,V,H,L,N,S,E,K,Q
447	E	GLU447:A	-0.020	5	-0.253, 0.084	6,5	249/250	T,D,M,A,I,K,Q,N,L,S,E,R,V
448	A	ALA448:A	-0.168	6	-0.427,-0.043	6,5	249/250	Y,N,L,Q,V,H,G,F,A,I,M
449	K	LYS449:A	-0.490	7	-0.643,-0.345	7,6	249/250	R,A,F,Q,K,S,E
450	A	-	-0.088	5	-0.345, 0.084	6,5	248/250	T,G,D,A,Q,S,N,E,H,R,P
451	S	SER450:A	0.640	3	0.227, 0.848	4,2	245/250	D,G,M,F,A,K,Q,E,L,S,N,H,C,R
452	I	ILE451:A	-1.050	9	-1.141,-1.016	9,8	245/250	L,Y,I,V,F
453	N	ASN452:A	-1.163	9	-1.252,-1.141	9,9	245/250	D,E,N,V,H
454	P	-	-1.047	9	-1.179,-0.970	9,8	245/250	S,L,V,I,P
455	V	-	-0.920	8	-1.016,-0.872	8,8	244/250	T,E,M,I,V,P
456	T	-	-0.674	7	-0.820,-0.576	8,7	245/250	V,I,S,L,Y,E,T
457	G	-	-1.293	9	-1.352,-1.252	9,9	244/250	P,G
458	R	-	-0.741	7	-0.872,-0.643	8,7	244/250	H,W,C,R,V,Q,S,L,A
459	V	-	-0.284	6	-0.505,-0.153	7,6	244/250	Y,L,C,W,R,V,T,F,I,M
460	E	-	-0.704	7	-0.872,-0.643	8,7	244/250	F,I,M,D,T,P,R,V,E,Q,K
461	E	-	0.080	5	-0.153, 0.227	6,4	242/250	V,P,H,L,S,E,Q,K,A,M,G,D,T
462	K	-	0.174	4	-0.153, 0.393	6,4	242/250	S,N,Y,E,K,Q,V,P,H,D,A,X
463	P	-	-0.154	6	-0.345,-0.043	6,5	243/250	T,G,M,I,A,K,Q,E,S,L,H,P,R
464	P	-	-0.173	6	-0.427,-0.043	6,5	243/250	S,N,L,K,R,V,P,C,H,D,T,I,A,M
465	N	-	-0.708	7	-0.872,-0.643	8,7	243/250	N,S,G,D,Q
466	P	-	-0.969	8	-1.101,-0.872	9,8	243/250	S,L,P,C,V,A,I

467	M	-	-0.295	6	-0.505,-0.153	7,6	243/250	L,T,W,F,I,V,M
468	E	-	-0.569	7	-0.764,-0.505	8,7	242/250	R,C,E,Q,K,A,X,D
469	G	-	-0.420	6	-0.643,-0.253	7,6	242/250	D,G,Q,E,N,S,H,C
470	M	-	-1.195	9	-1.286,-1.141	9,9	242/250	L,Q,I,M
471	T	-	-0.860	8	-0.970,-0.820	8,8	240/250	T,E,S
472	E	-	-0.563	7	-0.706,-0.427	7,6	240/250	V,D,E,Q
473	E	-	-1.205	9	-1.286,-1.179	9,9	241/250	D,E,Q,T
474	Q	-	-1.170	9	-1.252,-1.141	9,9	241/250	R,E,Q,K
475	K	-	-1.226	9	-1.320,-1.179	9,9	241/250	M,Q,K
476	E	-	-1.288	9	-1.352,-1.252	9,9	241/250	E,Q
477	H	-	0.274	4	-0.043, 0.393	5,4	241/250	D,F,I,A,Q,K,E,Y,L,H,C,R,V
478	E	-	-1.048	9	-1.141,-0.970	9,8	242/250	L,E,H,V,P
479	A	-	-1.243	9	-1.320,-1.216	9,9	244/250	A,V,F,S
480	M	-	-0.278	6	-0.505,-0.153	7,6	244/250	C,V,R,H,E,L,N,Q,K,I,M
481	K	-	-0.292	6	-0.505,-0.153	7,6	244/250	Q,K,N,S,E,H,R,G,D
482	L	-	-1.295	9	-1.352,-1.286	9,9	244/250	L,P
483	V	-	-0.575	7	-0.706,-0.505	7,7	244/250	S,L,V,G,T,A,I,M
484	N	-	-0.189	6	-0.427,-0.043	6,5	244/250	T,D,G,M,A,Q,K,E,L,N,S,H,C,R,V
485	M	-	-0.424	6	-0.643,-0.345	7,6	244/250	T,G,D,M,A,F,K,Q,L,S,E,R,V
486	F	-	-0.377	6	-0.576,-0.253	7,6	244/250	M,C,F,V,I,S,L
487	D	-	-0.703	7	-0.872,-0.643	8,7	244/250	N,S,E,H,V,P,T,G,D,A
488	K	-	-0.175	6	-0.427,-0.043	6,5	243/250	K,Q,E,S,N,H,R,T,M,F
489	L	-	-1.033	8	-1.141,-0.970	9,8	243/250	L,T,A,V,P,M
490	S	-	-0.612	7	-0.764,-0.505	8,7	244/250	H,V,R,C,Q,N,S,L,Y,M,I,A,T,D
491	R	-	-0.632	7	-0.764,-0.576	8,7	241/250	C,R,D,S,T,K,Q
492	H	-	1.376	1	0.848, 1.712	2,1	237/250	H,R,K,Q,Y,E,L,S,N,M,A,I,T,D,G
493	R	-	-0.031	5	-0.253, 0.084	6,5	234/250	G,D,X,S,N,E,Q,K,R,V,H
494	V	-	0.101	5	-0.153, 0.227	6,4	230/250	R,V,S,L,Q,F,I,M,T
495	I	-	-0.850	8	-0.970,-0.764	8,8	229/250	M,F,I,V,T,N,L
496	Q	-	-0.751	8	-0.872,-0.643	8,7	231/250	N,E,Q,K,R,H,T,M
497	P	-	-1.192	9	-1.286,-1.141	9,9	231/250	P,A,T
498	M	-	-0.605	7	-0.764,-0.505	8,7	231/250	M,I,A,T,G,C,V,L,S
499	G	-	-0.072	5	-0.345, 0.084	6,5	230/250	G,T,A,M,N,L,S,E,K,Q,V,R,C,H
500	M	-	-0.675	7	-0.820,-0.576	8,7	230/250	M,F,I,V,L
501	S	-	-0.444	6	-0.643,-0.345	7,6	229/250	K,L,S,N,R,V,C,T,G,D,M
502	P	-	0.927	2	0.393, 1.187	4,1	227/250	D,G,T,A,X,M,E,L,S,K,P,C,R,V
503	R	-	-0.806	8	-0.922,-0.706	8,7	228/250	D,G,E,S,N,Q,C,R
504	G	-	-1.325	9	-1.368,-1.320	9,9	228/250	G
505	Q	-	0.013	5	-0.253, 0.084	6,5	226/250	G,T,A,S,L,N,Q,K,R,H
506	L	-	-0.845	8	-1.016,-0.764	8,8	215/250	M,I,V,F,P,L

507	T	-	-0.096	5	-0.345, 0.084	6,5	210/250	H,V,R,K,Q,S,Y,E,A,I,T,G
508	S	-	-0.308	6	-0.505,-0.153	7,6	205/250	T,A,E,S,K,Q,P,V
509	L	-	-0.416	6	-0.643,-0.253	7,6	202/250	L,F,A,I,V,M
510	Q	-	0.368	4	0.084, 0.596	5,3	186/250	A,D,G,T,C,W,R,H,Y,E,S,L,N,K,Q
511	D	-	-0.108	5	-0.345, 0.084	6,5	176/250	L,N,S,E,K,Q,R,V,P,H,G,D,T,A
512	A	-	0.166	4	-0.153, 0.393	6,4	167/250	T,D,G,A,I,K,Q,E,L,N,H,P,V,R
513	M	-	1.060	1	0.596, 1.187	3,1	156/250	G,D,A,I,M,L,N,E,Q,K,V,R
514	C	-	1.058	1	0.596, 1.187	3,1	154/250	G,D,A,I,F,N,S,L,E,Q,V,R,C,P,H
515	E	-	1.604	1	0.848, 1.712	2,1	149/250	R,C,H,L,S,N,E,Q,K,A,F,M,G,D,T
516	T	-	1.124	1	0.596, 1.187	3,1	147/250	Y,L,S,Q,K,C,P,R,V,H,T,I,A,M
517	M	-	1.194	1	0.596, 1.712	3,1	144/250	M,I,A,T,H,P,V,R,K,Q,E,N,S,L
518	E	-	1.682	1	0.848, 1.712	2,1	140/250	S,N,L,E,Q,R,V,P,G,D,T,A,I
519	G	-	3.027	1	1.712, 3.096	1,1	132/250	M,I,T,G,H,P,R,K,Q,E,L,N,A,D,C,S
520	Q	-	1.386	1	0.848, 1.712	2,1	133/250	A,G,D,T,R,C,P,H,S,L,N,E,Q,K
521	L	-	2.901	1	1.712, 3.096	1,1	128/250	D,A,S,C,T,G,M,I,Q,K,L,N,E,V,R,P
522	S	-	0.513	3	0.084, 0.848	5,2	126/250	T,D,G,H,P,C,R,Q,K,E,N,S,L
523	S	-	-0.128	5	-0.427, 0.084	6,5	122/250	A,I,F,D,T,R,P,N,S,L,E,Q
524	D	-	0.220	4	-0.153, 0.393	6,4	116/250	D,T,E,S,N,L,Q,P,H
525	P	-	-0.066	5	-0.427, 0.084	6,5	96/250	P,H,S,N,L,E,A,D,T
526	D	-	0.248	4	-0.153, 0.596	6,3	92/250	S,N,E,Q,K,R,P,D,A
527	S	-	-0.287	6	-0.643,-0.043	7,5	68/250	E,D,S,L,R
528	D	-	-0.305	6	-0.643,-0.043	7,5	54/250	A,P,L,S,D,E
529	P	-	0.325	4	-0.253, 0.596	6,3	48/250	T,S,D,E,A,P
530	D	-	-0.981	8	-1.216,-0.872	9,8	44/250	D,S

*Below the confidence cut-off - The calculations for this site were performed on less than 6 non-gaped homologue sequences, or the confidence interval for the estimated score is equal to- or larger than- 4 color grades.
