# Supplementary material for: Structural underpinnings of Ric8A function as a G-protein α-subunit chaperone and guanine-nucleotide exchange factor
Source: Nat Commun. 2019 Jul 12;10:3084. doi: 10.1038/s41467-019-11088-x (PMC6625990; doi:10.1038/s41467-019-11088-x)
Supplement: Supplementary file 5 — Supplementary Data 2 [file 41467_2019_11088_MOESM5_ESM.pdf]

Supplementary Data 2. Intramolecular DSS-crosslinked peptides of apo Ric8A1-492

m/z	z	ppm	Crosslinked Peptide	RT	#	Score	Score Diff	Expect	MS-Tag Score	Exp	Rank	Low Score	XLink AA
801.9627	4	-0.19	FIK(+DSS)YTGYGNAAGLLAAR<br>FLK(+DSS)AQVLPPLRFIK(+DSS)YTGYGNAAGLLAAR<br>FLK(+DSS)AQVLPPLR	42.28442.284	1	56.5	20.1	1.5e-8	36.4<br>26.8	9.3e-4<br>0.18	1<br>7	20.1	408<br>352
792.1819	4	0.083	FIK(+DSS)YTGYGNAAGLLAAR<br>EAK(+DSS)ASINPVTGRFIK(+DSS)YTGYGNAAGLLAAR<br>EAK(+DSS)ASINPVTGR	36.38136.381	1	52.0	19.8	1.7e-7	32.2<br>20.3	0.0052<br>2.6	1<br>3	19.8	408<br>449
873.0071	4	0.97	K(Xlink:DSS1)FLK(+DSS)AQVLPPLR<br>FIK(+DSS)YTGYGNAAGLLAARK(Xlink:DSS1)FLK(+DSS)AQVLPPLR<br>FIK(+DSS)YTGYGNAAGLLAAR	45.24945.249	1	51.2	21.6	2.8e-7	27.4<br>25.5	0.091<br>0.25	5<br>15	23.8	352<br>408
820.1336	3	0.23	FIK(+DSS)YTGYGNAAGLLAAR<br>K(+DSS)FLKFIK(+DSS)YTGYGNAAGLLAAR<br>K(+DSS)FLK	36.34236.342	1	48.0	10.0	8.4e-7	38.0<br>14.2	2.7e-4<br>266	1<br>81	14.1	408<br>349
666.1409	4	-1.2	FLK(+DSS)AQVLPPLR<br>EAK(+DSS)ASINPVTGRFLK(+DSS)AQVLPPLR<br>EAK(+DSS)ASINPVTGR	34.92034.920	1	55.0	20.1	1.1e-6	32.5<br>28.6	0.032<br>0.19	7<br>16	24.6	352<br>449
801.9617	4	-1.4	FLK(+DSS)AQVLPPLR<br>FIK(+DSS)YTGYGNAAGLLAARFLK(+DSS)AQVLPPLR<br>FIK(+DSS)YTGYGNAAGLLAAR	42.85342.853	1	43.2	15.4	1.4e-6	26.3<br>21.2	0.030<br>0.60	5<br>17	16.9	352<br>408
630.3728	3	-0.13	VLLSFMEK(+DSS)R<br>NK(+DSS)LVRVLLSFMEK(+DSS)R<br>NK(+DSS)LVR	33.77533.775	1	57.4	9.4	6.6e-5	48.0<br>19.4	0.0017<br>34	1<br>7	16.9	318<br>375
512.8249	4	1.7	FLK(+DSS)AQVLPPLR<br>NK(+DSS)LVRFLK(+DSS)AQVLPPLR<br>NK(+DSS)LVR	32.80332.803	1	50.4	6.2	1.1e-4	44.2<br>17.6	0.0013<br>46	1<br>20	12.5	352<br>375
477.0297	4	-1.1	VLLSFM(Oxidation)EK(+DSS)R<br>NK(+DSS)LVRVLLSFM(Oxidation)EK(+DSS)R<br>NK(+DSS)LVR	29.32729.327	1	43.5	6.1	1.7e-4	37.4<br>6.3	0.0019<br>402	1<br>76	6.1	318<br>375
630.3730	3	0.18	VLLSFMEK(+DSS)R<br>NK(+DSS)LVRVLLSFMEK(+DSS)R<br>NK(+DSS)LVR	33.44433.444	1	42.5	9.5	3.3e-4	33.0<br>14.8	0.028<br>138	1<br>24	11.6	318<br>375
503.0427	4	-0.61	EAK(+DSS)ASINPVTGR<br>NK(+DSS)LVREAK(+DSS)ASINPVTGR<br>NK(+DSS)LVR	24.83024.830	1	41.7	6.2	4.1e-4	35.5<br>8.6	0.0052<br>312	1<br>47	10.4	449<br>375
639.0385	3	-0.092	EAK(+DSS)ASINPVTGR<br>K(+DSS)FLKEAK(+DSS)ASINPVTGR<br>K(+DSS)FLK	26.30126.301	1	51.7	10.3	4.9e-4	41.4<br>15.5	0.011<br>30	1<br>6	12.5	449<br>349
639.0384	3	-0.25	EAK(+DSS)ASINPVTGR<br>K(+DSS)FLKEAK(+DSS)ASINPVTGR<br>K(+DSS)FLK	26.44326.443	1	62.5	10.3	6.7e-4	52.2<br>12.8	0.010<br>321	1<br>36	12.4	449<br>349
754.1297	3	-0.25	LAK(+DSS)LLVSVLEQGLPPSR<br>K(+DSS)RLAK(+DSS)LLVSVLEQGLPPSR<br>K(+DSS)R	41.96441.964	1	53.9	0.0	0.0013	53.9<br>3.6	0.0013<br>1071	1<br>361	4.2	48<br>44
630.3728	3	-0.13	VLLSFMEK(+DSS)R<br>NK(+DSS)LVRVLLSFMEK(+DSS)R<br>NK(+DSS)LVR	33.76933.769	1	43.2	7.7	0.0021	35.5<br>10.8	0.034<br>269	1<br>66	7.7	318<br>375
473.0309	4	-1.2	VLLSFMEK(+DSS)R<br>NK(+DSS)LVRVLLSFMEK(+DSS)R<br>NK(+DSS)LVR	33.24433.244	1	38.5	7.9	0.0054	30.6<br>6.8	0.087<br>392	1<br>66	7.9	318<br>375

630.3721	3	-1.2	VLLSFMEK(+DSS)R<br>NK(+DSS)LVRVLLSFMEK(+DSS)R<br>NK(+DSS)LVR	33.24933.249	1	40.0	7.8	0.0072	32.2<br>12.2	0.13<br>232	1<br>33	11.9	318<br>375
473.0315	4	0.037	VLLSFMEK(+DSS)R<br>NK(+DSS)LVRVLLSFMEK(+DSS)R<br>NK(+DSS)LVR	34.30534.305	1	40.7	9.4	0.0089	31.3<br>10.1	0.32<br>1011	1<br>80	11.5	318<br>375

Intramolecular DSS-crosslinked peptides of apo Ric8A1-492 were identified by ProteinProspector (v. 5.22.1). Nine nonredundant high-scoring crosslinked pairs are underlined, including four involving residues of the flexible C-terminal region (underlined with green line).

Abbreviations:
RT - peptide elution retention time;
Score - an overall Protein Prospector score that describes how many total product ions are attributable to the entire cross-link;
Score Diff - Difference in score between the top cross-linked match and the top linear match;
Expect - Expectation value for the crosslink;
MS-Tag Score - tag-based scores for the crosslinked peptides;
Exp - Expectation values of stronger and weaker peptides in the crosslink;
Low Score - score calculated after putative cross-linked productshave been assigned and elemental composition is known;
Rank - rank of the individual peptide in the mass modification search;
X-link AA - positions of crosslinked residues
