# Supplementary material for: Structural underpinnings of Ric8A function as a G-protein α-subunit chaperone and guanine-nucleotide exchange factor
Source: Nat Commun. 2019 Jul 12;10:3084. doi: 10.1038/s41467-019-11088-x (PMC6625990; doi:10.1038/s41467-019-11088-x)
Supplement: Supplementary file 6 — Supplementary Data 3 [file 41467_2019_11088_MOESM6_ESM.pdf]

Supplementary Data 3. Intermolecular DSS-crosslinked peptides of the Ric8A1-492/miniGα_i complex

m/z	z	ppm	Crosslinked Peptide	RT	#	Score	Score Diff	Expect	MS-Tag Score	Exp	Rank	Low Score	XLink AA
570.8310	4	0.86	**VK(+DSS)TTGIVETHFTFK**<br>**K(+DSS)FLK**	40.691	1	77.6	2.5	4.0e-8	75.1<br>9.9	9.6e-8<br>665	1<br>473	9.9	61<br>349
1071.8580	3	0.97	**MHESMK(+DSS)LFDSIC(Carbamidomethyl)NNK**<br>**LVNMFDK(+DSS)LSR**	53.618	1	92.0	21.8	8.6e-8	70.2<br>35.8	1.0e-4<br>7.5	1<br>11	23.8	122<br>488
1030.5400	3	1.4	**LK(+DSS)ESVAPVLSVLTEC(Carbamidomethyl)AR**<br>**NNLK(+DSS)DC(Carbamidomethyl)GLF**	60.729	1	73.5	12.5	9.9e-8	57.7<br>10.7	3.6e-5<br>1450	2<br>456	15.8	327<br>223
1030.5400	3	1.4	**LK(+DSS)ESVAPVLSVLTEC(Carbamidomethyl)AR**<br>**NNLK(+DSS)DC(Carbamidomethyl)GLF**	60.737	2	67.8	16.5	1.7e-6	51.2<br>14.9	8.4e-4<br>627	2<br>198	18.3	327<br>223
747.1013	4	0.60	**VEEK(+DSS)PPNPMEGMTEEQK**<br>**EDGEK(+DSS)AAR**	32.037	1	66.0	8.1	2.2e-6	57.9<br>9.4	4.1e-5<br>2022	1<br>968	13.5	462<br>21
747.1016	4	1.00	**VEEK(+DSS)PPNPMEGMTEEQK**<br>**EDGEK(+DSS)AAR**	32.095	1	59.0	10.5	3.3e-6	48.5<br>11.9	2.2e-4<br>523	1<br>508	14.9	462<br>21
474.6113	3	0.61	**SK(+DSS)MIDR**<br>**K(+DSS)FLK**	29.882	1	65.8	16.2	1.3e-5	49.6<br>34.5	0.0016<br>0.16	1<br>2	27.8	9<br>349
474.6118	3	1.7	**SK(+DSS)MIDR**<br>**K(+DSS)FLK**	29.925	1	57.3	16.2	3.9e-5	41.1<br>26.6	0.0080<br>0.93	1<br>2	23.5	9<br>349
474.6113	3	0.61	**SK(+DSS)MIDR**<br>**K(+DSS)FLK**	29.889	1	55.5	14.7	9.0e-5	40.8<br>24.2	0.010<br>2.2	1<br>8	22.1	9<br>349
474.6118	3	1.7	**SK(+DSS)MIDR**<br>**K(+DSS)FLK**	29.940	1	45.0	14.1	4.7e-4	30.9<br>16.2	0.097<br>25	1<br>23	20.4	9<br>349
1071.8580	3	0.97	**MHESMK(+DSS)LFDSIC(Carbamidomethyl)NNK**<br>**LVNMFDK(+DSS)LSR**	53.577	1	63.2	21.2	5.7e-4	42.0<br>19.7	0.40<br>386	1<br>138	21.2	122<br>488
570.8312	4	1.2	**VK(+DSS)TTGIVETHFTFK**<br>**K(+DSS)FLK**	40.765	1	34.8	4.2	0.0012	30.6<br>11.9	0.010<br>147	1<br>136	10.4	61<br>349
479.9427	3	0.10	**SK(+DSS)M(Oxidation)IDR**<br>**K(+DSS)FLK**	25.737	1	58.3	15.7	0.0015	42.6<br>22.1	0.16<br>74	1<br>10	23.1	9<br>349
479.9425	3	-0.31	**SK(+DSS)M(Oxidation)IDR**<br>**K(+DSS)FLK**	25.767	1	49.4	15.1	0.0021	34.3<br>21.1	0.29<br>22	1<br>5	21.4	9<br>349
711.4139	2	1.4	**K(+DSS)FLK**	29.953	1	40.8	11.3	0.0054	21.6	3.7	8	25.5	349

			SK(+DSS)MIDR						18.9	9.3	33		9
711.4134	2	0.73	K(+DSS)FLK	29.914	1	39.0	12.3	0.0054	21.6	3.2	17	23.7	349
			SK(+DSS)MIDR						17.5	14	82		9
474.6115	3	1.0	SK(+DSS)MIDR	30.016	1	44.5	13.1	0.0070	31.4	0.42	1	20.6	9
			K(+DSS)FLK						16.8	40	25		349
474.6115	3	1.0	SK(+DSS)MIDR	30.089	1	44.0	11.5	0.0087	32.5	0.30	1	17.8	9
			K(+DSS)FLK						12.7	132	58		349

Supplementary Data 3. Intermolecular DSS-crosslinked peptides of the Ric8A1-492/miniGα$_i$ complex by ProteinProspector (v. 5.22.1). Five nonredundant crosslinked pairs are underlined, including two involving residues of the distal C-terminal region Ric8A453-492 (underlined with green line).

Abbreviations:
RT - peptide elution retention time;
Score - an overall Protein Prospector score that describes how many total product ions are attributable to the entire cross-link;
Score Diff - Difference in score between the top cross-linked match and the top linear match;
Expect - Expectation value for the crosslink;
MS-Tag Score - tag-based scores for the crosslinked peptides;
Exp - Expectation values of stronger and weaker peptides in the crosslink;
Low Score - score calculated after putative cross-linked productshave been assigned and elemental composition is known;
Rank - rank of the individual peptide in the mass modification search;
X-link AA - positions of crosslinked residues
