# Supplementary material for: Structural underpinnings of Ric8A function as a G-protein α-subunit chaperone and guanine-nucleotide exchange factor
Source: Nat Commun. 2019 Jul 12;10:3084. doi: 10.1038/s41467-019-11088-x (PMC6625990; doi:10.1038/s41467-019-11088-x)
Supplement: Supplementary file 7 — Supplementary Data 4 [file 41467_2019_11088_MOESM7_ESM.pdf]

Supplementary Data 4. Intermolecular DSS-crosslinked peptides of the Ric8A1-492/Gα_t complex

m/z	z	ppm	Crosslinked Peptide	RT	#	Score	Score Diff	Expect	MS-Tag Score	Exp	Rank	Low Score	XLink AA
1035.5400	3	1.6	**LK(+DSS)ESVAPVLSVLTEC(Carbamidomethyl)AR<br>ENLK(+DSS)DC(Carbamidomethyl)GLF**	60.871	1	84.2	13.9	1.9e-7	64.2<br>20.1	1.1e-4<br>141	3<br>64	20.0	327<br>345
1035.5400	3	1.6	**LK(+DSS)ESVAPVLSVLTEC(Carbamidomethyl)AR<br>ENLK(+DSS)DC(Carbamidomethyl)GLF**	60.875	1	67.2	12.6	6.3e-7	50.0<br>18.5	4.3e-4<br>69	3<br>24	17.2	327<br>345
1213.1280	4	1.5	**EIYSHMTC(Carbamidomethyl)ATDTQNVK(+DSS)FVFDAVTDIIIK<br>VLFNITFDSIK(+DSS)R**	66.064	1	81.5	21.5	1.5e-6	53.3<br>29.1	0.026<br>111	3<br>119	28.2	329<br>236
752.3812	4	0.22	**DVK(+DSS)EIYSHMTC(Carbamidomethyl)ATDTQNVK<br>NK(+DSS)LVR**	39.528	1	97.8	12.3	1.8e-6	85.5<br>16.1	4.0e-5<br>1707	1<br>653	18.6	313<br>375
1035.5400	3	1.6	**LK(+DSS)ESVAPVLSVLTEC(Carbamidomethyl)AR<br>ENLK(+DSS)DC(Carbamidomethyl)GLF**	60.936	1	51.5	7.8	1.9e-6	41.0<br>9.5	3.2e-4<br>1456	3<br>589	10.5	327<br>345
970.7027	5	0.27	**EIYSHMTC(Carbamidomethyl)ATDTQNVK(+DSS)FVFDAVTDIIIK<br>VLFNITFDSIK(+DSS)R**	65.989	1	74.5	21.1	1.3e-5	53.4<br>22.4	0.019<br>817	1<br>395	24.4	329<br>236
462.5129	4	1.3	**LK(+DSS)EDAEKDAR<br>K(+DSS)FLK**	24.976	1	55.5	15.6	1.4e-5	39.9<br>26.6	0.0093<br>2.3	1<br>5	22.0	20<br>349
752.3816	4	0.75	**DVK(+DSS)EIYSHMTC(Carbamidomethyl)ATDTQNVK<br>NK(+DSS)LVR**	39.570	1	88.7	11.3	1.6e-5	77.4<br>15.3	2.9e-4<br>2357	1<br>803	18.7	313<br>375
715.1471	4	0.74	**FIK(+DSS)YTGYGNAAGLLAAR<br>STIVK(+DSS)QMK**	44.521	1	86.8	16.6	2.8e-4	70.2<br>16.6	0.012<br>2520	1<br>682	20.8	408<br>47
804.1455	4	1.2	**MHESMK(+DSS)LFDSIC(Carbamidomethyl)NNK<br>LVNMFDK(+DSS)LSR**	53.457	1	79.2	28.1	3.2e-4	50.0<br>35.2	0.81<br>43	2<br>73	29.2	244<br>488
1035.5400	3	1.6	**LK(+DSS)ESVAPVLSVLTEC(Carbamidomethyl)AR<br>ENLK(+DSS)DC(Carbamidomethyl)GLF**	60.877	1	44.5	4.9	3.3e-4	38.3<br>3.0	0.0038<br>4195	3<br>1034	7.3	327<br>345
708.6306	4	0.98	**FIK(+DSS)YTGYGNAAGLLAAR<br>K(+DSS)DLFEEK**	48.467	1	74.8	22.2	0.0011	52.6<br>22.9	0.18<br>159	1<br>123	26.3	408<br>267
462.5133	4	2.2	**LK(+DSS)EDAEKDAR<br>K(+DSS)FLK**	25.028	1	42.8	11.1	0.0013	30.6<br>20.9	0.29<br>21	2<br>5	18.5	20<br>349
708.6302	4	0.41	**FIK(+DSS)YTGYGNAAGLLAAR<br>K(+DSS)DLFEEK**	48.526	1	49.6	12.8	0.0021	36.8<br>10.3	0.16<br>1369	1<br>577	15.0	408<br>267
804.1452	4	0.82	**LVNMFDK(+DSS)LSR<br>MHESMK(+DSS)LFDSIC(Carbamidomethyl)NNK**	53.505	1	57.1	21.1	0.0029	33.8<br>25.2	3.5<br>48	5<br>36	23.3	488<br>244

Suppl. Data 4. Intermolecular DSS-crosslinked peptides of the Ric8A1-492/Gα_t complex by ProteinProspector (v. 5.22.1). Six nonredundant crosslinked pairs are underlined, including the crosslink involving the distal C-terminal region Ric8A453-492 (underlined with green line). For abbreviations see Supplementary Data 3.
